# Mouse tissue glycome atlas 2022 highlights inter-organ variation in major *N*-glycan profiles

**DOI:** 10.1038/s41598-022-21758-4

**Published:** 2022-10-24

**Authors:** Michiru Otaki, Nozomi Hirane, Yayoi Natsume-Kitatani, Mari Nogami Itoh, Masanori Shindo, Yoichi Kurebayashi, Shin-Ichiro Nishimura

**Affiliations:** 1 Field of Drug Discovery Research, Faculty of Advanced Life Science, Hokkaido University, N21, W11, Kita-ku, Sapporo, 001-0021 Japan; 2 Laboratory of Bioinformatics, Artificial Intelligence Center for Health and Biomedical Research, National Institutes of Biomedical Innovation, Health and Nutrition, 7-6-8 Saito-Asagi, Ibaraki, Osaka 567-0085 Japan; 3 Field of Artificial Intelligence and Digital Health Science, Graduate School of Medicine, Kobe University, 1-5-6 Minatojimaminami-cho, Chuo-ku, Kobe, 650-0047 Japan

**Keywords:** Biochemistry, Biological techniques, Chemical biology, Structural biology, Biomarkers, Diseases, Medical research

## Abstract

This study presents “mouse tissue glycome atlas” representing the profiles of major *N*-glycans of mouse glycoproteins that may define their essential functions in the surface glycocalyx of mouse organs/tissues and serum-derived extracellular vesicles (exosomes). Cell surface glycocalyx composed of a variety of *N*-glycans attached covalently to the membrane proteins, notably characteristic “*N*-glycosylation patterns” of the glycocalyx, plays a critical role for the regulation of cell differentiation, cell adhesion, homeostatic immune response, and biodistribution of secreted exosomes. Given that the integrity of cell surface glycocalyx correlates significantly with maintenance of the cellular morphology and homeostatic immune functions, dynamic alterations of *N*-glycosylation patterns in the normal glycocalyx caused by cellular abnormalities may serve as highly sensitive and promising biomarkers. Although it is believed that inter-organs variations in *N*-glycosylation patterns exist, information of the glycan diversity in mouse organs/tissues remains to be elusive. Here we communicate for the first-time *N*-glycosylation patterns of 16 mouse organs/tissues, serum, and serum-derived exosomes of Slc:ddY mice using an established solid-phase glycoblotting platform for the rapid, easy, and high throughput MALDI-TOFMS-based quantitative glycomics. The present results elicited occurrence of the organ/tissue-characteristic *N*-glycosylation patterns that can be discriminated to each other. Basic machine learning analysis using this *N*-glycome dataset enabled classification between 16 mouse organs/tissues with the highest F1 score (69.7–100%) when neural network algorithm was used. A preliminary examination demonstrated that machine learning analysis of mouse lung *N*-glycome dataset by random forest algorithm allows for the discrimination of lungs among the different mouse strains such as the outbred mouse Slc:ddY, inbred mouse DBA/2Crslc, and systemic lupus erythematosus model mouse MRL-*lpr*/*lpr* with the highest F1 score (74.5–83.8%). Our results strongly implicate importance of “human organ/tissue glycome atlas” for understanding the crucial and diversified roles of glycocalyx determined by the organ/tissue-characteristic *N*-glycosylation patterns and the discovery research for *N*-glycome-based disease-specific biomarkers and therapeutic targets.

## Introduction

Glycosylation of proteins is a major posttranslational modification. Many intravital glycoproteins are involved in cell–cell interactions and glycans linked covalently to the proteins have essential roles in numerous biological processes such as cell adhesion, receptor-mediated signaling, immune recognition, and pathogen infection^1^. Membrane tethered proteins are mostly modified covalently with multiple *N*-glycan chains, and these glycan modifications eventually form a bulky forest-like glycocalyx covering the outer layer of the cells^2,3^. Divergent glycan structures and functions of the cell surface glycocalyx may be controlled strictly by the acceptor-substrate specificities of a series of glycosyltransferases and their affinities to the designated sugar nucleotides as donor substrates in the glycoprotein biosynthetic processes in ER/Golgi^4,5^.

It is well documented that specific interactions of the cell surface glycocalyx with endogenous lectins such as galectins, selectins, and siglecs are especially important for various regulatory programs in the homeostatic immune responses related to autoimmunity and neoplastic diseases^6^. In tumor tissues, it has been widely accepted that changes in the cancer cell surface glycocalyx strongly affect the interaction with various lectins and pattern recognition receptors of the neighboring immune cells and stromal cells that may determine the cancer microenvironment^7^.

Cancer cells as well as normal cells generate and release exosomes, nano-sized extracellular vesicles enclosed by the lipid bilayer, that can deliver a variety of cargo molecules including nucleic acids, proteins, and lipids^8^ to the acceptor cells through endocytosis^9^. It was demonstrated that dendritic cells (DCs) and cancer cells induce or reduce the immune responses by generating their exosomes^10^. It was also reported that cancer cell-secreted exosomes remotely promote metastatic tumors by transferring mRNAs involved in metastasis in an organotropic manner^11–13^. Exosomes display various membrane glycoproteins, such as CD9, CD63, CD44, and integrins^14,15^, indicating that surface of exosomes are also covered by the glycocalyx^16^. Surprisingly, artificially designed nanosomes as a simple model for the exosome displaying major *N*-glycans derived from four different human cancer cell lines uncovered that cancer cell-type specific glycocalyx determines immediately circulation, clearance, and organotropic biodistribution of the exosomes secreted from cancer cells independent of the influences of core proteins or other cargo molecules^17^. These results implicate that the glycocalyx of exosomes is closely related to the characteristic *N*-glycosylation patterns of the parent tumor cells involved in the primary cancer tissues. More importantly, dominant interactions between exosomal glycocalyx and endogenous lectins are determined by both thresholds (abundances) of the key glycotypes in the glycocalyx^18^ and the avidity of each lectin receptors on the tissue-resident immune cells such as neutrocytes, monocytes, DCs, macrophages, B cells, natural killer cells (NK), eosinophiles and basophils^19–21^.

Despite emerging importance of the organ/tissue characteristic *N*-glycosylation patterns and their dynamic alterations in the processes of many diseases, the structural features, and expression levels of major *N*-glycans for total glycoproteins in mammalian organs/tissues have remained unclear. Apparently, their biosynthetic processes achieved without genetic template in addition to the structural complexity of glycans have long made systemic and high throughput *N*-glycomics of intact cells and organ/tissue samples extremely difficult^22^. Recent progress in nano-LC and high-performance mass spectrometry-based approaches for in-depth glycomics and glycoproteomics has provided us with numerous excellent tools such as metabolic, isotopic, or isobaric labeling and software for the accurate and quantitative analysis of glycans and glycopeptides^23–25^. However, it is important to note that *N*-glycomics for the diversified and complex mammalian organs/tissues must need multistep, tedious, and time-consuming procedures for the sample preparation before the nano-LC and mass spectrometric analysis, particularly extraction of various glycoproteins, labeling of *N*-glycans released from glycoproteins, isolation and clean-up of the labeled *N*-glycans from highly complicated and heterogeneous mixture containing non-glycan impurities and unknown contaminants^23^. To achieve easy and reproducible *N*-glycan profiling of the samples derived from a variety of mammalian organs/tissues in terms of the differences in size, morphology, and total masses and contents of major glycoproteins for the quantitative *N*-glycomics, we considered that use of the solid-phase chemical manipulations is a promising approach for enabling easy one-pot glycan-enrichment, derivatization, removal of the impurities and contaminants, and accurate quantitation of the organ/tissue-characteristic “*N*-glycosylation patterns”.

Glycoblotting method developed for glycan-specific enrichment makes use of the stability of hemiacetals under mild acidic medium to form oxime or hydrazone bonds with aminooxy- or hydrazide-functionalized solid polymer particles^18,26,27^ that differentiate the reducing glycans (carbohydrates) from any other non-glycan molecules and unknown contaminants existing in generally complex biological sources. By using an established glycoblotting protocol, *N*-glycans captured covalently on a commercially available hydrazide-functionalized solid polymer beads could be washed thoroughly, modified selectively at sialic acid residues, and finally labeled by *trans*-imidization with tagging reagents having an aminooxy group for Mass Spec Quantitative Analysis^27^. Merit of the glycoblotting technique is evident because a streamlined on-bead-one-pot glycan enrichment and chemical manipulation allowed easy, reliable, and high throughput quantitative profiling of major *N*-glycans released from gross glycoproteins of a variety of human clinical samples of various diseases such as cancers^28–33^, ulcerative colitis^34^, diabetic retinopathy^35,36^, osteoarthritis^37,38^, and Alzheimer’s disease^39^ as well as *N*-glycome profiling of the cultured mammalian cells^18,40,41^.

In the present study, we challenge the construction of the first database for mouse organ/tissue *N*-glycome by using glycoblotting-based solid-phase platform that facilitates rapid, systemic, and quantitative profiling of organ/tissue-characteristic *N*-glycosylation patterns. We considered that annotation of *N*-glycomics dataset for mouse, one of the most important standard and disease model organisms, provides substantial challenges in the integrative approach to utilize the multi-omics datasets to gain insights into biological systems. Indeed, mouse has well-annotated genomes, transcriptomes and proteomes, and many tools available for interactive annotation^42–44^. Preliminary machine learning analysis demonstrates advantage and importance of the comprehensive *N*-glycome database for widely used mouse organs/tissues, serum, and exosomes in the investigation of basic biological functions of organ/tissue specific *N*-glycosylation patterns and the discovery research of novel *N*-glycome-related diagnostic biomarkers and therapeutic targets in various human diseases.

## Results

### Glycoblotting-based solid-phase approach is well-suited for global *N*-glycan profiling of mouse organs/tissue, serum, and exosomes

**Figure 1:**
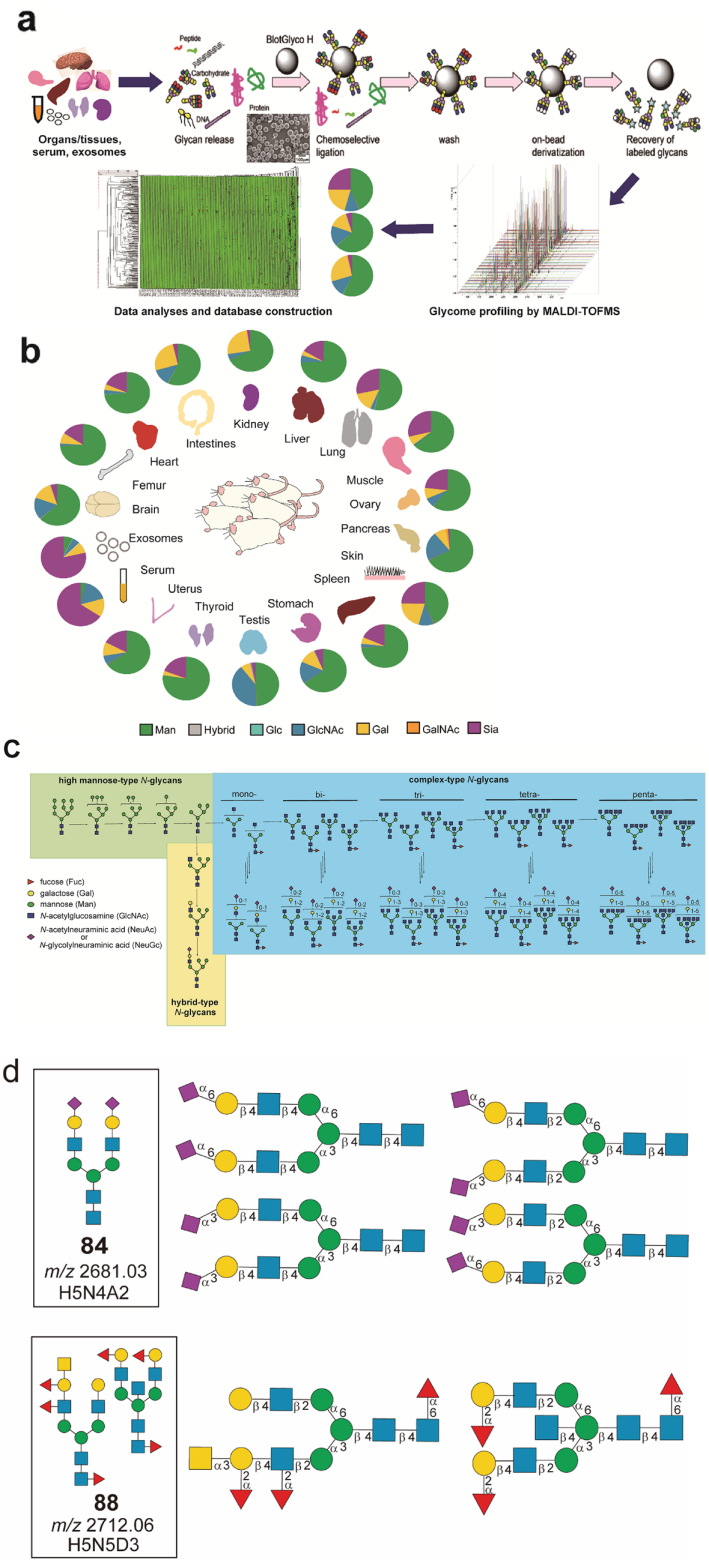
A strategy for the construction of the mouse glycome atlas. (**a**) An image representation for the glycoblotting-based solid-phase glycan enrichment analysis for the construction of mouse tissue glycome atlas. (**b**) In the present study, sixteen frozen organs/tissues in addition to the serum and serum-derived exosomes were employed for the general protocol enabling one-pot rapid and efficient glycan enrichment and subsequent chemical manipulations on the polymer-solid beads. Pie charts represent ratio of the terminal sugar residues of each organ revealed by glycotyping analysis^17,18,37,49^ of the present results. (**c**) General biosynthetic pathway of major *N*-glycans showing maturation from high mannose-type to hybrid-type and complex-type glycoforms in the posttranslational modification of mammalian proteins. This schematic representation does not involve the biosynthesis of minor glycoforms having GalNAc terminals, Lewis type antigenic structures, lactosaminoglycans (LacNAc repeats), sulfated sugar moieties, and so on. (**d**) The sugar compositions of *N*-glycans assigned based on the reported structures may often predict multiple candidates of the isomeric glycoforms found in the Expasy GlycoMod Tool (https://web.expasy.org/glycomod/ and https://glyconnect.expasy.org/browser/compositions/453).

Global profiling of characteristic *N*-glycosylation patterns for 16 mouse organs/tissues and body fluids (brain, femur, heart, intestines, kidney, liver, lung, muscle, ovary, pancreas, skin, spleen, stomach, testis, thyroid, uterus, serum, and serum-derived exosomes) of healthy normal mice (outbred strain Slc:ddY) was performed efficiently by using an improved glycoblotting-based *N*-glycomics workflow shown in Fig. 1a. It is important to note that the pretreatment process is easy, reproducible, and generalizable for any animal organ/tissue samples used in the common biological experiments. We established herein a standardized method for the mammalian tissue sample preparation as follows: (i) all frozen organs/tissues collected by surgical dissection of five perfused mice were lyophilized and grounded before use, whereas thyroid and serum were collected from the pooled samples; (ii) a small spatula of the grounded powdery samples (approximately 2–3 mm^3^) was dissolved in 100 mL of lysis buffer (0.1% SDS, 0.1% Triton X-100 in 100 mM ammonium hydrogen carbonate) to extract proteins under sonication. After centrifugation, the supernatant containing total proteins was quantified by using BCA method and estimated to be a range from 0.418 (femur) to 2.89 (liver) mg; (iii) an extract solution, 23.6 (liver)–3.46 (femur) mL, containing 100 mg of proteins was subjected directly without reductive alkylation and tryptic digestion to the treatment with 200 U of PNGase F at 37 °C for 16 h, in which the solution volume was adjusted finally to 50 mL by using lysis buffer; (iv) the crude materials containing released *N*-glycans were employed for the glycoblotting and solid-phase manipulation with BlotGlyco® H beads (2 mg in 200 mL) in the presence of 10 pmol of non-natural *N*-glycan derivative [(Neu5Aca2,6Galb1,4GlcNAcb1,2Man)_2_a1,3/6Manb1,4GlcNAc] as an internal standard according to the well-established protocol for human serum *N*-glycan profiling^28–34^. Exosomes were prepared from the pooled mouse serum (~ 25 mL) by a general procedure using ultracentrifugation^45^. The isolated exosomes were found to be the size in a range from 50 to 250 nm (Fig. S1a) and displayed the typical exosomal biomarkers such as CD9 and CD81 (Fig. S1b)^46^. The exosomal protein concentration was estimated to be 18.6 mg/ mL of serum. Serum-derived exosomes (corresponds to 10 mg of total proteins) can be subjected directly to the step (iii), the treatment with PNGase F as described above.

Solid-phase glycoblotting and subsequent chemical derivatization of covalently enriched *N*-glycans allowed for the first time easy and quantitative MALDI-TOFMS-based *N*-glycomics of mouse organs/tissues, serum, and exosomes (n = 5) (Fig. 1b). Totally, 103 monosaccharide compositions of the *N*-glycans were predicted basically by using the Expasy GlycoMod Tool (https://web.expasy.org/glycomod/ and https://glyconnect.expasy.org/browser/compositions/453) that covers large datasets for the molecular weight (*m/z*) due to the *N*-glycans observed in mass spectrometry as summarized in Tables S1, S2, and Fig. S2, while the *N*-glycan compositions listed herein do not cover considerable numbers of the isomeric variants that could be produced by the position-specific a2,3- and a2,6-sialylation at Gal residues^47,48^. In addition, each molecular weight data may be a mixture of these glycoforms. In the present study, we allowed to use the mass peaks corresponding to the known mammalian *N*-glycan structures detected in at least three independent experiments throughout all organs/tissues, serum, and exosomes (Table S2). Grouping of the *N*-glycan compositions by such as glycotypes, number of the branch, and the key terminal sugar residue described in Table S1 was based on the hierarchical *N*-glycan structures elaborated by the general mammalian biosynthetic pathway (Fig. 1c). All of the datasets in terms of the raw mass spectra, molecular mass (*m/z*), predicted glycoforms (compound number in bold) and compositions indicated by single-letter-representation [H = Hex (Man, Gal, Glc), N = HexNAc (GlcNAc, GalNAc), D = deoxy Hex (Fuc), A = Neu5Ac, and G = Neu5Gc] as exemplified in Fig. 1d, and absolute quantities (0.04–903.13 pmol/100 mg total protein) determined by means of the spiked internal standard were summarized in Fig. S3 and Table S2, respectively. It is important to note that the monosaccharide compositions of each predicted glycoform assigned based on the reported *N*-glycan structures in the database would predict plural isomeric structures as plausible candidates (Fig. 1d). For example, a well-known biantennary glycoform **84** (H5N4A2) having two Neu5Ac residues may represent at least four possible isomeric structures that could be produced by the regio-specific sialylation at two Gal residues, notably whether a2,3 or a2,6-glycoside linkage, respectively. On the other hand, a complex glycoform **88** (H5N5D3) bearing three Fuc residues identified herein for the first time in mouse can be predicted as two completely different biantennary (left) and bisect (right) structures reported previously in human, sheep, and pig as reported in the Glyconnect database (https://glyconnect.expasy.org/browser/compositions/453).

**Figure 2:**
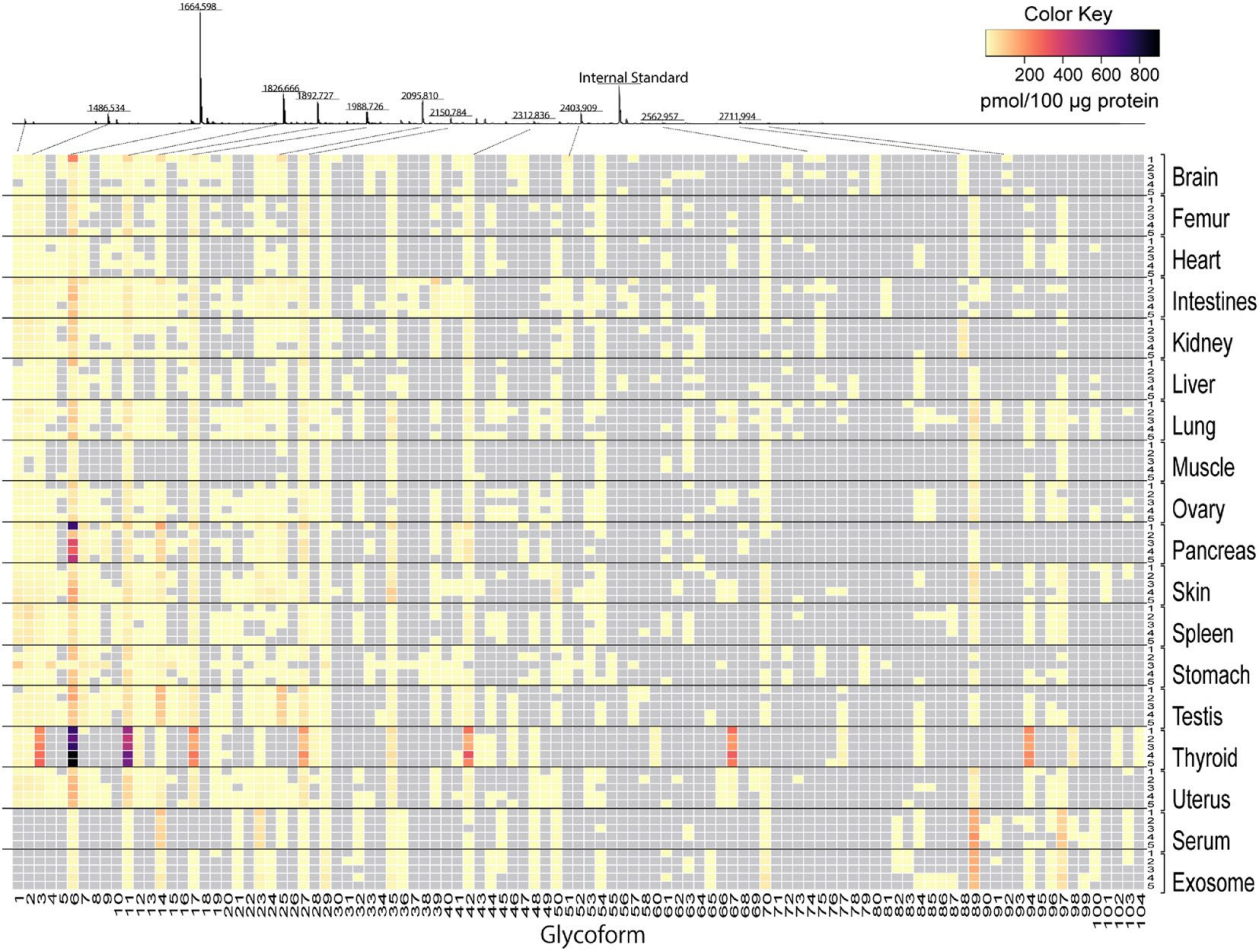
Heatmap representation of the absolute expression levels (pmole/100 mg total protein) of *N*-glycan structures (103 glycoforms listed in Fig. S3) identified in the mouse organs/tissues, serum, and serum-derived exosomes. The MALDI-TOFMS spectrum is a result for one of the five samples of the brain samples tested. The heatmap shows an absolute glycan level estimated from the peak areas compared with that of the internal standard spiked. The grey rectangles show that the peaks are not detected. The numbers of 1–5 in a right column show the sample number for each organ/tissue, serum, and exosomes described in the Table S2. The numbers of **1**–**104** (compound **59** is an internal standard spiked before glycoblotting) in a bottom row represent the glycoforms listed in Table S1 and Fig. S2, respectively.

**Figure 3:**
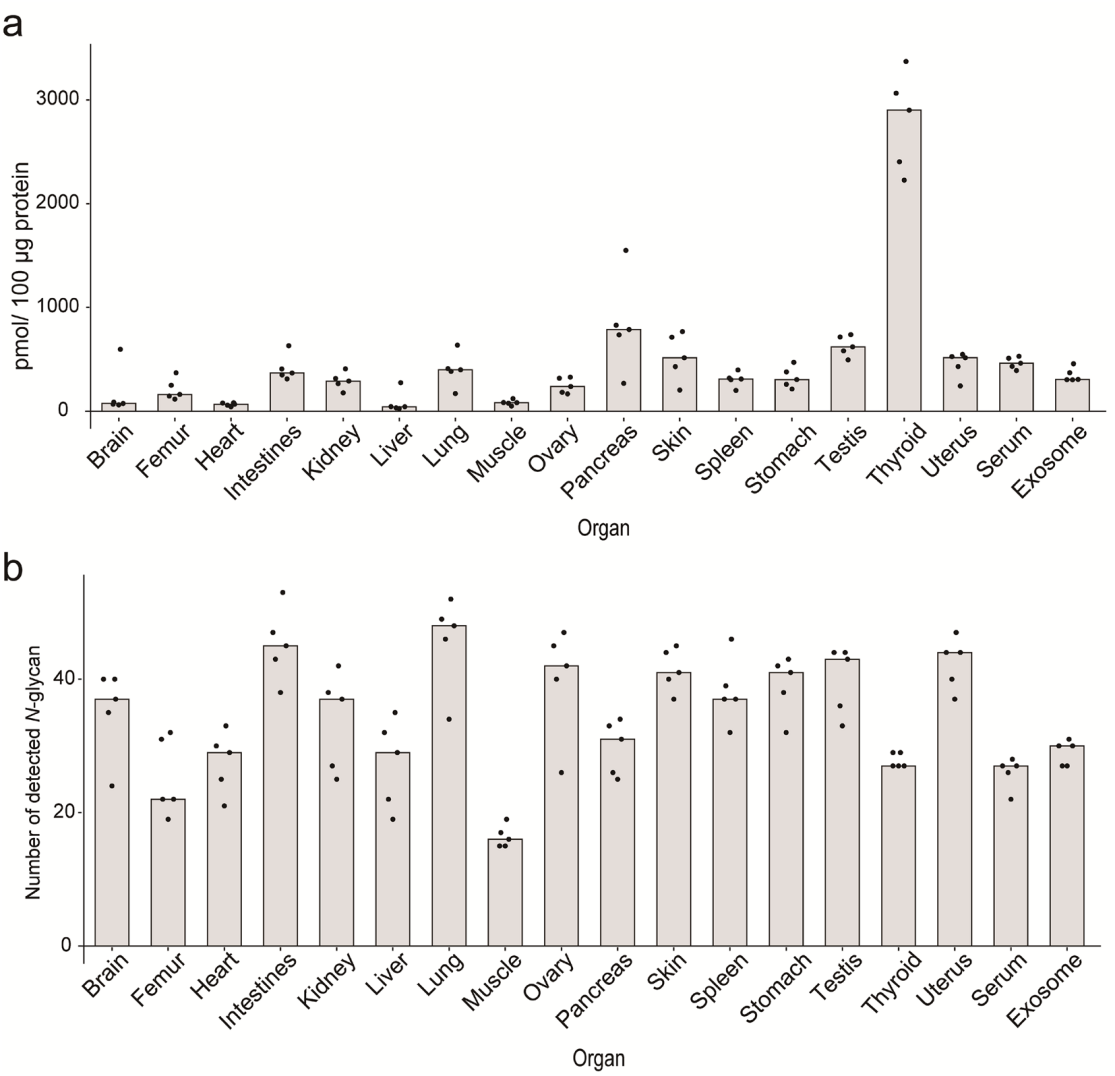
Posttranslational protein glycosylation is dependent strongly on the individual organs. (**a**) Differences in the expression levels of gross *N*-glycans represented as pmole/100 mg protein. (**b**) Total number of *N*-glycans (glycoforms) identified in the organs/tissues, serum, and exosomes, respectively. Column charts indicate the medians of the results for five independent experiments.

An overview of the mouse *N*-glycome atlas revealed that there are significantly organ/tissue-specific features in the *N*-glycosylation patterns determined mainly by the numbers and abundances of some distinct *N*-glycan structures (predicted glycoforms) as indicated in the heatmap representation (Fig. 2). In this context, it seems likely that total expression levels of the total *N*-glycans (Fig. 3a) and total number of the identified *N*-glycans (Fig. 3b) also implicate remarkable inter-organ differences in the total *N*-glycome profiles. Surprisingly, the *N*-glycosylation pattern of muscles was composed only of 16 glycoforms with the lowest total expression level (82.8 pmol/100 μg protein). In contrast, thyroids, and pancreas contained much higher densities of *N*-glycans (2901.0 and 787.0 pmol/100 μg protein) than other organs, whereas totally 27 and 31 glycoforms were profiled, respectively. These results show highly abundant specific *N*-glycan structures in thyroids and pancreas.

### Highlighting inter-organ differences in mouse N-glycome profiles

The heatmap representation of mouse organ/tissue *N*-glycome profiles, notably “barcodes” of the absolute expression levels of each *N*-glycan composition, clearly elicited presence of some abundant *N*-glycans (glycoforms) that can differentiate these organs/tissues, serum, and exosomes (Fig. 2, see also, Table S1 and Fig. S2). For example, thyroids may be characterized specifically by the *N*-glycosylation pattern having highly abundant many glycoforms such as **3** (H4N2), **6** (H5N2), **11** (H6N2), **17** (H7N2), **27** (H8N2), **42** (H9N2), **67** (H5N4D1A1), and **94** (H5N4D1A2), respectively. This result suggests that gross glycoproteins distributed in thyroids on the average are modified heavily by six of mannose terminated *N*-glycans and two sialylated *N*-glycans with fucose residue. The barcode for testis is also differentiated easily by focusing on the three highly abundant glycoforms, **6** (H5N2), **14** (H3N4D1), and **25** (H3N5D1). On the other hand, pancreas can be discriminated from other organs by the unique barcode containing highly abundant glycoform **6** (H5N2) in addition to other abundant **14** (H3N4D1), **17** (H7N2), **27** (H8N2), and **36** (H4N4G1). Interestingly, mouse serum was found to have quite different *N*-glycosylation pattern from those of other organs/tissues that can be discriminated by using some abundant and unique glycoforms such as **14** (H3N4D1), **23** (H4N4D1), **32** (H5N3G1), **45** (H6N3G1), **89** (H5N4G2), and **97** (H5N4D1G2).

**Figure 4:**
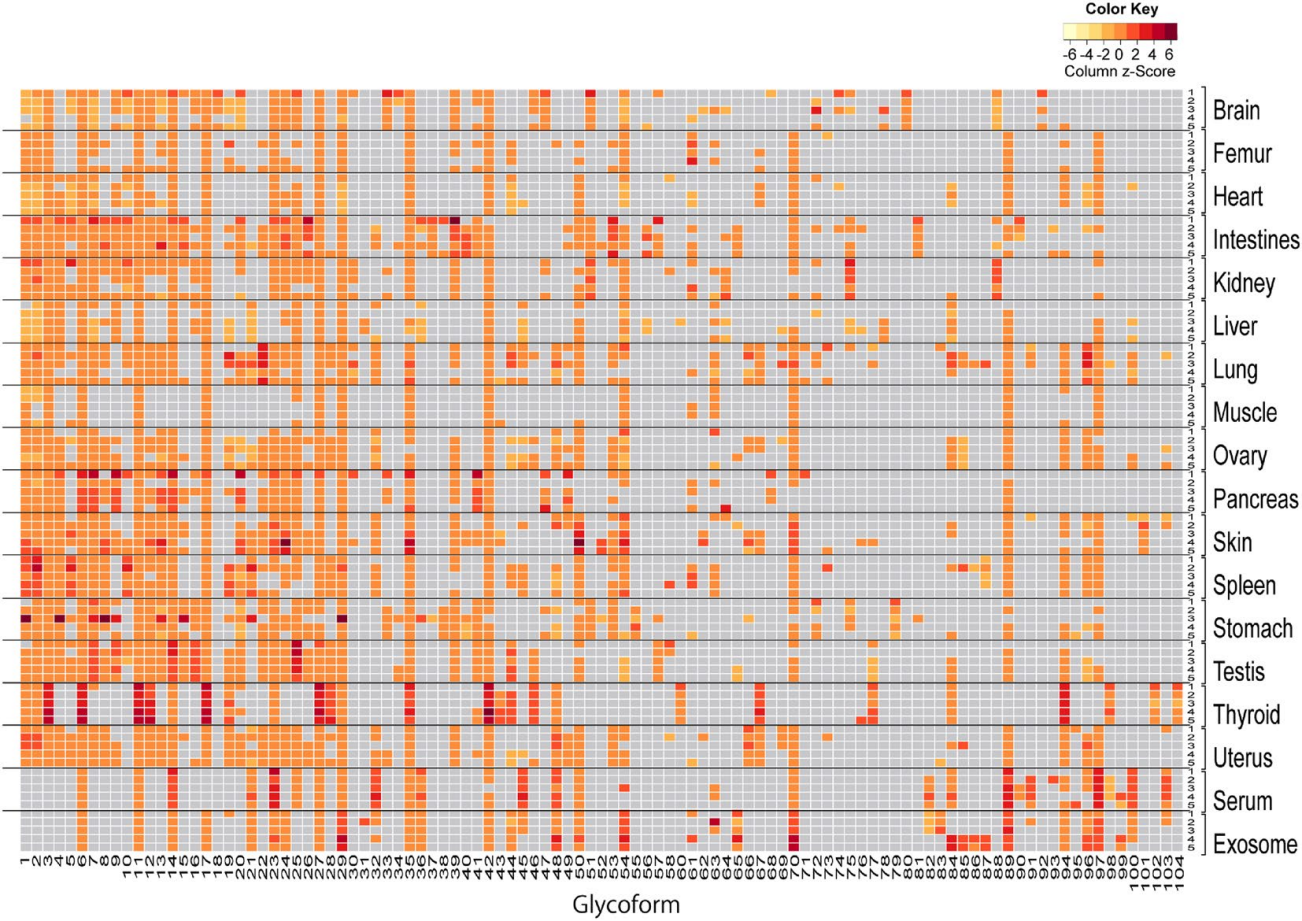
Heatmap representation of the scaled expression levels of *N*-glycan structures shown in Fig. 2 (103 glycoforms listed in Fig. S2) identified in the mouse organs/tissues, serum, and serum-derived exosomes. The color key in the heatmap shows the column z-score of level for each glycoform. The grey rectangles show that the peaks are not detected. The numbers of 1–5 in a right column show the sample number for each organ/tissue, serum, and exosomes described in the Table S2. The numbers of **1**–**104** (compound **59** as an internal standard is not shown) in a bottom row represent the glycoforms listed in Table S1 and Fig. S2, respectively.

To better understand both inter- and intra-organ differences in the expression levels of the individual glycoforms, a scaled heatmap was reconstructed to highlight the deviation of *N*-glycosylation patterns including relatively lower abundances of glycoforms (Fig. 4), while the heatmap of Fig. 2 shows differences in the absolute abundance of 103 N-glycans in a wide dynamic range from 0.04 to 903.13 pmol/100 mg total protein. Obviously, this heatmap provides much more informative barcodes than those shown in Fig. 2, in which the heatmap represented by the scaled *N*-glycome expression levels unveiled newly many organ-specific features in the *N*-glycosylation patterns of brain, intestines, kidney, skin, spleen, stomach, uterus, serum, and exosomes. For example, brain is characterized by the specific *N*-glycosylation pattern containing bisecting type glycoforms with Fuc residues such as **25** (H3N5D1), **51** (H4N5D2), **80** (H4N5D2), and **88** (H5N5D3), respectively. Interestingly, our previous study had also revealed independent presence of the glycoforms containing bisecting type structures such as **16** (H3N5), **25** (H3N5D1), **51** (H4N5D2), and **88** (H5N5D3) as major *N*-glycan structures in the transgenic mouse brains of Huntington’s disease model^41^. It was revealed that intestines are characterized efficiently by the unique multivalent and fucosylated glycoforms **41** (H3N6D1), **53** (H5N5D1), **65** (H4N7), and **81** (H5N6D1). Moreover, kidney can be discriminated from other organs by occurrence of two unique glycoforms **75** (H5N5D2) and **88** (H5N5D3) involving multiple fucosylations (Fig. 1d). Surprisingly, glycoform **101** (H6N5G2) terminated with two Neu5Gc residues was found to be specifically identified only in the skin samples, while highly complex glycoforms containing both Fuc and Neu5Ac residues such as **60** (H3N4D3A1), **98** (H6N5D1A1), **102** (H6N5D1A2), and **104** (H6N5D1A3) were observed only in the thyroid tissue samples. Spleen appeared to have distinct *N*-glycosylation pattern from those of other organs when focusing on the glycoforms **2** (H3N2D1), **5** (H4N2D1), **10** (H5N2D1), **19** (H4N3A1), and **21** (H4N3G1). Interestingly, stomach and uterus can be discriminated specifically from other organs by the occurrence of glycoforms **79** (H3N6D3) and **66** (H4N4A2), respectively. Mouse exosomes derived from serum can be easily differentiated from serum by highlighting the glycoforms **17** (H7N2), **44** (H6N3A1), **54** (H6N5), and **65** (H4N7) that are not detected in the serum samples.

### Glycotyping and clustering analysis of mouse organ/tissue N-glycan compositions

**Figure 5:**
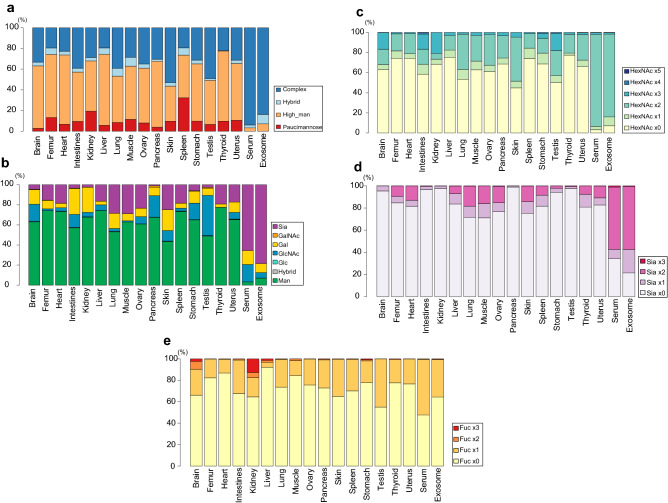
Glycotyping analysis of 103 mouse organ glycoforms based on the general *N*-glycan taxonomy (**a**), the most matured terminal sugar residue defined by the hierarchy in *N*-glycan biosynthesis (**b**), the numbers of HexNAc except core structure or the numbers of antennae of the *N*-glycan structure (**c**), number of sialic acid residue (Neu5Ac and Neu5Gc) (**d**), and number of Fuc (**e**), respectively. Colored columns show the percentages (average of five samples) to total *N*-glycans estimated in the individual organs except the internal standard compound spiked.

Classification of complex and manifold *N*-glycan structures into the simple groups by highlighting some common structural motifs in mammalian *N*-glycans may assist our insight into the organ characteristic features in the *N*-glycosylation patterns^18,37,49^. Glycotyping analysis of totally 103 of *N*-glycan compositions identified herein mouse 16 organs/tissues, serum, and exosomes (Tables S1, S2, S3) was examined by focusing on general glycan types (Fig. 5a), dominant terminal sugars (Fig. 5b), number of HexNAc (Fig. 5c), number of sialic acids (Fig. 5d), and number of Fuc (Fig. 5e), respectively. These analyses were based on a speculation that the same *m/z* contains a single major glycoform, not a mixture of isoforms. As shown in Fig. 5a, it is important to note that major glycoproteins in the mouse serum and exosomes are modified extensively with complex type *N*-glycans (> 80%). In contrast, it was also revealed that the *N*-glycan compositions of 16 organs/tissues are composed of distinctly high levels of high mannose-type *N*-glycans (40–70%), while the ratio of glycotypes for the organ/tissue glycoproteins differs significantly among these organs/tissues. Intriguingly, Fig. 5a also showed that glycoproteins in spleen have unusually higher-level (~ 20%) of paucimannose *N*-glycans **1** (H3N2) and **2** (H3N2D1) than others. The ratio of terminal sugars (Fig. 5b) and the number of HexNAc residues (Fig. 5c) seemed to correlate strongly with the branching number terminated with sialic acid residues of the abundant glycoforms (Fig. 5d). On the other hand, glycotyping by the number of Fuc uncovered remarkably higher-level expression of *N*-glycans containing multiple Fuc residues such as **75** (H5N5D2) and **88** (H5N5D3) in the kidney when compared with other organs (Fig. 5e).

**Figure 6:**
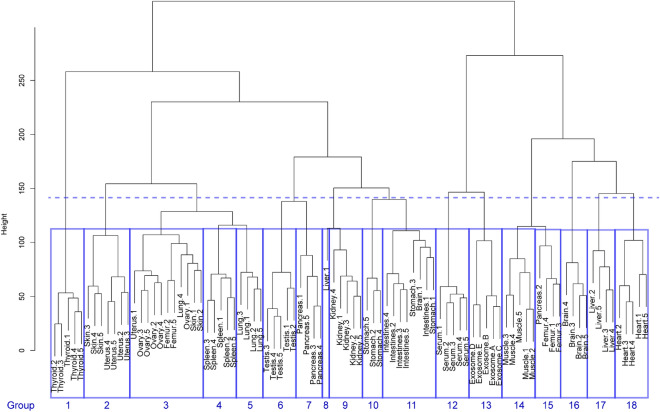
The hierarchical clustering analysis of the mouse *N*-glycome datasets. The *N*-glycosylation pattern dissimilarities in all samples were aggregated into 18 groups by using hierarchical clustering method (unsupervised learning). The Canberra distances were used as the dissimilarities among the *N*-glycosylation patterns in the samples. The dendrogram represents the aggregation by Ward’s method.

Clustering analysis is often useful for dividing a large group showing the diversified *N*- glycome profiles into smaller subgroups that share unique patterns in the sources for the genetically quite similar organisms having remarkable inter-species differences in the body size, lifestyles, and diet as demonstrated in the avian egg white glycoproteins^50^, and the hierarchical clustering tool groups records and arranges them in a dendrogram based on their similarity. The hierarchical clustering analysis of the mouse *N*-glycome datasets, the molecular mass (*m/z*) and expression levels (pmole/100 mg protein) of 103 glycoforms for totally 90 samples tested (Tables S2 and S3), was carried out based on Canberra distance among the organs/tissues, serum, and exosomes because this approach is sensitive to the small changes when both coordinate near to zero and is not sensitive to the outliers. As a result, eight of 18 groups (Group 1, 4, 6, 9, 12, 13, 14, and 18) were summarized by organ, serum, or exosomes (Fig. 6). In addition, the dendrogram demonstrated that ovary (Group 3), spleen (Group 4), testis (Group 6), kidney (Group 9), intestines (Group 11), muscle (Group 14), and heart (Group 18) are in the respective groups regardless of the variance derived from individual differences of the *N*-glycosylation patterns. However, femur, skin, and some outliers were not discriminated to other organs in this analysis (Group 2, 3, 8, 11, and 15). The dashed line shows that the *N*-glycosylation patterns of ovary, spleen, and lung (Group 3, 4, and 5), testis and pancreas (Group 6 and 7), and stomach and intestines (Group 10 and 11) were similar among organs. These results suggest that clustering analysis may facilitate partly quantitation of the inter-organs distance regarding their similarity/dissimilarity in the total *N*-glycome profiles.

### Machine learning approach expands applicability of the mouse glycome atlas

Digital information for multi-omics data such as genomics/transcriptomics, proteomics and metabolomics has recently become widely available for the discovery of novel biomarkers which can provide surrogate information for the presence of a disease and its condition^51^. Machine learning approaches and data mining tools have greatly supported the growth in the development of precision medicine toward customized healthcare^44^. Especially, merging the information from different omics techniques^52^ into valid datasets by machine learning approach is one of the most exciting and important challenges in precision medicine as well as basic systems biology. Despite the increasing importance of the *N*-glycomics as a potential and unique omics dataset that may provide various disease specific and highly sensitive biomarkers, availability of human *N*-glycome data in the bioinformatic studies remains still very low. We hypothesized that the use of machine learning of organ/tissue-characteristic *N*-glycosylation patterns might facilitate more efficient classification of the complex and diversified *N*-glycome profiles in the mouse organs/tissues, serum, and exosomes than other approaches. Considering the biosynthetically defined diversity (Fig. 1c) of the functional *N*-glycan compositions (103 glycoforms, Fig. S2) and dynamic range both of molecular weights (*m/z* 1340.55–3497.33, Table S1) and absolute abundances (0.04–903.13 pmol/100 mg total protein, Tables S2 and S3), *N*-glycomics data required for defining organ/tissue-specific *N*-glycan compositions can be apparently simpler and smaller size than other omics data with ultra-high dimensional datasets^44,53^. Therefore, it was expected that a certain number (scale) of the samples might allow for utilizing machine learning approaches without the risk of overlearning. Our attention was directed to the potentials of the mouse tissue glycome atlas in the machine learning-based analysis, while some algorithms may not provide accurate results due to the insufficient numbers of the present *N*-glycomics data.

**Figure 7:**
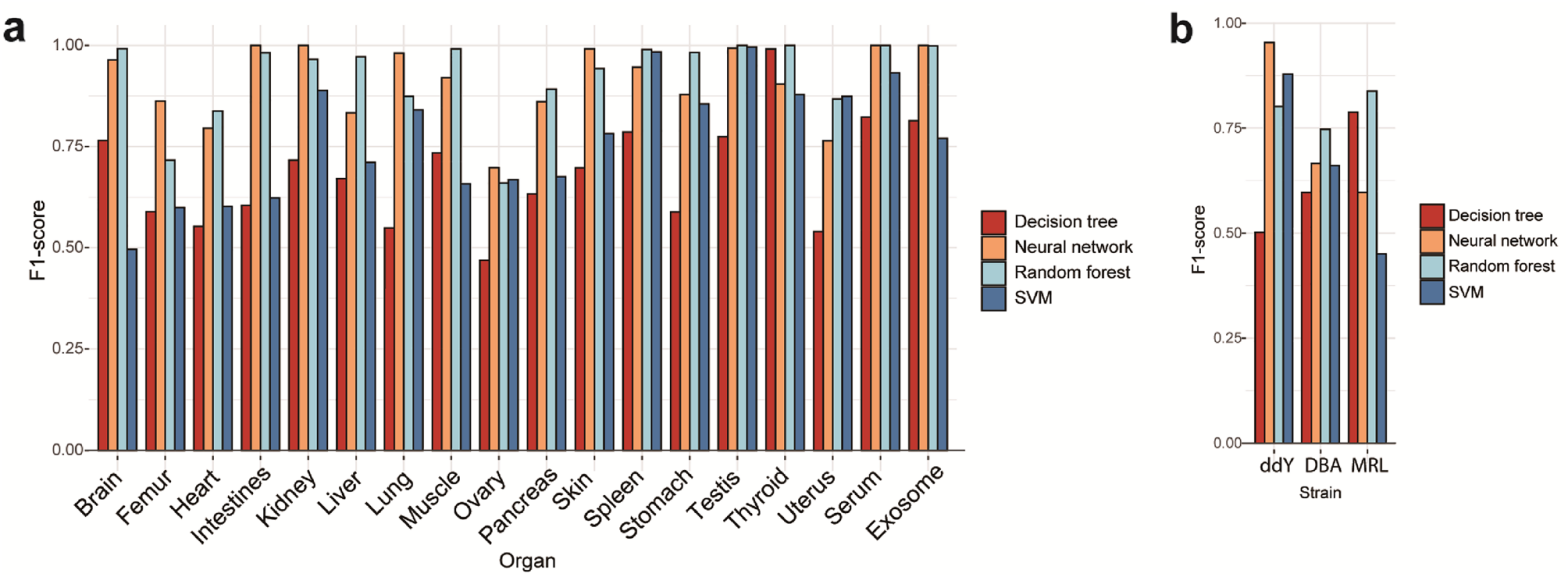
Machine learning analysis of the organ *N*-glycosylation patterns expands applicability of the mouse tissue glycome atlas. (**a**) The F1 scores in predicting organs by discriminant analysis using four machine learning algorithms with organ/tissue *N*-glycan profiles. The test data were randomly selected one of five replicates by stratified sampling, while other four of five samples were used as learning data. (**b**) The F1 scores in predicting mouse strains by discriminant analysis using four machine learning algorisms with lung *N*-glycan profiles (Tables S2 and S4). Red: decision tree, orange: neural network, sky-blue: random forest, blue: SVM. ddY: outbred mouse Slc:ddY, DBA: inflammatory inbred mouse DBA/2Crslc, MRL: the model mouse of systemic lupus erythematosus (SLE) syndrome MRL-lpr/lpr.

To test this hypothesis, we trained a multi-class classifier using four different machine learning algorithms such as decision tree, neural network, random forest, and support vector machine (SVM) in the *N*-glycosylation pattern-based classification of the mouse organs/tissues, serum, and exosomes. Four of the five records for each organ *N*-glycome data were randomly selected as training data to learn *N*-glycosylation patterns of the individual organs. Each model was evaluated on the training data to be able to simultaneously predict classification for each of the organs. The training and prediction process was repeated more than 1000 times, selecting different combinations of training and test data to obtain the F1 score as shown in Fig. 7a. These results showed that neural network algorithm provides the highest F1 score (69.7–100%) among four algorithms tested. The averages of F1 scores across all organs were 68.3 ± 3.0% in decision tree, 91.1 ± 2.2% in neural network, 76.9 ± 3.4% in SVM, and 92.6 ± 2.3% in random forest (mean ± SE).

Versatility of this approach was demonstrated by the discrimination of three mouse strains, outbred mouse Slc:ddY, inflammatory inbred mouse DBA/2Crslc^54,55^, and MRL-*lpr*/*lpr* mice^56,57^ known as a disease model mouse of SLE syndrome^58^, based on the differences in the lung *N*-glycosylation pattern according to the above examination protocol for the machine learning algorisms. Although the purpose of our study by means of these disease model mouse strains is to discover novel glycan-related biomarkers in various lung inflammation (idiopathic pulmonary fibrosis), infection, cancer, and autoimmune disease (https://www8.cao.go.jp/cstp/panhu/prism2021_e/2021.html), it was thought that this preliminary assessment may implicate general potentials, merits, and feasibility of our approach based on the mouse tissue glycome atlas in a variety of studies using designated model mice. *N*-glycosylation patterns of the lungs (n = 5) for DBA/2Crslc and MRL-*lpr*/*lpr* mice were profiled according to the general procedure shown in Fig. 1 and the results (Table S4) were employed for the machine learning study in combination with the dataset of the lung *N*-glycosylation pattern for the outbred mouse Slc:ddY (Table S2). As shown in Fig. 7b, it was demonstrated that the random forest algorithm provides 74.5–83.8% of F1 score for the discrimination of three mouse strains. The averages of F1 scores in three strains were 62.8 ± 8.4% in decision tree, 73.9 ± 10.9% in neural network, 66.3 ± 12.4% in SVM, and 79.5 ± 2.6% in random forest (mean ± SE). The present results implicate high potentials of mouse tissue *N*-glycome datasets in the discrimination of the organs/tissues between normal and disease model mice, and further discovery study towards *N*-glycome-related biomarkers.

## Discussion

Mice are the predominant and the most used experimental model in basic and biomedical research because the similarity in the genetic code between mice and humans is considerably high with synteny of over 80%^43^. Indeed, we use laboratory mice to answer a variety of questions ranging from investigating a pathological effect of knocked out/in gene to understanding drug efficacy and metabolism. Importantly, these studies using mouse models need essentially the high-throughput molecular phenotyping techniques such as genetics, proteomics, and metabolomics, which may provide systemic and comprehensive insights into the molecular mechanisms in the related human diseases^44,51–53^. Integrating multi-omics data by artificial intelligence would accelerate the development of precision medicine as well as biological markers which can identify the molecular cause of a disease, although machine learning analysis of multi-omics seems to be still in the embryonic stage.

Merit of the present approach is evident because quantitative profiling of the *N*-glycosylation patterns allows for the discrimination of the organs/tissues between normal and disease model mice. Remarkably, 48 out of 103N-glycans profiled in this study (Fig. S2) were found to be identified for the first time as mouse-derived *N*-glycans, while they have been reported and listed as human and/or other mammalian *N*-glycome data in the Expasy GlycoMod Tool (https://web.expasy.org/glycomod/ and https://glyconnect.expasy.org/browser/compositions/453). Interestingly, some *N*-glycans found in the serum-derived exosomes could not be detected in the serum samples, implicating the potentials of exosomal glycocalyx as organ/tissue-specific biomarkers. Obviously, our results provided evidence that inter-organs variations in *N*-glycosylation patterns exist in mice. The standardized protocol established herein may facilitate the development of a novel database that covers organ/tissue *N*-glycome profiles of a variety of disease model mice contributing to the discovery of the organ/tissue-specific *N*-glycosylation patterns as new class of biomarkers in human diseases. Indeed, the use of machine learning of organ/tissue-characteristic *N*-glycosylation patterns facilitated classification of the complex and diversified *N*-glycome profiles in the mouse organs/tissues, implicating that machine learning approach may expand applicability of the mouse tissue glycome atlas toward the discovery of the novel biomarkers. However, it should be noted that we need to increase the number and quality of datasets of the organ/tissue/exosome *N*-glycome profiles for various disease model mice including information on the isomeric variants produced by the position-specific a2,3- and a2,6-sialylation at Gal residues^47,48^.

Yamakawa et al. reported organ-specific sialylation and glycosylation patterns in zebrafish^59^, in which glycan structures were profiled systematically by the combined use of MALDI-TOFMS, GC/MS, and NMR. It is interesting to note that comparative study of organ-specific *N*-glycosylation patterns between fish and mammals may be of benefit from an evolutional aspect. On the other hand, mouse organ *N-*glycome was profiled efficiently by using lectin microarray^60^, whereas lectins recognize mostly the terminal sugars of *N-* and *O-*glycans. Given that some MS-based *N-*glycome analyses in the specific mouse organ/tissue have also been reported previously^61,62^, efforts for integrating various mouse tissue *N*-glycome datasets obtained by different methodologies would also be crucial for improving the quality and versatility of glycome databases.

Recently, Borchers and coworkers developed over 5000 mass spectrometry-based targeted proteomics assays for 20 mouse tissues and determined the concentration ranges of a total of over 1600 proteins^63^. It is interesting to note that targeted quantitative glycoproteomics approach^64^ for these mouse tissue proteins might provide the concentration ranges of the glycoproteins as highly potential biomarkers containing information of the *N*-glycoforms modified at each *N*-glycosylation site. However, it is now clear that interactions between organ/tissue glycocalyx and endogenous lectins and pattern recognition receptors depend strongly on the *N*-glycosylation patterns of each organ/tissue glycocalyx, notably the abundances of the key glycoforms^17,18^. In other words, it is likely that glycans can function as clustered molecules, in which cell surface density of the key glycoforms in the individual organs, tissues, and exosomes may be essential for determining the specificity and affinity strength in the interaction with various partner molecules having carbohydrate recognition domains. It was reported that damages in the glycocalyx integrity disrupt normal cellular functions, leading to organ/tissue dysfunction and severe diseases^65,66^.

## Conclusion

The present study communicates the “mouse tissue glycome atlas” representing the profiles of *N*-glycosylation patterns of total proteins that may define their essential functions in the *N*-glycosylation patterns of mouse organs/tissues, serum, and serum-derived exosomes. It is clear that cell surface glycocalyx composed of the characteristic “*N*-glycosylation patterns” plays a critical role for the regulation of cell differentiation, cell adhesion, homeostatic immune response^67^, and biodistribution of secreted exosomes^17^. It is important to note that the integrity of cell surface glycocalyx correlates strongly with maintenance of the cellular morphology and homeostatic immune responses. Therefore, changes in the *N*-glycosylation patterns in the normal glycocalyx caused by cellular abnormalities may serve as highly sensitive and promising biomarkers. The present results strongly suggest importance of “human tissue glycome atlas” as well as “mouse tissue glycome atlas” for understanding the crucial and diversified roles of glycocalyx determined by the organ/tissue-characteristic *N*-glycosylation patterns and the discovery research for *N*-glycome-based disease-specific biomarkers and therapeutic targets^68^.

## Methods

### Protein extraction from mouse tissue

The frozen Slc:ddY mouse (6 weeks old) organs/tissues were obtained from Japan Bio Serum (Tokyo, Japan) except muscles, femurs, and serum which were purchased from Sankyo Labo Service Corporation, INK. (Tokyo, Japan). The frozen lungs derived in inflammatory inbred DBA/2Crslc mice and SLE model MRL-*lpr*/*lpr* mice were prepared by the method reported previously^69^. These animals were handled in accordance with the guidelines of the Institutional Animal Care and Use Committee of NIBIO (National Institute of Biomedical Innovation, Japan). The study protocol was approved by the Animal Ethics Review Committee of NIBIO (Protocol Number DS29-47). All experiments performed with mice were in accordance with the ARRIVE guidelines and regulations of this committee. In brief, after lungs collection from each strain, samples were placed in a separate screw-cup tube and immediately (within 20 s) snap-frozen. The organs were collected from five independent mice except for the thyroid and serum which were collected from the pooled samples. These frozen organs/tissues were lyophilized for 4 days and were ground with Automill (Tokken Inc., Kashiwa Japan). The powdery tissue samples (approximately 2–3 mm^3^) were sonicated in lysis buffer (0.1% SDS, 0.1% Triton X-100 in 100 mM ammonium hydrogen carbonate) to extract total proteins. The supernatants containing the proteins were stored at – 20 °C until use. All *N*-glycomics protocols using mouse tissues and serum samples were approved by the Institutional Animal Care and Use Committee of Hokkaido University and performed in accordance with ARRIVE guidelines and regulations of this committee (National University Corporation Hokkaido University Regulations on Animal Experimentation).

### Preparation of exosomes from mouse serum

The exosomes from frozen serum were purified by the basic protocol using ultracentrifugation^45^. Twenty mL of serum was centrifuged successively at increasing speed, i.e., 300 × *g* for 10 min*,* 2000 × *g* for 10 min, and 10,000 × *g* for 10 min at 4 °C three times to remove large dead cells and cellular debris. The final supernatant was ultracentrifuged in an Ultra-Clear centrifuge tube by using SW41 Ti swinging bucket rotor (Beckman Coulter, Brea, CA, USA) at 100,000 × *g* for 70 min at 4 °C to afford the pellet of the exosomes. The pellet was washed in a 50 μL of D-PBS (FUJIFILM Wako pure Chemical corporation, Osaka, Japan) to remove contaminating serum proteins and ultracentrifuged again at 100,000 × *g* for 70 min at 4 °C. Finally, the residual pellet was stored at 4 °C until use. The diameters of the exosomes were analyzed by the nanoparticle tracking analysis system, NanoSight (Quantum design Japan, Tokyo) (Fig. S1a).

### Western blotting

Total protein concentrations in 6 μg of exosome samples were determined by using BCA protein assay kit (Thermo Fisher Scientific, Waltham, MA, USA). As standard samples, we also employed 6 μg of mouse serum, lyophilized exosomes purified from human A549 cell culture supernatant (HansaBioMed Life Sciences, Tallinn, Estonia), and lyophilized exosomes purified from B16F10 mouse cell culture media (HansaBioMed Life Sciences). The exosome samples and standards were lysed in the buffer [0.25 M Tris–HCl (pH 6.8), 40% glycerol, 8% SDS, 0.08% bromophenol blue, and 800 mM DTT] and boiled for 5 min at 95 °C. These samples were subjected to electrophoresis using 5–20% polyacrylamide gel (c-PAGEL HR, ATTO Corporation, Tokyo, Japan) in Tris–glycine buffer at 5.5 mA for 40 min. Proteins were transferred onto PVDF membranes using Trans-Blot semi-dry electrophoretic transfer cell (BIO-RAD, Hercules, CA) at 20 V for 30 min, and the membranes were blocked with PVDF Blocking Reagent for Can Get Signal (TOYOBO, Osaka, Japan) for 1 h at room temperature. Membranes were incubated with anti-CD81 antibody (System Biosciences, Palo Alto, CA) at 1:1000 dilution and anti-CD9 antibody (System Biosciences) at 1:10,000 dilution at 4 °C overnight. After being washed with TBS-T buffer, the membranes were incubated with goat anti-rabbit HRP secondary antibody (System Biosciences) at room temperature for 1 h. After washing, the membranes were incubated with ECL Prime Western Blotting Detection Reagent (Cytiva, Tokyo, Japan) at room temperature for 5 min and visualized by using Luminescent image analyzer LAS-4000 EP mini (Fujifilm, Tokyo, Japan) (Fig. S1b).

### Solid-phase N-glycan enrichment, chemical modification, probing, and quantitation

After quantifying the total proteins by using BCA protein assay kit (Thermo Fisher Scientific, Waltham, MA, USA), the samples from organs/tissues/sera containing 100 μg of proteins and that from exosomes containing 10 μg of proteins were subjected to the digestion by 200 U of PNGase F (Peptide: *N*-glycosidase F from *Flavobacterium meningosepticum*, New England Biolabs, Ipswich, MA, USA) at 37 °C for 16 h. The crude *N*-glycans were purified and modified “on the solid-phase” based on a general protocol for glycoblotting^21^ (Fig. 1a) with a slight modification. Initially, 200 μL of BlotGlyco^Ⓡ^ H beads (Sumitomo Bakelite, Tokyo, Japan) in a 10 mg/mL suspension with water was placed into the wells of a MultiScreen^Ⓡ^ Solvinert filter plate (Merck Millipore Ltd., Burlington, Massachusetts, USA), and water was removed in vacuo. The samples were dried with a centrifugal evaporator and then suspended with 10 pmol (20 μL, 5.0 μmole/L in water) of non-natural *N*-glycan derivative [(Neu5Aca2, 6Galb1, 4GlcNAcb1, 2Man)_2_a1,3/6Manb1,4GlcNAc] as an internal standard (Tokyo Chemical Industry Co., Ltd, Tokyo, Japan). The solutions were applied into each well together with 180 μL of 2% acetic acid (AcOH) in acetonitrile (ACN). The plate was incubated at 80 °C for 45 min to capture the *N*-glycans in the samples onto the polymer beads through the hydrazone bonds. The plate was washed with 200 μL each of 2 M Guanidine-HCl in 16.6 mM ammonium hydrogen carbonate, MilliQ water, and 1% triethylamine in methanol twice each. Unreacted hydrazide groups of BlotGlyco^Ⓡ^ beads were capped by acetylation using 100 μL of 10% acetic anhydride in methanol at room temperature for 30 min. Each well was washed twice with 200 μL of 10 mM HCl aqueous solution, methanol, and dioxane. To protect the carboxyl group of the sialic acid residues, the residual BlotGlyco^Ⓡ^ beads were treated with 100 μL of 100 mM 1-methyl-3-*p*-tolytriazene (MTT) in dioxane at 60 °C for 90 min. After washing twice using 200 μL of dioxane, methanol, and MilliQ water, the beads were treated with 20 μL of 20 mM of the labeling reagent [aminooxy-Trp-Arg derivative (ao-WR), Sumitomo Bakelite, Tokyo, Japan] in MilliQ water and 180 μL of 2% AcOH in ACN at 90 °C for 45 min. The WR-labeled *N*-glycans in the residual mixture were eluted by 150 μL of MilliQ water and purified with GlycoWorks™ HILIC μElution Plate (Waters, Milford, Massachusetts, USA) according to the manufacturer’s description.

Purified WR-labeled *N*-glycans were mixed with an equal volume of matrix containing 10 mg/mL 2,5-dehydroxybenzoic acid (DHB) in 50% ACN on an MTP384 polished steel target plate (Bruker Daltonics, Billerica, Massachusetts, USA). And then the analytics were subjected to matrix-assisted laser desorption/ionization-time-of-flight mass spectrometry (MALDI-TOFMS) analysis by using Ultraflex III mass spectrometry (Bruker Daltonics, Billerica, MA). All the spectra were obtained using a reflection mode with an acceleration voltage of 25 kV, a reflector voltage of 26.3 kV, and a pulsed ion extraction of 90 ns in the positive ion mode. The spectra were the results of averaging of ~ 2000 laser shots. All peaks were picked by FlexAnalysis ver. 3.4 Software (Bruker Daltonics) using SNAP algorithm that fits isotopic patterns to the matching experimental data. The areas of isotopic patterns derived from *N*-glycans were quantitatively analyzed by comparing that for 100 pmol (10 pmol for exosomes) of an internal standard spiked in each status. The sugar compositions and proposed structures of *N*-glycans were assigned by Expasy GlycoMod Tool (https://web.expasy.org/glycomod/) using experimental masses. The predicted glycoforms of *N*-glycans identified in mouse 16 organs, tissues, serum, and serum-derived exosomes were summarized in Fig. S2, Tables S2 and S3.

### Statistical analysis

All the statistical analyses were performed with R 4.1.1 using the data of Tables S2 and S4. Figures 2 and 4 were obtained by analyzing the glycan levels in Table S2 with “gplots” package^70^ version 3.1.1 in R. The arguments for Figs. 3 and 5 were based on the glycotypes and sugar compositions described in Table S1. For the strain discrimination, *N*-glycans detected in the lungs of DBA and MRL were selected in concordance with those of Slc:ddY mouse (Table S4) and used for machine learning analysis with the dataset in Table S2. The dissimilarity analysis was performed by using Canberra distances^71^ of each *N*-glycosylation pattern in Table S2. In the hierarchical cluster dendrogram (Fig. 6), the distances between clusters were calculated based on Ward’s method with “stats” package in R 4.1.1. For the machine learning analysis of mouse tissue *N*-glycome (Fig. 7a), we employed the *N*-glycan levels described in Table S2. In the case of the strain discrimination (Fig. 7b), the *N*-glycan levels observed in the lungs of DBA and MRL were selected in concordance with those of Slc:ddY mouse (Table S4) and used for machine learning analysis with the dataset in Table S2. The test data were randomly selected one of five replicates by stratified sampling, while the other four of five samples were used as learning data. Data partitions were created for each prediction using the “createDataPartition” function in “caret” package^72^. Therefore, the test data was a dataset with one randomly selected from each organ on each trial. The R packages used for decision tree, random forest, SVM, and neural network were “rpart”^73^ version 4.1–15, “randomForest”^74^ version 4.6–14, “kernlab”^75^ version 0.9–29, and “nnet”^76^ version 7.3–16, respectively. All the best parameters for four algorithms used were tuned by means of “caret” package version 6.0–88 and the best values were used for the following analysis. The F1-scores for discriminating among organs or strains were calculated by repeating more than1000 times prediction (Table S5).

## Supplementary Information

Supplementary Information.

## Data Availability

The datasets generated during the current study are available in the Supplementary Information and will be deposited in a suited database as soon as possible.

## Abbreviations

Fuc: Fucose
Gal: Galactose
GalNAc: *N*-acetylgalactosamine
Glc: Glucose
GlcNAc: *N*-acetylglucosamine
Hex: Hexose
HexNAc: *N*-acetylhexosamine
Man: Mannose
Neu5Ac: *N*-acetylneuraminic acid
Neu5Gc: *N*-glycolylneuraminic acid

## References

[1] Varki A (2017). Biological roles of glycans. Glycobiology.

[2] Paszek MJ (2014). The cancer glycocalyx mechanically primes integrin-mediated growth and survival. Nature.

[3] Kuo JC-H (2018). Physical biology of the cancer cell glycocalyx. Nat. Phys..

[4] Varki A, Cummings RD, Esko JD, Freeze HH, Stanley P, Bertozzi CR, Hart GW, Etzler ME (2008). Essentials of Glycobiology.

[5] Dennis JW, Nabi IR, Demetriou M (2009). Metabolism, cell surface organization, and disease. Cell.

[6] Rabinovich GA, Croci DO (2012). Regulatory circuits mediated by lectin-glycan interactions in autoimmunity and cancer. Immunity.

[7] Peixoto A, Relvas-Santos M, Azevedo R, Lara Santos L, Ferreira JA (2019). Protein glycosylation and tumor microenvironment alterations driving cancer hallmarks. Front. Oncol..

[8] Pathan M (2019). Vesiclepedia 2019: A compendium of RNA, proteins, lipids and metabolites in extracellular vesicles. Nucleic Acids Res..

[9] Mulcahy LA, Pink RC, Carter DRF (2014). Routes and mechanisms of extracellular vesicle uptake. J. Extracell. Ves..

[10] Kurywchak P, Tavormina J, Kalluri R (2018). The emerging roles of exosomes in the modulation of immune responses in cancer. Genome Med..

[11] Zomer A (2015). In vivo imaging reveals extracellular vesicle-mediated phenocopying of metastatic behavior. Cell.

[12] Sung BH, Ketova T, Hoshino D, Zijlstra A, Weaver AM (2015). Directional cell movement through tissues is controlled by exosome secretion. Nat. Commun..

[13] Becker A, Thakur BK, Weiss JM, Kim HS, Peinado H, Lyden D (2016). Extracellular vesicles in cancer: Cell-to-cell mediators of metastasis. Cancer Cell.

[14] Colombo M, Raposo G, Théry C (2014). Biogenesis, secretion, and intercellular interactions of exosomes and other extracellular vesicles. Annu. Rev. Cell Dev. Biol..

[15] Hoshino A (2015). Tumour exosome integrins determine organotropic metastasis. Nature.

[16] Martins ÁM, Ramos CC, Freitas D, Reis CA (2021). Glycosylation of cancer extracellular vesicles: Capture strategies, functional roles and potential clinical applications. Cells.

[17] Koide R, Hirane N, Kambe D, Yokoi Y, Otaki M, Nishimura S-I (2022). Antiadhesive nanosome elicits role of glycocalyx of tumor cell-derived exosomes in the organotropic cancer metastasis. Biomaterials.

[18] Amano M (2010). Threshold in stage-specific embryonic glycotypes uncovered by a full portrait of dynamic *N*-glycan expression during cell differentiation. Mol. Cell. Proteomics.

[19] Garcia-Vallejo JJ, van Kooyk Y (2013). The physiological role of DC-SIGN: A tale of mice and men. Trends Immunol..

[20] Davies LC, Jenkins SJ, Allen JE, Taylor PR (2013). Tissue-resident macrophages. Nat. Immunol..

[21] Macauley MS, Crocker PR, Paulson JC (2014). Siglec-mediated regulation of immune cell function in disease. Nat. Rev. Immunol..

[22] Nishimura S-I (2011). Toward automated glycan analysis. Adv. Carbohydr. Chem. Biochem..

[23] Li Q, Xie Y, Wong M, Barboza M, Lebrilla CB (2020). Comprehensive structural glycomic characterization of the glycocalyxes of cells and tissues. Nat. Protocol..

[24] Polasky D, Yu F, Teo GC, Nesvizhskii AI (2020). Fast and comprehensive *N*- and *O*-glycoproteomics analysis with MSFragger-Glyco. Nat. Methods.

[25] Peng W, Reyes CDG, Gautam S, Yu A, Cho BG, Goli M, Donohoo K, Mondello S, Kobeissy F, Mechref Y (2022). MS-based glycomics and glycoproteomics methods enabling isomeric characterization. Mass Spec. Rev..

[26] Nishimura S-I (2005). High-throughput protein glycomics: Combined use of chemoselective glycoblotting and MALDI-TOF/TOF mass spectrometry. Angew. Chem. Int. Ed..

[27] Furukawa J, Shinohara Y, Kuramoto H, Miura Y, Shimaoka H, Kurogochi M, Nakano M, Nishimura S-I (2008). Comprehensive approach to structural and functional glycomics based on chemoselective glycoblotting and sequential tag conversion. Anal. Chem..

[28] Miura Y (2008). BlotGlycoABCTM: An integrated glycoblotting technique for rapid and large-scale clinical glycome. Mol. Cell. Proteom..

[29] Kamiyama T (2013). Identification of novel serum biomarkers of hepatocellular carcinoma using glycomic analysis. Hepatology.

[30] Nouso K (2013). Clinical utility of high-throughput glycome analysis in patients with pancreatic cancer. J. Gastroenterol..

[31] Hatakeyama S (2014). Serum *N*-glycan alteration associated with renal cell carcinoma detected by high-throughput glycan analysis. J. Urol..

[32] Gebrehiwot Abrha G, Seifu Melka D, Mamo Kassaye Y, Gemechu T, Lako W, Hinou H, Nishimura S-I (2019). Exploring serum and Immunoglobulin G *N*-glycome as diagnostic biomarkers for early detection of breast cancer in Ethiopian women. BMC Cancer.

[33] Matsumoto T (2019). Serum *N*-glycan profiling is a potential biomarker for castration-resistant prostate cancer. Sci. Rep..

[34] Miyahara K (2013). Serum glycan markers for evaluation of disease activity and prediction of clinical course in patients with ulcerative colitis. PLoS ONE.

[35] Inafuku S, Noda K, Amano M, Ohashi T, Yoshizawa C, Saito W, Murata M, Kanda A, Nishimura S-I, Ishida S (2015). Alteration of *N*-glycan profiles in diabetic retinopathy. Invest. Ophthalmol. Vis. Sci..

[36] Inafuku S, Noda K, Amano M, Nishimura S-I, Ishida S (2016). Increase of sialylated *N*-glycansin eyes in with neovascular glaucoma secondary to proliferative diabetic retinopathy. Curr. Eye Res..

[37] Ishihara T (2014). Discovery of novel differentiation markers in the early stage of chondrogenesis by glycoform-focused reverse proteomics and genomics. BBA-GenSubjects.

[38] Nishimura S-I, Ishihara T, Iwasaki N, Preedy VR (2016). Differentiation biomarkers of osteoarthritis determined by glycoblotting. Biomarkers in Bone Disease, Biomarkers in Disease: Methods, Discoveries and Applications.

[39] Gizaw ST, Ohashi T, Tanaka M, Hinou H, Nishimura S-I (2016). Glycoblotting method allows for rapid and efficient glycome profiling of human Alzheimer’s disease brain, serum and cerebrospinal fluid towards potential biomarker discovery. BBA-Gen Subjects.

[40] Terashima M, Amano M, Onodera T, Nishimura S-I, Iwasaki N (2014). Quantitative glycomics monitoring of induced pluripotent- and embryonic stem cells during neuronal differentiation. Stem Cell Res..

[41] Gizaw ST, Koda T, Amano M, Kamimura K, Ohashi T, Hinou H, Nishimura S-I (2015). A comprehensive glycome profiling of Huntington's disease transgenic mice. BBA-Gen Subjects.

[42] Kanapin A (2003). Mouse proteome analysis. Genome Res..

[43] Lloyd KCK (2020). The deep genome project. Genome Biol..

[44] Reel PS, Reel S, Pearson E, Trucco E, Jefferson E (2021). Using machine learning approaches for multi-omics data analysis: A review. Biotech. Adv..

[45] Théry C, Clayton A, Amigorena S, Raposo G (2006). Isolation and characterization of exosomes from cell culture supernatants. Curr. Prot. Cell Biol..

[46] Mathieu M, Martin-Jaular L, Lavieu G, Théry C (2019). Specificities of secretion and uptake of exosomes and other extracellular vesicles for cell-to-cell communication. Nat. Cell Biol..

[47] Nishikaze T, Tsumoto H, Sekiya S, Iwamoto S, Miura Y, Tanaka K (2017). Differentiation of sialyl linkage isomers by one-pot sialic acid derivatization for mass spectrometry-based glycan profiling. Anal. Chem..

[48] Yang S, Jakowska E, Kosikova M, Xie H, Cipollo J (2017). Solid-phase chemical modification for sialic acid linkage analysis: Application to glycoproteins of host cells used in influenza virus propagation. Anal. Chem..

[49] Amano M, Hashimoto R, Nishimura S-I (2012). Effects of single genetic damage in carbohydrate-recognizing proteins on mouse serum *N*-glycan profile revealed by a simple glycotyping analysis. ChemBioChem.

[50] Hirose K, Amano M, Hashimoto R, Lee YC, Nishimura S-I (2011). Insight into glycan diversity and evolutional lineage based on comparative avio-*N*-glycomics and sialic acid analysis of 88 egg whites of Galloanserae. Biochemistry.

[51] Misra BB, Langefeld C, Oliver M, Cox LA (2019). Integrated omics: Tools, advances and future approaches. J. Mol. Endocrinol..

[52] Kim M, Tagkopoulos I (2018). Data integration and predictive modeling methods for multi-omics datasets. Mol. Omics.

[53] Meng C, Zeleznik OA, Thallinger GG, Kuster B, Gholami AM, Culhane A (2016). Dimension reduction techniques for the integrative analysis of multi-omics data. Brief. Bioinform..

[54] Frank EA (2017). Generic susceptibility to toxicologic lung response among inbred mouse strains following exposure to carbon nanotubes and profiling of underlying gene networks. Toxicol. Appl. Pharmacol..

[55] Pompilio A (2014). Stenotrophomonas maltophilia virulence and specific variations in trace elements during acute lung infection: Implication in cystic fibrosis. PLoS ONE.

[56] Sugimoto K (2017). Autoimmune disease mouse model exhibits pulmonary arterial hypertension. PLoS ONE.

[57] Liu Z (2016). Tumor necrosis factor-like weak inducer of apoptosis accelerates the progression of renal fibrosis in lupus nephritis by activating SMAD and p38 MAPK in TGF-b1 signaling pathway. Mediators Inflamm..

[58] Andrews BYBS (1978). Spontaneous murine lupus-like syndromes. Clinical and immunopathological manifestations in several strains. J. Exp. Med..

[59] Yamakawa N (2018). Systems glycomics of adult zebrafish identifies organ-specific sialylation and glycosylation patterns. Nat. Commun..

[60] Nagai-Okatani C (2019). LM-Glycome atlas ver 1.0: A novel visualization tool for lectin microarray-based glycomic profiles of mouse tissue sections. Molecules.

[61] Reiding KR (2019). High-throughput serum *N*-glycomics: Method comparison and application to study rheumatoid arthritis and pregnancy-associated changes. Mol. Cell. Proteomics.

[62] Ji IJ (2015). Spatially-resolved exploration of the mouse brain glycome by tissue glyco-capture (TGC) and nano-LC/MS. Anal. Chem..

[63] Mohammed Y, Bhowmick P, Michaud SA, Sickmann A, Borchers CH (2021). Mouse quantitative proteomics knowledgebase: Reference protein concentration ranges in 20 mouse tissues using 5000 quantitative proteomics assays. Bioinform..

[64] Kurogochi M (2010). Sialic acid-focused quantitative mouse serum glycoproteomics by multiple reaction monitoring assay. Mol. Cell. Proteomics.

[65] Arabyan N (2016). *Salmonella* degrades the host glycocalyx leading to altered infection and glycan remodeling. Sci. Rep..

[66] Yeo TW (2019). Glycocalyx breakdown is associated with severe disease and fatal outcome in *Plasmodium falciparum* malaria. Clin. Infec. Diseases.

[67] Möckl L (2020). The emerging role of the mammalian glycocalyx in functional membrane organization and immune system regulation. Front. Cell Dev. Biol..

[68] Lauc G, Pezer M, Rudan I, Campbell H (2016). Mechanisms of disease: The human *N*-glycome. BBA-GenSubjects.

[69] Abe Y (2020). Comprehensive characterization of the phosphoproteome of gastric cancer from endoscopic biopsy specimens. Theranostics.

[70] Warnes, G. R. *et al*. gplots: Various R programming tools for plotting data. *R Package version 3.1.1* (2020).

[71] Lance GN, Williams WT (1966). Computer programs for hierarchical polythetic classification (‘Similarity Analyses’). Comput. J..

[72] Kuhn M (2008). Building predictive models in R using the caret package. J. Stat. Softw..

[73] Therneau, T., Atkinson, B., Ripley, B. rpart: Recursive partitioning and regression trees. (2022).

[74] Liaw A, Wiener M (2002). Classification and regression by randomForest. R News.

[75] Li YL, Zhang YP (2011). kernlab-An S4 package for kernel methods in R. Adv. Mater. Res..

[76] Venables W, Ripley B (2002). Modern Applied Statistics with S.

