# Supplementary material for: Mouse tissue glycome atlas 2022 highlights inter-organ variation in major N-glycan profiles
Source: Sci Rep. 2022 Oct 24;12:17804. doi: 10.1038/s41598-022-21758-4 (PMC9592591; doi:10.1038/s41598-022-21758-4)
Supplement: Supplementary file 1 — Supplementary Information. [file 41598_2022_21758_MOESM1_ESM.docx]

Supplementary Information

**Table S1**. List of the estimated *N*-glycans and the sugar compositions of mouse 16 organs, serum, and serum exosomes.

| Peak No. | Predicted m/z | Abbreviation | Glycan composition | Type | HexNAc | End | Sialic | Fucose |
| --- | --- | --- | --- | --- | --- | --- | --- | --- |
| 1 | 1340.55 | H3N2 | (Hex)3 (HexNAc)2 | Pauciman | 0 | Man | 0 | 0 |
| 2 | 1486.60 | H3N2D1 | (Hex)3 (HexNAc)2 (Deoxyhexose)1 | Pauciman | 0 | Man | 0 | 1 |
| 3 | 1502.60 | H4N2 | (Hex)4 (HexNAc)2 | Pauciman | 0 | Man | 0 | 0 |
| 4 | 1543.63 | H3N3 | (HexNAc)1 + (Man)3(GlcNAc)2 | complex | 0 | GlcNAc | 0 | 0 |
| 5 | 1648.66 | H4N2D1 | (Hex)4 (HexNAc)2 (Deoxyhexose)1 | Pauciman | 0 | Man | 0 | 1 |
| 6 | 1664.65 | H5N2 | (Hex)2 + (Man)3(GlcNAc)2 | high_man | 0 | Man | 0 | 0 |
| 7 | 1689.68 | H3N3D1 | (HexNAc)1 (Deoxyhexose)1 + (Man)3(GlcNAc)2 | complex | 1 | GlcNAc | 0 | 1 |
| 8 | 1705.68 | H4N3 | (Hex)1 (HexNAc)1 + (Man)3(GlcNAc)2 | complex | 1 | Gal | 0 | 0 |
| 9 | 1746.70 | H3N4 | (HexNAc)2 + (Man)3(GlcNAc)2 | complex | 2 | GlcNAc | 0 | 0 |
| 10 | 1810.71 | H5N2D1 | (Hex)2 (Deoxyhexose)1 + (Man)3(GlcNAc)2 | high_man | 0 | Man | 0 | 1 |
| 11 | 1826.71 | H6N2 | (Hex)3 + (Man)3(GlcNAc)2 | high_man | 0 | Man | 0 | 0 |
| 12 | 1851.74 | H4N3D1 | (Hex)1 (HexNAc)1 (Deoxyhexose)1 + (Man)3(GlcNAc)2 | complex | 1 | Gal | 0 | 1 |
| 13 | 1867.73 | H5N3 | (Hex)2 (HexNAc)1 + (Man)3(GlcNAc)2 | hybrid | 1 | Gal | 0 | 0 |
| 14 | 1892.76 | H3N4D1 | (HexNAc)2 (Deoxyhexose)1 + (Man)3(GlcNAc)2 | complex | 2 | GlcNAc | 0 | 1 |
| 15 | 1908.76 | H4N4 | (Hex)1 (HexNAc)2 + (Man)3(GlcNAc)2 | complex | 2 | Gal | 0 | 0 |
| 16 | 1949.78 | H3N5 | (HexNAc)3 + (Man)3(GlcNAc)2 | complex | 3 | GlcNAc | 0 | 0 |
| 17 | 1988.76 | H7N2 | (Hex)4 + (Man)3(GlcNAc)2 | high_man | 0 | Man | 0 | 0 |
| 18 | 1997.79 | H4N3D2 | (Hex)1 (HexNAc)1 (Deoxyhexose)2 + (Man)3(GlcNAc)2 | complex | 1 | Gal | 0 | 2 |
| 19 | 2010.79 | H4N3A1 | (Hex)1 (HexNAc)1 (NeuAc)1 + (Man)3(GlcNAc)2 | complex | 1 | Sia | 1 | 0 |
|  |  |  | (HexNAc)1 (Deoxyhexose)1 (NeuGc)1 + (Man)3(GlcNAc)2 |  |  |  |  |  |
| 20 | 2013.79 | H5N3D1 | (Hex)2 (HexNAc)1 (Deoxyhexose)1 + (Man)3(GlcNAc)2 | hybrid | 1 | Hybrid | 0 | 1 |
| 21 | 2026.79 | H4N3G1 | (Hex)1 (HexNAc)1 (NeuGc)1 + (Man)3(GlcNAc)2 | complex | 1 | Sia | 1 | 0 |
| 22 | 2029.78 | H6N3 | (Hex)3 (HexNAc)1 + (Man)3(GlcNAc)2 | hybrid | 1 | Gal | 0 | 0 |
| 23 | 2054.82 | H4N4D1 | (Hex)1 (HexNAc)2 (Deoxyhexose)1 + (Man)3(GlcNAc)2 | complex | 2 | Gal | 0 | 1 |
| 24 | 2070.81 | H5N4 | (Hex)2 (HexNAc)2 + (Man)3(GlcNAc)2 | complex | 2 | Gal | 0 | 0 |
| 25 | 2095.84 | H3N5D1 | (HexNAc)3 (Deoxyhexose)1 + (Man)3(GlcNAc)2 | complex | 3 | GlcNAc | 0 | 1 |
| 26 | 2111.84 | H4N5 | (Hex)1 (HexNAc)3 + (Man)3(GlcNAc)2 | complex | 3 | Gal | 0 | 0 |
| 27 | 2150.81 | H8N2 | (Hex)5 + (Man)3(GlcNAc)2 | high_man | 0 | Man | 0 | 0 |
| 28 | 2156.85 | H4N3D1A1 | (Hex)1 (HexNAc)1 (Deoxyhexose)1 (NeuAc)1 + (Man)3(GlcNAc)2 | complex | 1 | Sia | 1 | 1 |
|  |  |  | (HexNAc)1 (Deoxyhexose)2 (NeuGc)1 + (Man)3(GlcNAc)2 |  |  |  |  |  |
| 29 | 2172.84 | H5N3A1 | (Hex)2 (HexNAc)1 (NeuAc)1 + (Man)3(GlcNAc)2 | Hybrid | 1 | Sia | 1 | 0 |
|  |  |  | (Hex)1 (HexNAc)1 (Deoxyhexose)1 (NeuGc)1 + (Man)3(GlcNAc)2 |  |  |  |  |  |
| 30 | 2175.84 | H6N3D1 | (Hex)3 (HexNAc)1 (Deoxyhexose)1 + (Man)3(GlcNAc)2 | hybrid | 1 | Gal | 0 | 1 |
| 31 | 2185.84 | H3N3G2 | (HexNAc)1 (NeuGc)2 + (Man)3(GlcNAc)2 | complex | 1 | Sia | 2 | 0 |
| 32 | 2188.84 | H5N3G1 | (Hex)2 (HexNAc)1 (NeuGc)1 + (Man)3(GlcNAc)2 | hybrid | 1 | Sia | 1 | 0 |
| 33 | 2200.87 | H4N4D2 | (Hex)1 (HexNAc)2 (Deoxyhexose)2 + (Man)3(GlcNAc)2 | complex | 2 | Gal | 0 | 2 |
| 34 | 2213.87 | H4N4A1 | (Hex)1 (HexNAc)2 (NeuAc)1 + (Man)3(GlcNAc)2 | complex | 2 | Sia | 1 | 0 |
|  |  |  | (HexNAc)2 (Deoxyhexose)1 (NeuGc)1 + (Man)3(GlcNAc)2 |  |  |  |  |  |
| 35 | 2216.87 | H5N4D1 | (Hex)2 (HexNAc)2 (Deoxyhexose)1 + (Man)3(GlcNAc)2 | complex | 2 | Gal | 0 | 1 |
| 36 | 2229.86 | H4N4G1 | (Hex)1 (HexNAc)2 (NeuGc)1 + (Man)3(GlcNAc)2 | complex | 2 | Sia | 1 | 0 |
| 37 | 2232.86 | H6N4 | (Hex)3 (HexNAc)2 + (Man)3(GlcNAc)2 | complex | 2 | Gal | 0 | 0 |
| 38 | 2241.90 | H3N5D2 | (HexNAc)3 (Deoxyhexose)2 + (Man)3(GlcNAc)2 | complex | 3 | GlcNAc | 0 | 2 |
| 39 | 2257.90 | H4N5D1 | (Hex)1 (HexNAc)3 (Deoxyhexose)1 + (Man)3(GlcNAc)2 | complex | 3 | Gal | 0 | 1 |
| 40 | 2273.89 | H5N5 | (Hex)2 (HexNAc)3 + (Man)3(GlcNAc)2 | complex | 3 | Gal | 0 | 0 |
| 41 | 2298.92 | H3N6D1 | (HexNAc)4 (Deoxyhexose)1 + (Man)3(GlcNAc)2 | complex | 2 | GalNAc | 0 | 1 |
| 42 | 2312.86 | H9N2 | (Hex)6 + (Man)3(GlcNAc)2 | high_man | 0 | Man | 0 | 0 |
| 43 | 2318.90 | H5N3D1A1 | (Hex)2 (HexNAc)1 (Deoxyhexose)1 (NeuAc)1 + (Man)3(GlcNAc)2 | hybrid | 1 | Sia | 1 | 1 |
|  |  |  | (Hex)1 (HexNAc)1 (Deoxyhexose)2 (NeuGc)1 + (Man)3(GlcNAc)2 |  |  |  |  |  |
| 44 | 2334.90 | H6N3A1 | (Hex)3 (HexNAc)1 (NeuAc)1 + (Man)3(GlcNAc)2 | hybrid | 1 | Sia | 1 | 0 |
|  |  |  | (Hex)2 (HexNAc)1 (Deoxyhexose)1 (NeuGc)1 + (Man)3(GlcNAc)2 |  |  |  |  |  |
| 45 | 2350.89 | H6N3G1 | (Hex)3 (HexNAc)1 (NeuGc)1 + (Man)3(GlcNAc)2 | hybrid | 1 | Sia | 1 | 0 |
| 46 | 2359.93 | H4N4D1A1 | (Hex)1 (HexNAc)2 (Deoxyhexose)1 (NeuAc)1 + (Man)3(GlcNAc)2 | complex | 2 | Sia | 1 | 1 |
|  |  |  | (HexNAc)2 (Deoxyhexose)2 (NeuGc)1 + (Man)3(GlcNAc)2 |  |  |  |  |  |
| 47 | 2362.93 | H5N4D2 | (Hex)2 (HexNAc)2 (Deoxyhexose)2 + (Man)3(GlcNAc)2 | complex | 2 | Gal | 0 | 2 |
| 48 | 2375.92 | H5N4A1 | (Hex)2 (HexNAc)2 (NeuAc)1 + (Man)3(GlcNAc)2 | complex | 2 | Sia | 1 | 0 |
|  |  |  | (Hex)1 (HexNAc)2 (Deoxyhexose)1 (NeuGc)1 + (Man)3(GlcNAc)2 |  |  |  |  |  |
| 49 | 2378.92 | H6N4D1 | (Hex)3 (HexNAc)2 (Deoxyhexose)1 + (Man)3(GlcNAc)2 | complex | 2 | Gal | 0 | 1 |
| 50 | 2391.92 | H5N4G1 | (Hex)2 (HexNAc)2 (NeuGc)1 + (Man)3(GlcNAc)2 | complex | 2 | Sia | 1 | 0 |
| 51 | 2403.95 | H4N5D2 | (Hex)1 (HexNAc)3 (Deoxyhexose)2 + (Man)3(GlcNAc)2 | complex | 3 | Gal | 0 | 2 |
| 52 | 2416.95 | H4N5A1 | (Hex)1 (HexNAc)3 (NeuAc)1 + (Man)3(GlcNAc)2 | complex | 3 | Sia | 1 | 0 |
|  |  |  | (HexNAc)3 (Deoxyhexose)1 (NeuGc)1 + (Man)3(GlcNAc)2 |  |  |  |  |  |
| 53 | 2419.95 | H5N5D1 | (Hex)2 (HexNAc)3 (Deoxyhexose)1 + (Man)3(GlcNAc)2 | complex | 3 | Gal | 0 | 1 |
| 54 | 2435.94 | H6N5 | (Hex)3 (HexNAc)3 + (Man)3(GlcNAc)2 | complex | 3 | Gal | 0 | 0 |
| 55 | 2444.98 | H3N6D2 | (HexNAc)4 (Deoxyhexose)2 + (Man)3(GlcNAc)2 | complex | 2 | GalNAc | 0 | 2 |
| 56 | 2445.96 | H3N3D2A2 | (HexNAc)1 (Deoxyhexose)2 (NeuAc)2 + (Man)3(GlcNAc)2 | complex | 1 | Sia | 2 | 2 |
| 57 | 2460.98 | H4N6D1 | (Hex)1 (HexNAc)4 (Deoxyhexose)1 + (Man)3(GlcNAc)2 | complex | 4 | Gal | 0 | 1 |
| 58 | 2474.92 | H10N2 | (Hex)7 + (Man)3(GlcNAc)2 | high_man | 0 | Glc | 0 | 0 |
|  |  |  | (HexNAc)1 (NeuAc)2 (NeuGc)1 + (Man)3(GlcNAc)2 |  |  |  |  |  |
| 59 | 2477.95 | I. S. | InternalStandard (Hex)5 (HexNAc)3 (NeuAc)2 | IS | IS | IS | IS | IS |
| 60 | 2489.99 | H3N4D3A1 | (HexNAc)2 (Deoxyhexose)3 (NeuAc)1 + (Man)3(GlcNAc)2 | complex | 2 | Sia | 1 | 3 |
| 61 | 2499.95 | H8N3D1 | (Hex)5 (HexNAc)1 (Deoxyhexose)1 + (Man)3(GlcNAc)2 | hybrid | 1 | Gal | 0 | 1 |
| 62 | 2502.00 | H3N7D1 | (HexNAc)5 (Deoxyhexose)1 + (Man)3(GlcNAc)2 | complex | 5 | GlcNAc | 0 | 1 |
| 63 | 2505.99 | H4N4D2A1 | (Hex)1 (HexNAc)2 (Deoxyhexose)2 (NeuAc)1 + (Man)3(GlcNAc)2 | complex | 2 | Sia | 1 | 2 |
|  |  |  | (HexNAc)2 (Deoxyhexose)3 (NeuGc)1 + (Man)3(GlcNAc)2 |  |  |  |  |  |
| 64 | 2508.99 | H5N4D3 | (Hex)2 (HexNAc)2 (Deoxyhexose)3 + (Man)3(GlcNAc)2 | complex | 2 | Gal | 0 | 3 |
| 65 | 2518.00 | H4N7 | (Hex)1 (HexNAc)5 + (Man)3(GlcNAc)2 | complex | 5 | Gal | 0 | 0 |
|  |  |  | (HexNAc)2 (Deoxyhexose)1 (NeuAc)1 (NeuGc)1 + (Man)3(GlcNAc)2 |  |  |  |  |  |
| 66 | 2518.98 | H4N4A2 | (Hex)1 (HexNAc)2 (NeuAc)2 + (Man)3(GlcNAc)2 | complex | 2 | Sia | 2 | 0 |
| 67 | 2521.98 | H5N4D1A1 | (Hex)2 (HexNAc)2 (Deoxyhexose)1 (NeuAc)1 + (Man)3(GlcNAc)2 | complex | 2 | Sia | 1 | 1 |
|  |  |  | (Hex)1 (HexNAc)2 (Deoxyhexose)2 (NeuGc)1 + (Man)3(GlcNAc)2 |  |  |  |  |  |
| 68 | 2524.98 | H6N4D2 | (Hex)3 (HexNAc)2 (Deoxyhexose)2 + (Man)3(GlcNAc)2 | hybrid | 2 | Gal | 0 | 2 |
| 69 | 2534.98 | H3N4D1G2 | (HexNAc)2 (Deoxyhexose)1 (NeuGc)2 + (Man)3(GlcNAc)2 | complex | 2 | Sia | 2 | 1 |
|  |  |  | (Hex)1 (HexNAc)2 (NeuAc)1 (NeuGc)1 + (Man)3(GlcNAc)2 |  |  |  |  |  |
| 70 | 2537.97 | H5N4D1G1 | (Hex)2 (HexNAc)2 (Deoxyhexose)1 (NeuGc)1 + (Man)3(GlcNAc)2 | complex | 2 | Sia | 1 | 1 |
|  |  |  | (Hex)3 (HexNAc)2 (NeuAc)1 + (Man)3(GlcNAc)2 |  |  |  |  |  |
| 71 | 2540.97 | H7N4D1 | (Hex)4 (HexNAc)2 (Deoxyhexose)1 + (Man)3(GlcNAc)2 | complex | 2 | Gal | 0 | 1 |
| 72 | 2550.01 | H4N5D3 | (Hex)1 (HexNAc)3 (Deoxyhexose)3 + (Man)3(GlcNAc)2 | complex | 3 | Gal | 0 | 3 |
| 73 | 2553.97 | H6N4G1 | (Hex)3 (HexNAc)2 (NeuGc)1 + (Man)3(GlcNAc)2 | complex | 2 | Sia | 1 | 0 |
| 74 | 2563.01 | H4N5D1A1 | (Hex)1 (HexNAc)3 (Deoxyhexose)1 (NeuAc)1 + (Man)3(GlcNAc)2 | complex | 3 | Sia | 1 | 1 |
|  |  |  | (HexNAc)3 (Deoxyhexose)2 (NeuGc)1 + (Man)3(GlcNAc)2 |  |  |  |  |  |
| 75 | 2566.01 | H5N5D2 | (Hex)2 (HexNAc)3 (Deoxyhexose)2 + (Man)3(GlcNAc)2 | complex | 3 | Gal | 0 | 2 |
| 76 | 2579.00 | H5N5A1 | (Hex)2 (HexNAc)3 (NeuAc)1 + (Man)3(GlcNAc)2 | complex | 3 | Sia | 1 | 0 |
|  |  |  | (Hex)1 (HexNAc)3 (Deoxyhexose)1 (NeuGc)1 + (Man)3(GlcNAc)2 |  |  |  |  |  |
| 77 | 2582.00 | H6N5D1 | (Hex)3 (HexNAc)3 (Deoxyhexose)1 + (Man)3(GlcNAc)2 | complex | 3 | Gal | 0 | 1 |
| 78 | 2588.99 | H8N2D3 | (Hex)5 (Deoxyhexose)3 + (Man)3(GlcNAc)2 | high_man | 0 | Man | 0 | 3 |
| 79 | 2591.04 | H3N6D3 | (HexNAc)4 (Deoxyhexose)3 + (Man)3(GlcNAc)2 | complex | 4 | GlcNAc | 0 | 3 |
| 80 | 2607.03 | H4N6D2 | (Hex)1 (HexNAc)4 (Deoxyhexose)2 + (Man)3(GlcNAc)2 | complex | 3 | GalNAc | 0 | 2 |
| 81 | 2623.03 | H5N6D1 | (Hex)2 (HexNAc)4 (Deoxyhexose)1 + (Man)3(GlcNAc)2 | complex | 4 | Gal | 0 | 1 |
| 82 | 2671.04 | H6N4D3 | (Hex)3 (HexNAc)2 (Deoxyhexose)3 + (Man)3(GlcNAc)2 | complex | 2 | Gal | 0 | 3 |
| 83 | 2680.05 | H5N7 | (Hex)2 (HexNAc)5 + (Man)3(GlcNAc)2 | complex | 5 | Gal | 0 | 0 |
| 84 | 2681.03 | H5N4A2 | (Hex)2 (HexNAc)2 (NeuAc)2 + (Man)3(GlcNAc)2 | complex | 2 | Sia | 2 | 0 |
|  |  |  | (Hex)1 (HexNAc)2 (Deoxyhexose)1 (NeuAc)1 (NeuGc)1 + (Man)3(GlcNAc)2 |  |  |  |  |  |
|  |  |  | (HexNAc)2 (Deoxyhexose)2 (NeuGc)2 + (Man)3(GlcNAc)2 |  |  |  |  |  |
| 85 | 2684.03 | H6N4D1A1 | (Hex)3 (HexNAc)2 (Deoxyhexose)1 (NeuAc)1 + (Man)3(GlcNAc)2 | complex | 2 | Sia | 1 | 1 |
|  |  |  | (Hex)2 (HexNAc)2 (Deoxyhexose)2 (NeuGc)1 + (Man)3(GlcNAc)2 |  |  |  |  |  |
| 86 | 2697.03 | H5N4A1G1 | (Hex)2 (HexNAc)2 (NeuAc)1 (NeuGc)1 + (Man)3(GlcNAc)2 | complex | 2 | Sia | 2 | 0 |
|  |  |  | (Hex)1 (HexNAc)2 (Deoxyhexose)1 (NeuGc)2 + (Man)3(GlcNAc)2 |  |  |  |  |  |
| 87 | 2700.03 | H7N4A1 | (Hex)4 (HexNAc)2 (NeuAc)1 + (Man)3(GlcNAc)2 | Hybrid | 2 | Sia | 1 | 0 |
|  |  |  | (Hex)3 (HexNAc)2 (Deoxyhexose)1 (NeuGc)1 + (Man)3(GlcNAc)2 |  |  |  |  |  |
| 88 | 2712.06 | H5N5D3 | (Hex)2 (HexNAc)3 (Deoxyhexose)3 + (Man)3(GlcNAc)2 | complex | 3 | Gal | 0 | 3 |
| 89 | 2713.02 | H5N4G2 | (Hex)2 (HexNAc)2 (NeuGc)2 + (Man)3(GlcNAc)2 | complex | 2 | Sia | 2 | 0 |
| 90 | 2725.06 | H5N5D1A1 | (Hex)2 (HexNAc)3 (Deoxyhexose)1 (NeuAc)1 + (Man)3(GlcNAc)2 | complex | 3 | Sia | 1 | 1 |
|  |  |  | (Hex)1 (HexNAc)3 (Deoxyhexose)2 (NeuGc)1 + (Man)3(GlcNAc)2 |  |  |  |  |  |
| 91 | 2741.05 | H6N5A1 | (Hex)3 (HexNAc)3 (NeuAc)1 + (Man)3(GlcNAc)2 | complex | 3 | Sia | 1 | 0 |
|  |  |  | (Hex)2 (HexNAc)3 (Deoxyhexose)1 (NeuGc)1 + (Man)3(GlcNAc)2 |  |  |  |  |  |
| 92 | 2766.09 | H4N6D1A1 | (Hex)1 (HexNAc)4 (Deoxyhexose)1 (NeuAc)1 + (Man)3(GlcNAc)2 | complex | 4 | Sia | 1 | 1 |
|  |  |  | (HexNAc)4 (Deoxyhexose)2 (NeuGc)1 + (Man)3(GlcNAc)2 |  |  |  |  |  |
| 93 | 2785.08 | H6N6D1 | (Hex)3 (HexNAc)4 (Deoxyhexose)1 + (Man)3(GlcNAc)2 | complex | 4 | Gal | 0 | 1 |
| 94 | 2827.09 | H5N4D1A2 | (Hex)2 (HexNAc)2 (Deoxyhexose)1 (NeuAc)2 + (Man)3(GlcNAc)2 | complex | 2 | Sia | 2 | 1 |
|  |  |  | (Hex)1 (HexNAc)2 (Deoxyhexose)2 (NeuAc)1 (NeuGc)1 + (Man)3(GlcNAc)2 |  |  |  |  |  |
|  |  |  | (HexNAc)2 (Deoxyhexose)3 (NeuGc)2 + (Man)3(GlcNAc)2 |  |  |  |  |  |
| 95 | 2842.10 | H6N7 | (Hex)3 (HexNAc)5 + (Man)3(GlcNAc)2 | complex | 5 | Gal | 0 | 0 |
| 96 | 2843.09 | H5N4D1A1G1 | (Hex)2 (HexNAc)2 (Deoxyhexose)1 (NeuAc)1 (NeuGc)1 + (Man)3(GlcNAc)2 | complex | 2 | Sia | 2 | 1 |
|  |  |  | (Hex)3 (HexNAc)2 (NeuAc)2 + (Man)3(GlcNAc)2 |  |  |  |  |  |
|  |  |  | (Hex)1 (HexNAc)2 (Deoxyhexose)2 (NeuGc)2 + (Man)3(GlcNAc)2 |  |  |  |  |  |
| 97 | 2859.08 | H5N4D1G2 | (Hex)2 (HexNAc)2 (Deoxyhexose)1 (NeuGc)2 + (Man)3(GlcNAc)2 | complex | 2 | Sia | 2 | 1 |
|  |  |  | (Hex)3 (HexNAc)2 (NeuAc)1 (NeuGc)1 + (Man)3(GlcNAc)2 |  |  |  |  |  |
| 98 | 2887.11 | H6N5D1A1 | (Hex)3 (HexNAc)3 (Deoxyhexose)1 (NeuAc)1 + (Man)3(GlcNAc)2 | complex | 3 | Sia | 1 | 1 |
|  |  |  | (Hex)2 (HexNAc)3 (Deoxyhexose)2 (NeuGc)1 + (Man)3(GlcNAc)2 |  |  |  |  |  |
| 99 | 3002.14 | H5N4A2G1 | (Hex)2 (HexNAc)2 (NeuAc)2 (NeuGc)1 + (Man)3(GlcNAc)2 | complex | 2 | Sia | 3 | 0 |
|  |  |  | (Hex)9 (HexNAc)1 + (Man)3(GlcNAc)2 |  |  |  |  |  |
|  |  |  | (Hex)1 (HexNAc)2 (Deoxyhexose)1 (NeuAc)1 (NeuGc)2 + (Man)3(GlcNAc)2 |  |  |  |  |  |
|  |  |  | (HexNAc)2 (Deoxyhexose)2 (NeuGc)3 + (Man)3(GlcNAc)2 |  |  |  |  |  |
| 100 | 3034.13 | H5N4G3 | (Hex)2 (HexNAc)2 (NeuGc)3 + (Man)3(GlcNAc)2 | complex | 2 | Sia | 3 | 0 |
| 101 | 3078.16 | H6N5G2 | (Hex)3 (HexNAc)3 (NeuGc)2 + (Man)3(GlcNAc)2 | complex | 3 | Sia | 2 | 0 |
| 102 | 3192.22 | H6N5D1A2 | (Hex)3 (HexNAc)3 (Deoxyhexose)1 (NeuAc)2 + (Man)3(GlcNAc)2 | complex | 3 | Sia | 2 | 1 |
|  |  |  | (Hex)2 (HexNAc)3 (Deoxyhexose)2 (NeuAc)1 (NeuGc)1 + (Man)3(GlcNAc)2 |  |  |  |  |  |
|  |  |  | (Hex)1 (HexNAc)3 (Deoxyhexose)3 (NeuGc)2 + (Man)3(GlcNAc)2 |  |  |  |  |  |
| 103 | 3399.26 | H6N5G3 | (Hex)3 (HexNAc)3 (NeuGc)3 + (Man)3(GlcNAc)2 | complex | 3 | Sia | 3 | 0 |
|  |  |  | (Hex)10 (Deoxyhexose)3 + (Man)3(GlcNAc)2 |  |  |  |  |  |
|  |  |  | (Hex)4 (HexNAc)1 (Deoxyhexose)2 (NeuAc)3 + (Man)3(GlcNAc)2 |  |  |  |  |  |
|  |  |  | (Hex)3 (HexNAc)1 (Deoxyhexose)3 (NeuAc)2 (NeuGc)1 + (Man)3(GlcNAc)2 |  |  |  |  |  |
| 104 | 3497.33 | H6N5D1A3 | (Hex)3 (HexNAc)3 (Deoxyhexose)1 (NeuAc)3 + (Man)3(GlcNAc)2 | complex | 3 | Sia | 3 | 1 |
|  |  |  | (Hex)9 (HexNAc)2 (Deoxyhexose)2 + (Man)3(GlcNAc)2 |  |  |  |  |  |
|  |  |  | (Hex)2 (HexNAc)3 (Deoxyhexose)2 (NeuAc)2 (NeuGc)1 + (Man)3(GlcNAc)2 |  |  |  |  |  |
|  |  |  | (Hex)1 (HexNAc)3 (Deoxyhexose)3 (NeuAc)1 (NeuGc)2 + (Man)3(GlcNAc)2 |  |  |  |  |  |

These peaks were observed at least three samples. Glycan compositions were assigned from Expasy GlycoMod tool (<https://web.expasy.org/glycomod/>) by the *m/z* of the mass peaks; HexNAc: the numbers of HexNAc except core structure or the numbers of antennae of the *N*-glycan structure. Abbreviations is represented the summary numbers of sugars from next column. H: Hex, N: HexNAc, D: Deoxyhexose, A: Neu5Ac, G: Neu5Gc. The right five columns show the glycan types and groups; End: the terminal sugar at the glycan structures, Sialic: the total number of Neu5Ac and Neu5Gc, Fucose: the numbers of deoxyhexose.

**Table S2**. Expression levels of glycoforms identified in all samples (pmol/100 μg protein).

| Glycoform | **1** | **2** | **3** | **4** | **5** | **6** | **7** | **8** | **9** | **10** |
| --- | --- | --- | --- | --- | --- | --- | --- | --- | --- | --- |
| *m/z* | 1340.55 | 1486.6 | 1502.6 | 1543.63 | 1648.66 | 1664.65 | 1689.68 | 1705.68 | 1746.7 | 1810.71 |
| Abbreviation | H3N2 | H3N2D1 | H4N2 | H3N3 | H4N2D1 | H5N2 | H3N3D1 | H4N3 | H3N4 | H5N2D1 |
| Brain 1 | 2.34 | 14.89 | 5.51 | 0.00 | 2.26 | 231.14 | 4.45 | 0.00 | 2.08 | 2.56 |
| Brain 2 | 0.18 | 1.62 | 0.53 | 0.00 | 0.16 | 34.17 | 0.33 | 0.00 | 0.16 | 0.21 |
| Brain 3 | 0.11 | 0.81 | 0.31 | 0.00 | 0.09 | 20.58 | 0.18 | 0.00 | 0.12 | 0.12 |
| Brain 4 | 0.00 | 1.22 | 0.38 | 0.00 | 0.00 | 37.33 | 0.28 | 0.00 | 0.00 | 0.00 |
| Brain 5 | 0.13 | 1.09 | 0.37 | 0.00 | 0.00 | 25.50 | 0.24 | 0.00 | 0.11 | 0.17 |
| Femur 1 | 5.86 | 5.20 | 4.60 | 0.00 | 0.00 | 31.08 | 0.69 | 0.00 | 0.00 | 0.00 |
| Femur 2 | 13.78 | 15.13 | 9.78 | 0.00 | 0.00 | 50.18 | 1.99 | 1.10 | 0.00 | 0.00 |
| Femur 3 | 5.47 | 5.02 | 5.82 | 0.00 | 0.00 | 36.35 | 1.06 | 0.00 | 0.00 | 0.00 |
| Femur 4 | 6.92 | 6.38 | 6.18 | 0.00 | 0.00 | 32.70 | 0.00 | 0.00 | 0.00 | 0.00 |
| Femur 5 | 15.75 | 21.94 | 13.92 | 0.00 | 0.00 | 84.45 | 3.13 | 1.24 | 0.00 | 0.94 |
| Heart 1 | 2.85 | 4.67 | 3.55 | 1.25 | 1.18 | 16.43 | 1.46 | 0.00 | 0.00 | 1.24 |
| Heart 2 | 0.41 | 0.72 | 0.48 | 0.00 | 0.15 | 9.57 | 0.30 | 0.00 | 0.17 | 0.17 |
| Heart 3 | 0.70 | 1.14 | 0.67 | 0.14 | 0.16 | 17.85 | 0.48 | 0.00 | 0.17 | 0.00 |
| Heart 4 | 0.82 | 1.18 | 0.57 | 0.15 | 0.16 | 14.51 | 0.34 | 0.00 | 0.24 | 0.17 |
| Heart 5 | 1.01 | 2.27 | 1.15 | 0.36 | 0.35 | 26.78 | 1.01 | 0.00 | 0.00 | 0.00 |
| Intestines 1 | 35.37 | 36.60 | 12.37 | 17.88 | 2.87 | 82.39 | 46.07 | 8.75 | 13.81 | 2.97 |
| Intestines 2 | 7.92 | 8.90 | 7.49 | 2.07 | 1.13 | 107.12 | 10.56 | 2.16 | 1.55 | 1.62 |
| Intestines 3 | 11.68 | 13.58 | 9.86 | 3.45 | 1.25 | 128.30 | 14.68 | 3.62 | 3.13 | 1.95 |
| Intestines 4 | 9.58 | 10.61 | 7.06 | 3.88 | 0.00 | 70.44 | 8.96 | 7.19 | 3.14 | 1.57 |
| Intestines 5 | 10.62 | 11.82 | 8.35 | 1.48 | 1.15 | 115.93 | 12.96 | 1.81 | 1.17 | 1.77 |
| Kidney 1 | 30.68 | 36.00 | 22.97 | 2.52 | 4.45 | 101.78 | 6.63 | 2.02 | 2.90 | 3.00 |
| Kidney 2 | 17.98 | 21.65 | 16.16 | 1.47 | 0.00 | 70.52 | 3.59 | 1.17 | 1.77 | 1.72 |
| Kidney 3 | 20.42 | 24.14 | 17.61 | 1.42 | 0.00 | 94.42 | 3.76 | 0.00 | 1.60 | 1.75 |
| Kidney 4 | 11.83 | 13.15 | 7.61 | 0.63 | 0.79 | 41.26 | 1.53 | 0.00 | 0.00 | 0.76 |
| Kidney 5 | 14.14 | 17.19 | 10.28 | 0.83 | 1.36 | 51.05 | 2.43 | 0.75 | 0.97 | 1.27 |
| Liver 1 | 0.00 | 1.89 | 8.13 | 3.22 | 0.00 | 120.85 | 1.40 | 0.00 | 5.61 | 0.00 |
| Liver 2 | 1.41 | 0.28 | 0.92 | 0.35 | 0.00 | 16.87 | 0.00 | 0.00 | 0.92 | 0.00 |
| Liver 3 | 1.21 | 0.19 | 0.92 | 0.26 | 0.00 | 11.89 | 0.15 | 0.10 | 0.54 | 0.00 |
| Liver 4 | 1.30 | 0.60 | 1.07 | 0.29 | 0.00 | 17.50 | 0.09 | 0.00 | 0.82 | 0.00 |
| Liver 5 | 0.67 | 0.12 | 0.48 | 0.12 | 0.00 | 10.71 | 0.08 | 0.00 | 0.17 | 0.00 |
| Lung 1 | 7.52 | 8.73 | 5.85 | 0.64 | 0.00 | 91.48 | 1.39 | 1.30 | 0.00 | 0.82 |
| Lung 2 | 14.14 | 29.34 | 8.68 | 1.06 | 0.95 | 84.49 | 2.01 | 1.42 | 0.00 | 1.48 |
| Lung 3 | 16.82 | 21.04 | 12.74 | 1.15 | 0.00 | 102.27 | 3.89 | 1.98 | 0.00 | 1.48 |
| Lung 4 | 3.65 | 4.45 | 2.53 | 0.00 | 0.00 | 41.31 | 0.87 | 0.00 | 0.00 | 0.00 |
| Lung 5 | 11.76 | 15.42 | 9.53 | 1.30 | 0.94 | 107.38 | 3.29 | 2.21 | 0.00 | 1.35 |
| Muscle 1 | 1.65 | 0.66 | 2.55 | 0.00 | 0.00 | 15.05 | 0.00 | 0.00 | 0.00 | 0.00 |
| Muscle 2 | 2.02 | 0.61 | 3.01 | 0.00 | 0.00 | 15.34 | 0.00 | 0.00 | 0.00 | 0.00 |
| Muscle 3 | 6.27 | 0.00 | 5.40 | 0.00 | 0.00 | 16.19 | 0.00 | 0.00 | 0.00 | 0.00 |
| Muscle 4 | 4.16 | 0.00 | 3.25 | 0.00 | 0.00 | 10.91 | 0.00 | 0.00 | 0.00 | 0.00 |
| Muscle 5 | 8.16 | 1.65 | 8.01 | 0.00 | 0.81 | 25.85 | 0.00 | 0.00 | 0.00 | 0.00 |
| Ovary 1 | 8.81 | 11.11 | 8.68 | 0.00 | 0.00 | 81.31 | 4.15 | 0.00 | 0.00 | 0.00 |
| Ovary 2 | 6.69 | 8.44 | 4.94 | 0.35 | 0.24 | 40.55 | 2.22 | 0.49 | 0.21 | 0.00 |
| Ovary 3 | 5.92 | 7.91 | 4.99 | 0.70 | 0.00 | 52.81 | 3.01 | 0.88 | 0.00 | 0.00 |
| Ovary 4 | 2.24 | 2.94 | 2.27 | 0.27 | 0.00 | 27.18 | 1.77 | 0.34 | 0.25 | 0.00 |
| Ovary 5 | 6.72 | 9.00 | 6.13 | 0.54 | 0.54 | 66.04 | 3.32 | 0.75 | 0.41 | 0.00 |
| Pancreas 1 | 17.29 | 13.87 | 30.87 | 13.57 | 0.00 | 715.76 | 52.68 | 0.00 | 40.10 | 4.19 |
| Pancreas 2 | 6.84 | 2.76 | 7.96 | 5.74 | 0.00 | 109.34 | 14.39 | 1.36 | 7.07 | 0.00 |
| Pancreas 3 | 5.82 | 6.09 | 9.70 | 7.16 | 0.00 | 354.89 | 19.37 | 6.00 | 23.57 | 0.00 |
| Pancreas 4 | 5.11 | 5.69 | 9.24 | 6.27 | 0.00 | 306.83 | 18.61 | 6.40 | 22.91 | 0.00 |
| Pancreas 5 | 10.06 | 5.32 | 14.56 | 5.56 | 0.00 | 439.98 | 25.94 | 0.00 | 16.75 | 0.00 |
| Skin 1 | 6.41 | 4.35 | 4.63 | 2.35 | 0.00 | 28.80 | 1.28 | 1.39 | 0.00 | 0.00 |
| Skin 2 | 16.85 | 9.59 | 10.30 | 3.70 | 0.77 | 63.65 | 4.68 | 2.61 | 0.00 | 0.00 |
| Skin 3 | 20.49 | 14.18 | 14.41 | 9.96 | 3.40 | 107.66 | 6.76 | 4.67 | 1.93 | 0.00 |
| Skin 4 | 29.27 | 20.88 | 23.10 | 10.11 | 1.58 | 169.95 | 13.22 | 9.61 | 2.52 | 1.07 |
| Skin 5 | 35.99 | 23.80 | 24.02 | 9.84 | 0.00 | 154.85 | 13.68 | 6.92 | 2.25 | 1.73 |
| Spleen 1 | 36.10 | 58.00 | 22.90 | 1.36 | 3.78 | 71.48 | 3.23 | 1.21 | 0.00 | 3.60 |
| Spleen 2 | 38.91 | 62.49 | 25.14 | 1.13 | 5.21 | 60.56 | 3.07 | 1.36 | 0.00 | 4.71 |
| Spleen 3 | 17.54 | 29.41 | 11.50 | 1.41 | 1.92 | 35.61 | 3.50 | 1.07 | 0.00 | 1.86 |
| Spleen 4 | 31.38 | 37.48 | 16.26 | 0.83 | 2.44 | 59.09 | 2.57 | 0.99 | 0.00 | 2.48 |
| Spleen 5 | 33.69 | 40.91 | 19.43 | 1.50 | 3.14 | 49.06 | 2.63 | 1.79 | 0.00 | 2.60 |
| Stomach 1 | 0.00 | 19.48 | 6.29 | 6.87 | 0.00 | 115.56 | 20.03 | 2.71 | 4.59 | 0.00 |
| Stomach 2 | 4.62 | 6.08 | 3.51 | 1.67 | 0.00 | 107.67 | 7.73 | 0.68 | 0.00 | 0.00 |
| Stomach 3 | 94.25 | 20.00 | 6.89 | 65.18 | 0.74 | 6.34 | 36.07 | 29.50 | 29.60 | 0.00 |
| Stomach 4 | 6.23 | 9.33 | 2.51 | 3.04 | 0.00 | 100.27 | 14.61 | 0.84 | 0.00 | 0.00 |
| Stomach 5 | 2.65 | 4.61 | 2.00 | 1.00 | 0.00 | 97.88 | 4.36 | 0.00 | 0.60 | 0.00 |
| Testis 1 | 0.00 | 16.92 | 13.45 | 5.79 | 1.03 | 147.53 | 21.52 | 0.00 | 13.75 | 1.33 |
| Testis 2 | 16.28 | 20.87 | 17.30 | 8.83 | 0.00 | 172.30 | 32.38 | 2.27 | 14.70 | 1.11 |
| Testis 3 | 12.37 | 14.37 | 13.02 | 7.58 | 0.91 | 98.85 | 23.27 | 1.60 | 11.71 | 0.32 |
| Testis 4 | 11.75 | 16.03 | 11.71 | 7.83 | 0.90 | 104.20 | 22.60 | 1.82 | 12.97 | 0.00 |
| Testis 5 | 11.49 | 15.20 | 10.16 | 7.20 | 0.60 | 90.71 | 18.47 | 1.56 | 10.31 | 0.00 |
| Thyroid 1 | 11.46 | 7.16 | 228.69 | 0.00 | 0.00 | 733.44 | 11.27 | 0.00 | 0.00 | 0.00 |
| Thyroid 2 | 10.53 | 6.44 | 201.71 | 0.00 | 0.00 | 639.96 | 0.00 | 0.00 | 0.00 | 0.00 |
| Thyroid 3 | 13.49 | 8.64 | 242.05 | 0.00 | 0.00 | 740.11 | 0.00 | 0.00 | 0.00 | 0.00 |
| Thyroid 4 | 11.97 | 8.93 | 269.18 | 0.00 | 0.00 | 903.13 | 0.00 | 0.00 | 0.00 | 0.00 |
| Thyroid 5 | 16.11 | 10.71 | 289.71 | 0.00 | 0.00 | 900.72 | 0.00 | 0.00 | 0.00 | 0.00 |
| Uterus 1 | 4.34 | 3.73 | 3.56 | 0.91 | 0.00 | 72.99 | 1.47 | 0.92 | 0.00 | 0.00 |
| Uterus 2 | 23.55 | 26.73 | 17.24 | 2.00 | 1.71 | 121.54 | 9.27 | 2.15 | 0.00 | 0.00 |
| Uterus 3 | 30.04 | 24.94 | 21.83 | 2.82 | 1.96 | 144.09 | 11.84 | 3.39 | 1.86 | 0.00 |
| Uterus 4 | 18.02 | 15.51 | 13.87 | 2.51 | 1.10 | 134.49 | 9.28 | 2.69 | 2.08 | 0.00 |
| Uterus 5 | 18.99 | 14.04 | 12.51 | 2.14 | 0.95 | 113.31 | 7.84 | 2.31 | 1.65 | 0.00 |
| Serum 1 | 0.00 | 0.00 | 0.00 | 0.00 | 0.00 | 10.76 | 0.00 | 0.00 | 0.00 | 0.00 |
| Serum 2 | 0.00 | 0.00 | 0.00 | 0.00 | 0.00 | 5.15 | 0.00 | 0.00 | 0.00 | 0.00 |
| Serum 3 | 0.00 | 0.00 | 0.00 | 0.00 | 0.00 | 8.58 | 0.00 | 0.00 | 0.00 | 0.00 |
| Serum 4 | 0.00 | 0.00 | 0.00 | 0.00 | 0.00 | 7.61 | 0.00 | 0.00 | 0.00 | 0.00 |
| Serum 5 | 0.00 | 0.00 | 0.00 | 0.00 | 0.00 | 6.53 | 0.00 | 0.00 | 0.00 | 0.00 |
| Exosome 1 | 0.00 | 0.00 | 0.00 | 0.00 | 0.00 | 10.77 | 0.00 | 0.00 | 0.00 | 0.00 |
| Exosome 2 | 0.00 | 0.00 | 0.00 | 0.00 | 0.00 | 7.55 | 0.00 | 0.00 | 0.00 | 0.00 |
| Exosome 3 | 0.00 | 0.00 | 0.00 | 0.00 | 0.00 | 11.54 | 0.00 | 0.00 | 0.00 | 0.00 |
| Exosome 4 | 0.00 | 0.00 | 0.00 | 0.00 | 0.00 | 26.81 | 0.00 | 0.00 | 0.00 | 0.00 |
| Exosome 5 | 0.00 | 0.00 | 0.00 | 0.00 | 0.00 | 19.44 | 0.00 | 0.00 | 0.00 | 0.00 |

| Glycoform | **11** | **12** | **13** | **14** | **15** | **16** | **17** | **18** | **19** | **20** |
| --- | --- | --- | --- | --- | --- | --- | --- | --- | --- | --- |
| *m/z* | 1826.71 | 1851.74 | 1867.73 | 1892.76 | 1908.76 | 1949.78 | 1988.76 | 1997.79 | 2010.79 | 2013.79 |
| Abbreviation | H6N2 | H4N3D1 | H5N3 | H3N4D1 | H4N4 | H3N5 | H7N2 | H4N3D2 | H4N3A1 | H5N3D1 |
| Brain 1 | 67.45 | 4.48 | 3.59 | 53.09 | 1.31 | 4.24 | 26.97 | 1.85 | 0.00 | 4.04 |
| Brain 2 | 11.17 | 0.35 | 0.35 | 6.81 | 0.00 | 0.35 | 4.56 | 0.19 | 0.14 | 0.34 |
| Brain 3 | 6.57 | 0.20 | 0.19 | 3.73 | 0.00 | 0.19 | 2.59 | 0.11 | 0.13 | 0.19 |
| Brain 4 | 9.57 | 0.27 | 0.00 | 6.01 | 0.00 | 0.00 | 2.75 | 0.00 | 0.00 | 0.26 |
| Brain 5 | 8.32 | 0.28 | 0.23 | 5.27 | 0.00 | 0.23 | 3.25 | 0.15 | 0.12 | 0.27 |
| Femur 1 | 15.10 | 0.00 | 0.00 | 0.79 | 0.00 | 0.00 | 7.84 | 0.00 | 0.00 | 0.00 |
| Femur 2 | 36.12 | 0.00 | 1.87 | 2.78 | 0.00 | 0.00 | 17.57 | 0.00 | 2.24 | 0.00 |
| Femur 3 | 24.48 | 0.00 | 0.92 | 1.68 | 0.00 | 0.00 | 11.34 | 0.00 | 0.00 | 0.00 |
| Femur 4 | 19.42 | 0.00 | 0.00 | 1.33 | 0.00 | 0.00 | 8.57 | 0.00 | 1.27 | 0.00 |
| Femur 5 | 64.27 | 1.59 | 2.30 | 4.54 | 0.00 | 0.00 | 29.69 | 0.00 | 0.84 | 1.47 |
| Heart 1 | 13.26 | 0.00 | 0.00 | 1.92 | 0.00 | 0.00 | 4.18 | 0.00 | 0.00 | 0.00 |
| Heart 2 | 9.60 | 0.00 | 0.00 | 0.52 | 0.00 | 0.00 | 2.11 | 0.00 | 0.00 | 0.00 |
| Heart 3 | 14.80 | 0.23 | 0.00 | 0.90 | 0.00 | 0.00 | 3.32 | 0.00 | 0.00 | 0.00 |
| Heart 4 | 14.38 | 0.14 | 0.14 | 0.46 | 0.00 | 0.00 | 3.57 | 0.00 | 0.00 | 0.00 |
| Heart 5 | 19.80 | 0.00 | 0.00 | 1.00 | 0.00 | 0.00 | 3.90 | 0.00 | 0.00 | 0.00 |
| Intestines 1 | 19.05 | 5.97 | 4.50 | 54.75 | 15.77 | 0.00 | 9.07 | 0.00 | 0.00 | 2.71 |
| Intestines 2 | 35.07 | 2.01 | 1.85 | 10.22 | 2.43 | 1.53 | 21.96 | 0.00 | 0.55 | 1.08 |
| Intestines 3 | 41.43 | 3.18 | 3.70 | 12.71 | 5.32 | 0.00 | 21.77 | 0.00 | 0.00 | 1.75 |
| Intestines 4 | 28.27 | 2.30 | 10.45 | 9.40 | 9.96 | 1.88 | 16.28 | 0.00 | 0.65 | 1.07 |
| Intestines 5 | 43.71 | 3.33 | 1.89 | 8.41 | 1.20 | 0.00 | 27.07 | 0.00 | 0.00 | 1.74 |
| Kidney 1 | 31.59 | 1.59 | 1.26 | 9.14 | 1.15 | 1.57 | 18.72 | 0.00 | 0.00 | 1.31 |
| Kidney 2 | 21.21 | 0.87 | 0.93 | 5.42 | 0.00 | 0.98 | 14.52 | 0.00 | 0.00 | 0.88 |
| Kidney 3 | 16.87 | 0.00 | 0.00 | 5.37 | 0.00 | 1.04 | 15.74 | 0.00 | 0.00 | 0.00 |
| Kidney 4 | 13.77 | 0.00 | 0.00 | 2.06 | 0.00 | 0.00 | 8.36 | 0.00 | 0.00 | 0.00 |
| Kidney 5 | 22.78 | 0.54 | 0.57 | 3.71 | 0.51 | 0.63 | 13.95 | 0.00 | 0.00 | 0.60 |
| Liver 1 | 20.26 | 0.00 | 0.00 | 6.79 | 0.00 | 0.97 | 28.09 | 0.00 | 0.00 | 0.00 |
| Liver 2 | 2.25 | 0.00 | 0.00 | 0.92 | 0.00 | 0.00 | 1.78 | 0.00 | 0.00 | 0.00 |
| Liver 3 | 3.17 | 0.00 | 0.00 | 1.17 | 0.00 | 0.07 | 3.40 | 0.00 | 0.16 | 0.00 |
| Liver 4 | 3.06 | 0.00 | 0.00 | 0.53 | 0.00 | 0.18 | 3.57 | 0.00 | 0.16 | 0.00 |
| Liver 5 | 1.40 | 0.00 | 0.00 | 0.50 | 0.00 | 0.00 | 1.77 | 0.00 | 0.07 | 0.00 |
| Lung 1 | 50.05 | 1.21 | 3.05 | 3.34 | 0.00 | 0.00 | 23.84 | 0.00 | 0.00 | 0.00 |
| Lung 2 | 33.82 | 1.60 | 3.19 | 4.78 | 0.00 | 0.00 | 18.16 | 0.00 | 3.27 | 1.56 |
| Lung 3 | 52.67 | 3.56 | 3.99 | 14.29 | 0.00 | 0.00 | 29.38 | 0.00 | 2.19 | 3.37 |
| Lung 4 | 14.36 | 0.85 | 1.24 | 3.68 | 0.00 | 0.00 | 6.71 | 0.00 | 0.00 | 1.18 |
| Lung 5 | 41.19 | 2.38 | 4.74 | 6.21 | 1.12 | 0.00 | 19.92 | 0.00 | 1.42 | 2.67 |
| Muscle 1 | 9.15 | 0.00 | 0.00 | 0.00 | 0.00 | 0.00 | 2.98 | 0.00 | 0.00 | 0.00 |
| Muscle 2 | 8.61 | 0.00 | 0.00 | 0.00 | 0.00 | 0.00 | 3.40 | 0.00 | 0.00 | 0.00 |
| Muscle 3 | 12.44 | 0.00 | 0.00 | 0.00 | 0.00 | 0.00 | 3.98 | 0.00 | 0.00 | 0.00 |
| Muscle 4 | 7.86 | 0.00 | 0.00 | 0.00 | 0.00 | 0.00 | 1.97 | 0.00 | 0.00 | 0.00 |
| Muscle 5 | 14.26 | 0.00 | 0.00 | 0.00 | 0.00 | 0.00 | 5.87 | 0.00 | 0.00 | 0.00 |
| Ovary 1 | 40.10 | 0.00 | 2.54 | 20.30 | 0.00 | 0.00 | 17.18 | 0.00 | 0.00 | 0.00 |
| Ovary 2 | 27.80 | 0.92 | 0.92 | 4.55 | 0.00 | 0.00 | 10.38 | 0.00 | 0.46 | 0.42 |
| Ovary 3 | 33.91 | 1.39 | 1.56 | 11.09 | 0.00 | 0.00 | 16.36 | 0.00 | 0.57 | 0.69 |
| Ovary 4 | 23.46 | 0.00 | 0.70 | 11.09 | 0.32 | 0.00 | 9.85 | 0.00 | 0.34 | 0.00 |
| Ovary 5 | 38.83 | 1.30 | 1.05 | 22.45 | 0.00 | 0.00 | 21.31 | 0.00 | 0.58 | 0.72 |
| Pancreas 1 | 78.96 | 6.40 | 7.15 | 158.82 | 0.00 | 3.42 | 65.46 | 0.00 | 0.00 | 6.50 |
| Pancreas 2 | 18.33 | 0.00 | 0.00 | 45.38 | 0.00 | 0.00 | 3.26 | 0.00 | 0.00 | 0.00 |
| Pancreas 3 | 48.55 | 2.67 | 5.15 | 76.83 | 1.45 | 1.31 | 43.02 | 0.00 | 1.47 | 2.89 |
| Pancreas 4 | 43.45 | 3.25 | 5.07 | 76.99 | 1.84 | 0.00 | 37.05 | 0.00 | 0.00 | 3.22 |
| Pancreas 5 | 35.53 | 0.00 | 2.49 | 76.15 | 0.00 | 0.00 | 32.17 | 0.00 | 0.00 | 1.82 |
| Skin 1 | 13.82 | 1.50 | 1.30 | 10.13 | 0.00 | 0.00 | 5.71 | 0.00 | 0.00 | 0.00 |
| Skin 2 | 26.12 | 3.22 | 2.01 | 50.53 | 3.07 | 0.00 | 12.29 | 0.00 | 0.00 | 1.29 |
| Skin 3 | 37.51 | 4.41 | 4.21 | 18.83 | 4.85 | 0.00 | 22.70 | 0.00 | 0.00 | 2.09 |
| Skin 4 | 50.02 | 9.74 | 11.92 | 29.71 | 0.00 | 0.00 | 27.84 | 0.00 | 0.00 | 4.20 |
| Skin 5 | 54.31 | 8.27 | 7.77 | 28.02 | 6.01 | 0.00 | 29.70 | 0.00 | 0.00 | 3.84 |
| Spleen 1 | 24.60 | 1.21 | 1.67 | 2.95 | 0.00 | 0.00 | 13.64 | 0.00 | 0.00 | 0.00 |
| Spleen 2 | 28.53 | 1.14 | 2.07 | 3.62 | 0.00 | 0.00 | 19.33 | 0.00 | 2.38 | 1.35 |
| Spleen 3 | 9.49 | 0.00 | 1.31 | 3.77 | 0.00 | 0.00 | 8.75 | 0.00 | 1.62 | 0.81 |
| Spleen 4 | 30.56 | 0.85 | 1.66 | 2.38 | 0.00 | 0.00 | 19.77 | 0.00 | 2.48 | 0.89 |
| Spleen 5 | 25.08 | 1.52 | 2.20 | 3.11 | 0.00 | 0.00 | 14.37 | 0.00 | 2.22 | 1.20 |
| Stomach 1 | 66.11 | 3.72 | 2.71 | 19.90 | 2.09 | 2.70 | 16.43 | 0.00 | 0.00 | 1.29 |
| Stomach 2 | 55.54 | 1.88 | 0.71 | 7.50 | 0.53 | 0.51 | 13.03 | 0.00 | 0.00 | 0.53 |
| Stomach 3 | 1.32 | 5.56 | 13.01 | 21.70 | 36.83 | 2.76 | 0.59 | 0.00 | 1.28 | 0.86 |
| Stomach 4 | 59.27 | 1.74 | 1.07 | 14.04 | 0.00 | 1.54 | 13.95 | 0.00 | 0.00 | 0.68 |
| Stomach 5 | 39.02 | 1.17 | 0.53 | 7.02 | 0.32 | 0.00 | 10.43 | 0.00 | 0.00 | 0.33 |
| Testis 1 | 49.34 | 2.76 | 1.23 | 127.18 | 1.76 | 16.35 | 39.38 | 0.00 | 0.00 | 1.52 |
| Testis 2 | 60.53 | 4.71 | 1.47 | 127.15 | 1.81 | 17.03 | 36.06 | 0.00 | 0.78 | 2.56 |
| Testis 3 | 59.20 | 4.05 | 1.30 | 95.49 | 1.53 | 14.18 | 31.16 | 0.00 | 0.89 | 2.12 |
| Testis 4 | 51.64 | 3.55 | 1.42 | 101.49 | 1.85 | 16.06 | 34.82 | 0.00 | 0.81 | 2.24 |
| Testis 5 | 41.66 | 2.70 | 1.19 | 81.05 | 1.30 | 12.38 | 26.67 | 0.00 | 0.66 | 1.54 |
| Thyroid 1 | 514.44 | 16.67 | 0.00 | 1.26 | 0.00 | 0.00 | 230.46 | 0.00 | 1.67 | 2.56 |
| Thyroid 2 | 428.09 | 16.61 | 0.00 | 1.18 | 0.00 | 0.00 | 162.14 | 0.00 | 1.72 | 0.00 |
| Thyroid 3 | 458.29 | 21.81 | 0.00 | 1.27 | 0.00 | 0.00 | 159.91 | 0.00 | 1.78 | 0.00 |
| Thyroid 4 | 617.36 | 19.29 | 0.00 | 1.43 | 0.00 | 0.00 | 258.45 | 0.00 | 1.53 | 2.54 |
| Thyroid 5 | 594.10 | 29.99 | 0.00 | 1.72 | 0.00 | 0.00 | 218.11 | 0.00 | 2.71 | 0.00 |
| Uterus 1 | 18.73 | 0.72 | 1.11 | 5.75 | 0.00 | 0.00 | 12.45 | 0.00 | 0.66 | 0.00 |
| Uterus 2 | 72.26 | 3.28 | 3.68 | 13.28 | 0.99 | 0.00 | 34.64 | 0.00 | 1.22 | 1.84 |
| Uterus 3 | 71.62 | 4.75 | 4.44 | 18.20 | 0.00 | 0.00 | 32.64 | 0.00 | 1.31 | 2.40 |
| Uterus 4 | 71.06 | 4.12 | 4.35 | 28.34 | 0.00 | 0.00 | 41.05 | 0.00 | 0.84 | 2.23 |
| Uterus 5 | 52.41 | 3.34 | 2.80 | 23.14 | 0.00 | 0.56 | 29.24 | 0.00 | 0.84 | 1.64 |
| Serum 1 | 6.42 | 0.00 | 0.00 | 108.54 | 0.00 | 0.00 | 0.00 | 0.00 | 0.00 | 0.00 |
| Serum 2 | 3.55 | 0.00 | 0.00 | 53.81 | 0.00 | 0.00 | 0.00 | 0.00 | 0.00 | 0.00 |
| Serum 3 | 4.80 | 0.00 | 0.00 | 76.86 | 0.00 | 0.00 | 0.00 | 0.00 | 0.00 | 0.00 |
| Serum 4 | 4.97 | 0.00 | 0.00 | 79.48 | 0.00 | 0.00 | 0.00 | 0.00 | 0.00 | 0.00 |
| Serum 5 | 4.44 | 0.00 | 0.00 | 79.51 | 0.00 | 0.00 | 0.00 | 0.00 | 0.00 | 0.00 |
| Exosome 1 | 3.72 | 0.00 | 0.00 | 10.17 | 0.00 | 0.00 | 1.12 | 0.00 | 0.66 | 0.00 |
| Exosome 2 | 2.38 | 0.00 | 0.00 | 16.92 | 0.00 | 0.00 | 0.69 | 0.00 | 0.00 | 0.00 |
| Exosome 3 | 3.80 | 0.00 | 0.00 | 10.60 | 0.00 | 0.00 | 1.14 | 0.00 | 0.00 | 0.00 |
| Exosome 4 | 7.20 | 0.00 | 0.00 | 35.68 | 0.00 | 0.00 | 2.20 | 0.00 | 0.00 | 0.00 |
| Exosome 5 | 5.02 | 0.00 | 0.00 | 25.96 | 0.00 | 0.00 | 1.59 | 0.00 | 0.00 | 0.00 |

| Glycoform | **21** | **22** | **23** | **24** | **25** | **26** | **27** | **28** | **29** | **30** |
| --- | --- | --- | --- | --- | --- | --- | --- | --- | --- | --- |
| *m/z* | 2026.79 | 2029.78 | 2054.82 | 2070.81 | 2095.84 | 2111.84 | 2150.81 | 2156.85 | 2172.84 | 2175.84 |
| Abbreviation | H4N3G1 | H6N3 | H4N4D1 | H5N4 | H3N5D1 | H4N5 | H8N2 | H4N3D1A1 | H5N3A1 | H6N3D1 |
| Brain 1 | 0.00 | 0.00 | 7.83 | 5.05 | 56.00 | 0.00 | 12.60 | 0.00 | 2.38 | 1.62 |
| Brain 2 | 0.00 | 0.00 | 0.71 | 0.52 | 7.19 | 0.00 | 2.67 | 0.00 | 2.78 | 0.00 |
| Brain 3 | 0.00 | 0.00 | 0.38 | 0.28 | 4.18 | 0.00 | 1.48 | 0.00 | 2.89 | 0.00 |
| Brain 4 | 0.00 | 0.00 | 0.49 | 0.88 | 6.09 | 0.00 | 1.18 | 0.00 | 1.35 | 0.00 |
| Brain 5 | 0.00 | 0.00 | 0.54 | 0.34 | 5.78 | 0.00 | 1.69 | 0.00 | 2.49 | 0.00 |
| Femur 1 | 0.00 | 0.00 | 0.69 | 0.00 | 0.00 | 0.00 | 11.57 | 0.00 | 2.61 | 0.00 |
| Femur 2 | 0.00 | 1.53 | 3.29 | 0.00 | 1.37 | 0.00 | 21.95 | 0.00 | 6.14 | 0.00 |
| Femur 3 | 0.00 | 0.00 | 1.80 | 0.00 | 0.00 | 0.00 | 17.45 | 0.00 | 4.37 | 0.00 |
| Femur 4 | 0.00 | 0.00 | 1.72 | 1.96 | 0.00 | 0.00 | 11.82 | 0.00 | 6.40 | 0.00 |
| Femur 5 | 0.00 | 2.00 | 4.24 | 2.89 | 2.05 | 0.00 | 38.30 | 0.00 | 3.86 | 0.00 |
| Heart 1 | 0.00 | 0.00 | 1.12 | 0.00 | 0.00 | 0.00 | 4.18 | 0.00 | 3.13 | 0.00 |
| Heart 2 | 0.00 | 0.00 | 0.26 | 0.00 | 0.19 | 0.00 | 3.96 | 0.00 | 0.98 | 0.00 |
| Heart 3 | 0.00 | 0.00 | 0.34 | 0.26 | 0.25 | 0.00 | 5.62 | 0.00 | 1.15 | 0.00 |
| Heart 4 | 0.00 | 0.00 | 0.17 | 0.16 | 0.20 | 0.00 | 6.13 | 0.00 | 1.31 | 0.00 |
| Heart 5 | 0.00 | 0.00 | 0.42 | 0.00 | 0.43 | 0.00 | 5.51 | 0.00 | 1.44 | 0.00 |
| Intestines 1 | 0.00 | 0.00 | 29.38 | 10.18 | 24.36 | 21.27 | 7.72 | 0.00 | 4.39 | 0.00 |
| Intestines 2 | 0.00 | 1.32 | 5.42 | 3.93 | 7.57 | 6.08 | 19.98 | 0.00 | 4.00 | 0.00 |
| Intestines 3 | 0.00 | 1.67 | 4.11 | 11.07 | 9.43 | 8.60 | 19.10 | 0.00 | 3.85 | 0.00 |
| Intestines 4 | 0.69 | 2.22 | 5.21 | 12.88 | 6.80 | 0.00 | 14.59 | 0.00 | 8.15 | 0.00 |
| Intestines 5 | 0.00 | 1.73 | 3.90 | 4.48 | 6.41 | 6.13 | 20.41 | 0.52 | 3.24 | 0.00 |
| Kidney 1 | 0.00 | 0.00 | 3.28 | 3.96 | 3.56 | 1.59 | 18.51 | 0.00 | 3.00 | 1.80 |
| Kidney 2 | 0.00 | 0.00 | 1.98 | 1.81 | 1.82 | 1.15 | 15.98 | 0.00 | 7.55 | 1.23 |
| Kidney 3 | 0.00 | 0.00 | 1.77 | 1.43 | 1.71 | 1.00 | 17.10 | 0.00 | 2.66 | 1.30 |
| Kidney 4 | 0.00 | 0.00 | 0.83 | 0.87 | 0.00 | 0.00 | 10.94 | 0.00 | 5.74 | 0.00 |
| Kidney 5 | 0.00 | 0.00 | 1.26 | 1.47 | 1.33 | 0.86 | 18.14 | 0.00 | 3.34 | 1.16 |
| Liver 1 | 1.67 | 0.00 | 0.00 | 0.00 | 1.50 | 0.00 | 36.90 | 0.00 | 2.05 | 0.00 |
| Liver 2 | 0.17 | 0.00 | 0.00 | 0.23 | 0.15 | 0.00 | 1.56 | 0.00 | 1.64 | 0.00 |
| Liver 3 | 0.19 | 0.00 | 0.00 | 0.10 | 0.17 | 0.00 | 4.54 | 0.00 | 3.56 | 0.00 |
| Liver 4 | 0.21 | 0.00 | 0.00 | 0.00 | 0.19 | 0.00 | 3.70 | 0.00 | 3.14 | 0.00 |
| Liver 5 | 0.09 | 0.00 | 0.00 | 0.00 | 0.11 | 0.00 | 1.89 | 0.00 | 2.24 | 0.00 |
| Lung 1 | 0.00 | 16.78 | 2.31 | 2.54 | 1.02 | 0.00 | 24.67 | 0.86 | 4.64 | 1.18 |
| Lung 2 | 2.35 | 14.64 | 3.77 | 2.09 | 1.38 | 0.00 | 17.18 | 3.79 | 6.60 | 0.00 |
| Lung 3 | 2.50 | 18.53 | 13.85 | 4.35 | 2.60 | 0.00 | 29.77 | 2.27 | 8.14 | 2.63 |
| Lung 4 | 0.75 | 2.89 | 2.99 | 0.00 | 0.76 | 0.00 | 5.32 | 0.00 | 3.05 | 0.76 |
| Lung 5 | 1.77 | 15.04 | 4.10 | 4.08 | 2.69 | 0.00 | 13.37 | 1.28 | 5.66 | 1.43 |
| Muscle 1 | 0.00 | 0.00 | 0.00 | 0.00 | 1.61 | 0.00 | 6.48 | 0.00 | 7.24 | 0.00 |
| Muscle 2 | 0.00 | 0.00 | 0.00 | 0.00 | 1.07 | 0.00 | 5.89 | 0.00 | 3.55 | 0.00 |
| Muscle 3 | 0.00 | 0.00 | 0.00 | 0.00 | 0.00 | 0.00 | 7.38 | 0.00 | 6.10 | 0.00 |
| Muscle 4 | 0.00 | 0.00 | 0.00 | 0.00 | 0.00 | 0.00 | 4.21 | 0.00 | 5.45 | 0.00 |
| Muscle 5 | 0.00 | 0.00 | 0.00 | 0.00 | 3.57 | 0.00 | 10.32 | 0.00 | 4.43 | 0.00 |
| Ovary 1 | 0.00 | 0.00 | 10.72 | 0.00 | 1.93 | 0.00 | 16.07 | 0.00 | 5.80 | 0.00 |
| Ovary 2 | 0.00 | 0.55 | 1.79 | 0.73 | 0.71 | 0.21 | 9.67 | 0.43 | 5.75 | 0.00 |
| Ovary 3 | 0.52 | 0.88 | 5.29 | 1.59 | 1.22 | 0.00 | 21.90 | 0.58 | 6.66 | 0.00 |
| Ovary 4 | 0.66 | 0.27 | 6.05 | 1.33 | 0.71 | 0.00 | 15.08 | 0.29 | 5.56 | 0.00 |
| Ovary 5 | 1.15 | 0.88 | 12.17 | 1.35 | 1.75 | 0.39 | 28.40 | 0.66 | 5.35 | 0.00 |
| Pancreas 1 | 0.00 | 3.92 | 22.87 | 4.49 | 45.13 | 0.00 | 64.62 | 0.00 | 5.00 | 3.11 |
| Pancreas 2 | 0.00 | 0.00 | 6.21 | 1.62 | 10.79 | 0.00 | 5.19 | 0.00 | 3.25 | 0.00 |
| Pancreas 3 | 0.00 | 1.58 | 10.81 | 1.99 | 19.78 | 0.00 | 48.34 | 0.00 | 4.80 | 0.00 |
| Pancreas 4 | 0.00 | 1.97 | 12.78 | 2.28 | 22.76 | 0.00 | 44.97 | 0.00 | 2.95 | 0.00 |
| Pancreas 5 | 0.00 | 1.60 | 5.65 | 3.00 | 15.26 | 0.00 | 37.32 | 0.00 | 4.67 | 0.00 |
| Skin 1 | 0.00 | 0.00 | 9.04 | 3.18 | 2.17 | 0.00 | 5.73 | 0.00 | 4.42 | 0.00 |
| Skin 2 | 0.00 | 1.09 | 32.37 | 4.50 | 7.99 | 0.00 | 11.81 | 0.00 | 4.34 | 0.00 |
| Skin 3 | 1.24 | 2.63 | 10.93 | 9.32 | 10.19 | 0.94 | 18.82 | 0.00 | 6.01 | 0.00 |
| Skin 4 | 1.08 | 6.21 | 21.39 | 37.13 | 17.67 | 1.88 | 19.25 | 0.00 | 7.14 | 1.71 |
| Skin 5 | 1.21 | 4.24 | 20.79 | 24.21 | 17.00 | 1.53 | 25.18 | 0.00 | 7.43 | 0.00 |
| Spleen 1 | 2.15 | 0.00 | 0.97 | 0.00 | 1.09 | 0.00 | 10.01 | 1.67 | 6.86 | 0.00 |
| Spleen 2 | 4.58 | 0.92 | 1.01 | 0.78 | 1.29 | 0.00 | 20.89 | 2.82 | 11.79 | 0.00 |
| Spleen 3 | 1.84 | 0.00 | 0.00 | 1.08 | 0.93 | 0.00 | 8.84 | 1.22 | 8.94 | 0.00 |
| Spleen 4 | 2.39 | 0.00 | 0.00 | 1.61 | 0.00 | 0.00 | 20.05 | 1.46 | 9.97 | 0.00 |
| Spleen 5 | 3.14 | 0.93 | 0.97 | 0.00 | 0.00 | 0.00 | 15.29 | 2.73 | 10.89 | 0.00 |
| Stomach 1 | 0.00 | 1.46 | 6.48 | 0.00 | 8.51 | 2.30 | 17.05 | 0.00 | 2.88 | 0.00 |
| Stomach 2 | 0.00 | 0.43 | 1.67 | 0.00 | 2.73 | 0.46 | 12.50 | 0.00 | 2.13 | 0.00 |
| Stomach 3 | 4.49 | 0.00 | 9.92 | 6.75 | 3.97 | 0.00 | 0.00 | 0.00 | 41.75 | 0.00 |
| Stomach 4 | 0.00 | 0.57 | 2.87 | 2.33 | 4.92 | 1.28 | 16.32 | 0.00 | 2.02 | 0.00 |
| Stomach 5 | 0.00 | 0.41 | 1.14 | 0.00 | 1.94 | 0.59 | 9.69 | 0.00 | 1.78 | 0.00 |
| Testis 1 | 0.00 | 1.09 | 11.10 | 1.66 | 114.55 | 0.00 | 54.97 | 2.08 | 3.64 | 0.00 |
| Testis 2 | 0.00 | 0.94 | 9.36 | 1.30 | 100.88 | 1.15 | 35.36 | 1.51 | 3.96 | 0.00 |
| Testis 3 | 0.00 | 0.80 | 8.89 | 1.26 | 79.14 | 1.14 | 39.70 | 1.57 | 4.98 | 0.00 |
| Testis 4 | 0.00 | 1.01 | 9.60 | 1.31 | 82.03 | 1.17 | 49.49 | 1.45 | 4.40 | 0.00 |
| Testis 5 | 0.00 | 0.76 | 7.32 | 0.99 | 61.83 | 0.80 | 34.44 | 1.29 | 5.02 | 0.00 |
| Thyroid 1 | 0.00 | 0.00 | 2.46 | 0.00 | 0.00 | 0.00 | 192.13 | 16.14 | 8.39 | 0.00 |
| Thyroid 2 | 0.00 | 0.00 | 1.95 | 0.00 | 0.00 | 0.00 | 111.27 | 11.69 | 6.60 | 0.00 |
| Thyroid 3 | 0.00 | 0.00 | 2.03 | 0.00 | 0.00 | 0.00 | 112.65 | 13.91 | 6.68 | 0.00 |
| Thyroid 4 | 0.00 | 0.00 | 2.68 | 0.00 | 0.00 | 0.00 | 218.30 | 16.92 | 6.80 | 0.00 |
| Thyroid 5 | 0.00 | 0.00 | 2.70 | 0.00 | 0.00 | 0.00 | 158.18 | 18.74 | 6.60 | 0.00 |
| Uterus 1 | 0.68 | 0.90 | 2.18 | 1.54 | 0.88 | 0.00 | 13.57 | 0.00 | 3.01 | 0.00 |
| Uterus 2 | 0.88 | 2.74 | 5.34 | 4.73 | 2.91 | 0.00 | 37.65 | 1.81 | 8.89 | 0.00 |
| Uterus 3 | 0.00 | 2.84 | 7.80 | 7.45 | 3.21 | 1.23 | 35.04 | 0.00 | 7.09 | 0.00 |
| Uterus 4 | 0.80 | 2.59 | 11.40 | 4.24 | 3.80 | 0.97 | 41.61 | 0.00 | 9.38 | 0.00 |
| Uterus 5 | 0.65 | 1.83 | 8.99 | 3.05 | 3.16 | 0.77 | 28.88 | 1.00 | 6.43 | 0.00 |
| Serum 1 | 2.03 | 0.00 | 75.52 | 0.00 | 2.43 | 0.00 | 1.83 | 0.00 | 5.59 | 0.00 |
| Serum 2 | 2.17 | 0.00 | 36.91 | 0.00 | 1.09 | 0.00 | 1.21 | 0.00 | 4.45 | 0.00 |
| Serum 3 | 2.07 | 0.00 | 52.92 | 0.00 | 1.84 | 0.00 | 1.28 | 0.00 | 4.02 | 0.00 |
| Serum 4 | 2.57 | 0.00 | 55.56 | 0.00 | 1.55 | 0.00 | 1.78 | 0.00 | 4.31 | 0.00 |
| Serum 5 | 2.34 | 0.00 | 54.10 | 0.00 | 1.31 | 0.00 | 1.19 | 0.00 | 4.19 | 0.00 |
| Exosome 1 | 1.55 | 0.00 | 5.94 | 1.43 | 0.00 | 0.00 | 1.73 | 0.00 | 19.58 | 0.00 |
| Exosome 2 | 1.28 | 0.00 | 10.07 | 1.66 | 0.00 | 0.00 | 1.19 | 0.00 | 17.36 | 0.00 |
| Exosome 3 | 1.67 | 0.00 | 5.84 | 1.29 | 0.00 | 0.00 | 2.04 | 0.00 | 20.19 | 0.00 |
| Exosome 4 | 2.15 | 0.00 | 21.48 | 3.64 | 0.00 | 0.00 | 3.74 | 0.00 | 34.44 | 0.00 |
| Exosome 5 | 1.76 | 0.00 | 14.70 | 1.86 | 0.00 | 0.00 | 2.32 | 0.00 | 26.49 | 0.00 |

| Glycoform | **31** | **32** | **33** | **34** | **35** | **36** | **37** | **38** | **39** | **40** |
| --- | --- | --- | --- | --- | --- | --- | --- | --- | --- | --- |
| *m/z* | 2185.84 | 2188.84 | 2200.87 | 2213.87 | 2216.87 | 2229.86 | 2232.86 | 2241.9 | 2257.9 | 2273.89 |
| Abbreviation | H3N3G2 | H5N3G1 | H4N4D2 | H4N4A1 | H5N4D1 | H4N4G1 | H6N4 | H3N5D2 | H4N5D1 | H5N5 |
| Brain 1 | 0.00 | 0.00 | 12.37 | 1.12 | 11.34 | 0.00 | 0.00 | 0.00 | 5.18 | 0.00 |
| Brain 2 | 0.00 | 0.00 | 1.78 | 0.13 | 2.25 | 0.00 | 0.00 | 0.00 | 0.54 | 0.00 |
| Brain 3 | 0.00 | 0.00 | 1.00 | 0.00 | 1.31 | 0.00 | 0.00 | 0.00 | 0.33 | 0.00 |
| Brain 4 | 0.00 | 0.00 | 1.19 | 0.00 | 1.31 | 0.00 | 0.00 | 0.00 | 0.31 | 0.00 |
| Brain 5 | 0.00 | 0.00 | 1.31 | 0.00 | 1.70 | 0.00 | 0.00 | 0.00 | 0.41 | 0.00 |
| Femur 1 | 0.00 | 0.00 | 0.00 | 0.00 | 2.02 | 0.00 | 0.00 | 0.00 | 0.00 | 0.00 |
| Femur 2 | 0.00 | 0.00 | 0.00 | 0.00 | 5.52 | 0.00 | 0.00 | 0.00 | 1.79 | 0.00 |
| Femur 3 | 0.00 | 0.00 | 0.00 | 0.00 | 3.85 | 0.00 | 0.00 | 0.00 | 0.83 | 0.00 |
| Femur 4 | 0.00 | 0.00 | 0.00 | 0.00 | 2.83 | 0.00 | 0.00 | 0.00 | 0.00 | 0.00 |
| Femur 5 | 0.00 | 0.00 | 0.00 | 0.00 | 9.85 | 0.00 | 0.00 | 0.00 | 3.36 | 0.00 |
| Heart 1 | 0.00 | 0.00 | 0.00 | 0.00 | 3.19 | 0.00 | 0.00 | 0.00 | 0.00 | 0.00 |
| Heart 2 | 0.00 | 0.00 | 0.00 | 0.00 | 0.70 | 0.00 | 0.00 | 0.00 | 0.00 | 0.00 |
| Heart 3 | 0.00 | 0.00 | 0.00 | 0.00 | 0.98 | 0.00 | 0.00 | 0.00 | 0.00 | 0.00 |
| Heart 4 | 0.00 | 0.00 | 0.00 | 0.00 | 0.84 | 0.00 | 0.00 | 0.00 | 0.00 | 0.00 |
| Heart 5 | 0.00 | 0.00 | 0.00 | 0.00 | 2.71 | 0.00 | 0.00 | 0.00 | 0.00 | 0.00 |
| Intestines 1 | 0.00 | 0.00 | 0.00 | 0.00 | 14.38 | 2.85 | 5.29 | 2.65 | 82.52 | 0.00 |
| Intestines 2 | 0.00 | 0.56 | 0.00 | 0.00 | 4.37 | 0.60 | 2.08 | 0.00 | 21.57 | 2.07 |
| Intestines 3 | 0.00 | 0.92 | 0.00 | 0.00 | 4.24 | 0.00 | 2.87 | 0.00 | 17.22 | 3.93 |
| Intestines 4 | 0.00 | 1.72 | 0.00 | 0.72 | 6.08 | 0.98 | 2.86 | 0.00 | 12.28 | 3.23 |
| Intestines 5 | 0.00 | 0.00 | 0.00 | 0.00 | 4.97 | 0.00 | 1.97 | 0.55 | 16.08 | 3.19 |
| Kidney 1 | 0.00 | 0.00 | 0.00 | 0.00 | 4.59 | 0.00 | 0.00 | 0.00 | 12.43 | 0.00 |
| Kidney 2 | 0.00 | 0.00 | 0.73 | 0.00 | 2.91 | 0.00 | 0.00 | 0.00 | 7.39 | 0.00 |
| Kidney 3 | 0.00 | 0.00 | 0.00 | 0.00 | 3.17 | 0.00 | 0.00 | 0.00 | 7.58 | 0.00 |
| Kidney 4 | 0.00 | 0.00 | 0.00 | 0.00 | 1.31 | 0.00 | 0.00 | 0.00 | 2.89 | 0.00 |
| Kidney 5 | 0.00 | 0.00 | 0.56 | 0.00 | 2.53 | 0.00 | 0.00 | 0.00 | 7.32 | 0.00 |
| Liver 1 | 0.00 | 1.17 | 0.00 | 0.00 | 0.00 | 0.79 | 0.00 | 0.00 | 0.00 | 0.00 |
| Liver 2 | 0.00 | 0.00 | 0.00 | 0.00 | 0.00 | 0.00 | 0.00 | 0.00 | 0.00 | 0.00 |
| Liver 3 | 0.05 | 0.00 | 0.00 | 0.00 | 0.56 | 0.14 | 0.00 | 0.00 | 0.00 | 0.00 |
| Liver 4 | 0.04 | 0.00 | 0.00 | 0.00 | 0.43 | 0.09 | 0.00 | 0.00 | 0.00 | 0.00 |
| Liver 5 | 0.00 | 0.06 | 0.00 | 0.00 | 0.00 | 0.06 | 0.00 | 0.00 | 0.00 | 0.00 |
| Lung 1 | 0.00 | 1.60 | 0.00 | 0.00 | 21.89 | 0.00 | 0.00 | 0.00 | 0.87 | 0.00 |
| Lung 2 | 0.00 | 1.61 | 0.00 | 0.00 | 14.07 | 0.00 | 0.00 | 0.00 | 0.97 | 0.00 |
| Lung 3 | 0.00 | 2.07 | 0.00 | 0.00 | 52.17 | 0.00 | 0.00 | 0.00 | 2.59 | 0.00 |
| Lung 4 | 0.00 | 0.70 | 0.00 | 0.00 | 14.39 | 0.00 | 0.00 | 0.00 | 0.00 | 0.00 |
| Lung 5 | 0.00 | 1.66 | 0.00 | 0.00 | 27.91 | 0.00 | 0.00 | 0.00 | 1.64 | 0.00 |
| Muscle 1 | 0.00 | 0.00 | 0.00 | 0.00 | 4.12 | 0.00 | 0.00 | 0.00 | 0.00 | 0.00 |
| Muscle 2 | 0.00 | 0.00 | 0.00 | 0.00 | 3.06 | 0.00 | 0.00 | 0.00 | 0.00 | 0.00 |
| Muscle 3 | 0.00 | 0.00 | 0.00 | 0.00 | 3.57 | 0.00 | 0.00 | 0.00 | 0.00 | 0.00 |
| Muscle 4 | 0.00 | 0.00 | 0.00 | 0.00 | 1.52 | 0.00 | 0.00 | 0.00 | 0.00 | 0.00 |
| Muscle 5 | 0.00 | 0.00 | 0.00 | 0.00 | 4.38 | 0.00 | 0.00 | 0.00 | 0.00 | 0.00 |
| Ovary 1 | 0.00 | 1.79 | 0.00 | 0.00 | 7.51 | 0.00 | 0.00 | 0.00 | 0.00 | 0.00 |
| Ovary 2 | 0.00 | 0.41 | 0.00 | 0.00 | 8.25 | 0.00 | 0.00 | 0.00 | 0.83 | 0.00 |
| Ovary 3 | 0.00 | 0.69 | 0.00 | 0.00 | 0.00 | 0.00 | 0.00 | 0.00 | 1.78 | 0.00 |
| Ovary 4 | 0.00 | 0.66 | 0.00 | 0.00 | 4.98 | 0.00 | 0.00 | 0.00 | 0.91 | 0.00 |
| Ovary 5 | 0.00 | 1.02 | 0.00 | 0.00 | 8.57 | 0.00 | 0.00 | 0.00 | 1.71 | 0.00 |
| Pancreas 1 | 0.00 | 0.00 | 4.83 | 0.00 | 54.61 | 0.00 | 0.00 | 0.00 | 3.13 | 0.00 |
| Pancreas 2 | 0.00 | 0.00 | 0.00 | 0.00 | 5.58 | 0.00 | 0.00 | 0.00 | 1.53 | 0.00 |
| Pancreas 3 | 0.00 | 0.00 | 1.25 | 0.00 | 15.09 | 0.00 | 0.00 | 0.00 | 1.40 | 0.00 |
| Pancreas 4 | 0.00 | 0.00 | 1.73 | 0.00 | 19.30 | 0.00 | 0.00 | 0.00 | 2.08 | 0.00 |
| Pancreas 5 | 0.00 | 0.00 | 0.00 | 0.00 | 28.63 | 0.00 | 0.00 | 0.00 | 0.00 | 0.00 |
| Skin 1 | 0.00 | 1.02 | 0.00 | 0.00 | 13.72 | 0.00 | 0.00 | 0.00 | 1.19 | 0.00 |
| Skin 2 | 0.00 | 1.28 | 0.00 | 0.00 | 22.93 | 0.00 | 0.00 | 0.00 | 2.97 | 0.00 |
| Skin 3 | 0.00 | 1.18 | 0.00 | 0.00 | 39.98 | 0.00 | 0.00 | 0.00 | 3.46 | 1.05 |
| Skin 4 | 0.00 | 1.58 | 0.00 | 0.00 | 88.28 | 0.00 | 0.00 | 0.00 | 6.65 | 1.17 |
| Skin 5 | 0.00 | 1.34 | 0.00 | 0.00 | 72.67 | 0.00 | 0.00 | 0.00 | 6.67 | 0.00 |
| Spleen 1 | 0.00 | 0.00 | 0.00 | 0.00 | 3.59 | 0.00 | 0.00 | 0.00 | 1.24 | 0.00 |
| Spleen 2 | 0.00 | 2.19 | 0.00 | 0.00 | 2.97 | 0.00 | 0.00 | 0.00 | 1.21 | 0.00 |
| Spleen 3 | 0.00 | 1.26 | 0.00 | 0.00 | 2.32 | 0.00 | 0.00 | 0.00 | 0.00 | 0.00 |
| Spleen 4 | 0.00 | 2.03 | 0.00 | 0.00 | 1.97 | 0.00 | 0.00 | 0.00 | 0.00 | 0.00 |
| Spleen 5 | 0.00 | 1.70 | 0.00 | 0.00 | 2.79 | 0.00 | 0.00 | 0.00 | 0.99 | 0.00 |
| Stomach 1 | 0.00 | 0.00 | 0.00 | 0.00 | 12.86 | 0.00 | 0.00 | 0.00 | 8.33 | 0.00 |
| Stomach 2 | 0.00 | 0.00 | 0.39 | 0.00 | 3.59 | 0.00 | 0.00 | 0.45 | 2.30 | 0.00 |
| Stomach 3 | 0.00 | 0.00 | 1.77 | 0.89 | 3.51 | 2.95 | 0.86 | 1.54 | 7.18 | 1.75 |
| Stomach 4 | 0.00 | 0.00 | 0.55 | 0.00 | 4.45 | 0.00 | 0.00 | 0.48 | 4.63 | 0.41 |
| Stomach 5 | 0.00 | 0.00 | 0.26 | 0.00 | 2.53 | 0.00 | 0.00 | 0.39 | 1.90 | 0.22 |
| Testis 1 | 0.00 | 0.00 | 0.00 | 0.00 | 17.46 | 0.00 | 0.00 | 0.00 | 6.01 | 0.00 |
| Testis 2 | 0.00 | 0.00 | 0.00 | 0.00 | 10.66 | 0.00 | 0.00 | 0.00 | 4.81 | 0.00 |
| Testis 3 | 0.00 | 0.00 | 0.00 | 0.00 | 10.62 | 0.00 | 0.00 | 0.00 | 4.56 | 0.00 |
| Testis 4 | 0.00 | 0.00 | 0.00 | 0.90 | 14.88 | 0.00 | 0.00 | 0.00 | 5.68 | 0.00 |
| Testis 5 | 0.00 | 0.00 | 0.00 | 0.64 | 10.88 | 0.00 | 0.00 | 0.00 | 3.98 | 0.00 |
| Thyroid 1 | 0.00 | 0.00 | 0.00 | 0.00 | 59.46 | 0.00 | 0.00 | 0.00 | 0.00 | 0.00 |
| Thyroid 2 | 0.00 | 0.00 | 0.00 | 0.00 | 36.60 | 0.00 | 0.00 | 0.00 | 0.00 | 0.00 |
| Thyroid 3 | 0.00 | 0.00 | 0.00 | 0.00 | 33.89 | 0.00 | 0.00 | 0.00 | 0.00 | 0.00 |
| Thyroid 4 | 0.00 | 0.00 | 0.00 | 0.00 | 62.12 | 0.00 | 0.00 | 0.00 | 0.00 | 0.00 |
| Thyroid 5 | 0.00 | 0.00 | 0.00 | 0.00 | 41.85 | 0.00 | 0.00 | 0.00 | 0.00 | 0.00 |
| Uterus 1 | 0.00 | 0.83 | 0.00 | 0.00 | 5.15 | 0.00 | 0.00 | 0.00 | 0.90 | 0.00 |
| Uterus 2 | 0.00 | 1.06 | 0.00 | 0.00 | 23.05 | 0.00 | 0.00 | 0.00 | 2.85 | 0.00 |
| Uterus 3 | 0.00 | 0.00 | 0.00 | 0.00 | 22.87 | 0.00 | 0.00 | 0.00 | 3.35 | 0.00 |
| Uterus 4 | 0.00 | 1.04 | 0.65 | 0.00 | 24.21 | 0.00 | 0.00 | 0.00 | 2.85 | 0.00 |
| Uterus 5 | 0.00 | 0.73 | 0.56 | 0.00 | 15.29 | 0.00 | 0.00 | 0.00 | 2.24 | 0.00 |
| Serum 1 | 0.00 | 5.54 | 0.00 | 0.00 | 13.21 | 2.93 | 0.00 | 0.00 | 0.00 | 0.00 |
| Serum 2 | 0.00 | 5.45 | 0.00 | 0.00 | 4.51 | 2.32 | 0.00 | 0.00 | 0.00 | 0.00 |
| Serum 3 | 0.00 | 5.41 | 0.00 | 0.00 | 5.75 | 2.36 | 0.00 | 0.00 | 0.00 | 0.00 |
| Serum 4 | 0.00 | 6.51 | 0.00 | 0.00 | 6.00 | 2.88 | 0.00 | 0.00 | 0.00 | 0.00 |
| Serum 5 | 0.00 | 5.32 | 0.00 | 0.00 | 5.19 | 2.17 | 0.00 | 0.00 | 0.00 | 0.00 |
| Exosome 1 | 0.00 | 2.04 | 0.00 | 0.00 | 7.80 | 1.98 | 0.00 | 0.00 | 0.00 | 0.00 |
| Exosome 2 | 0.60 | 1.78 | 0.00 | 0.00 | 5.68 | 1.74 | 0.00 | 0.00 | 0.00 | 0.00 |
| Exosome 3 | 0.00 | 0.00 | 0.00 | 0.00 | 6.86 | 2.01 | 0.00 | 0.00 | 0.00 | 0.00 |
| Exosome 4 | 0.00 | 2.30 | 0.00 | 0.00 | 18.10 | 2.33 | 0.00 | 0.00 | 0.00 | 0.00 |
| Exosome 5 | 0.00 | 0.00 | 0.00 | 0.00 | 17.51 | 2.05 | 0.00 | 0.00 | 0.00 | 0.00 |

| Glycoform | **41** | **42** | **43** | **44** | **45** | **46** | **47** | **48** | **49** | **50** |
| --- | --- | --- | --- | --- | --- | --- | --- | --- | --- | --- |
| *m/z* | 2298.92 | 2312.86 | 2318.9 | 2334.9 | 2350.89 | 2359.93 | 2362.93 | 2375.92 | 2378.92 | 2391.92 |
| Abbreviation | H3N6D1 | H9N2 | H5N3D1A1 | H6N3A1 | H6N3G1 | H4N4D1A1 | H5N4D2 | H5N4A1 | H6N4D1 | H5N4G1 |
| Brain 1 | 1.33 | 6.08 | 0.00 | 0.00 | 0.00 | 1.42 | 4.57 | 0.00 | 0.00 | 0.00 |
| Brain 2 | 0.11 | 1.67 | 0.00 | 0.00 | 0.00 | 0.18 | 0.40 | 0.00 | 0.00 | 0.00 |
| Brain 3 | 0.00 | 0.94 | 0.00 | 0.00 | 0.00 | 0.13 | 0.20 | 0.00 | 0.00 | 0.00 |
| Brain 4 | 0.00 | 0.54 | 0.00 | 0.00 | 0.00 | 0.00 | 0.26 | 0.00 | 0.00 | 0.00 |
| Brain 5 | 0.10 | 1.14 | 0.00 | 0.00 | 0.00 | 0.15 | 0.30 | 0.00 | 0.00 | 0.00 |
| Femur 1 | 0.00 | 9.66 | 0.00 | 0.00 | 0.00 | 0.00 | 0.00 | 0.00 | 0.00 | 0.00 |
| Femur 2 | 2.01 | 14.01 | 0.00 | 1.62 | 0.00 | 0.00 | 0.00 | 1.21 | 0.00 | 2.23 |
| Femur 3 | 0.00 | 14.32 | 0.00 | 0.85 | 0.00 | 0.00 | 0.00 | 0.00 | 0.00 | 0.00 |
| Femur 4 | 0.00 | 8.07 | 0.00 | 1.06 | 0.00 | 0.00 | 0.00 | 0.00 | 0.00 | 1.60 |
| Femur 5 | 2.74 | 24.34 | 0.00 | 0.00 | 0.00 | 0.00 | 0.00 | 0.00 | 0.00 | 0.00 |
| Heart 1 | 0.00 | 2.92 | 0.00 | 1.13 | 0.00 | 0.00 | 0.00 | 0.00 | 0.00 | 0.95 |
| Heart 2 | 0.00 | 2.21 | 0.00 | 0.28 | 0.00 | 0.00 | 0.00 | 0.00 | 0.00 | 0.31 |
| Heart 3 | 0.00 | 4.20 | 0.00 | 0.61 | 0.00 | 0.00 | 0.00 | 0.00 | 0.00 | 0.50 |
| Heart 4 | 0.00 | 4.39 | 0.00 | 0.43 | 0.13 | 0.00 | 0.00 | 0.00 | 0.00 | 0.21 |
| Heart 5 | 0.00 | 3.18 | 0.00 | 0.39 | 0.00 | 0.00 | 0.00 | 0.00 | 0.00 | 0.36 |
| Intestines 1 | 3.19 | 0.00 | 0.00 | 0.00 | 0.00 | 0.00 | 0.00 | 0.00 | 0.00 | 2.59 |
| Intestines 2 | 1.08 | 15.64 | 0.00 | 0.00 | 0.00 | 0.00 | 0.00 | 0.00 | 0.57 | 0.76 |
| Intestines 3 | 1.85 | 15.15 | 0.00 | 0.00 | 0.00 | 0.00 | 0.00 | 0.00 | 0.00 | 1.15 |
| Intestines 4 | 1.03 | 11.07 | 0.00 | 0.00 | 0.00 | 0.00 | 0.00 | 0.00 | 0.52 | 1.89 |
| Intestines 5 | 1.52 | 13.18 | 0.00 | 0.00 | 0.00 | 0.00 | 0.00 | 0.00 | 0.00 | 0.00 |
| Kidney 1 | 0.00 | 14.77 | 0.00 | 0.00 | 0.00 | 0.00 | 1.05 | 0.00 | 0.00 | 0.00 |
| Kidney 2 | 0.00 | 15.43 | 0.00 | 0.00 | 0.00 | 0.00 | 0.68 | 0.00 | 0.00 | 1.21 |
| Kidney 3 | 0.00 | 17.45 | 0.00 | 0.00 | 0.00 | 0.00 | 0.00 | 0.00 | 0.00 | 0.00 |
| Kidney 4 | 0.00 | 10.47 | 0.00 | 0.00 | 0.00 | 0.00 | 0.00 | 0.00 | 0.00 | 0.85 |
| Kidney 5 | 0.00 | 20.92 | 0.00 | 0.00 | 0.00 | 0.00 | 0.80 | 0.00 | 0.00 | 1.04 |
| Liver 1 | 0.00 | 24.59 | 0.00 | 0.00 | 0.94 | 0.00 | 0.00 | 0.00 | 0.00 | 1.88 |
| Liver 2 | 0.00 | 1.02 | 0.00 | 0.00 | 0.00 | 0.00 | 0.00 | 0.00 | 0.00 | 0.00 |
| Liver 3 | 0.00 | 2.37 | 0.00 | 0.00 | 0.09 | 0.00 | 0.00 | 0.00 | 0.00 | 0.22 |
| Liver 4 | 0.00 | 1.80 | 0.00 | 0.00 | 0.09 | 0.00 | 0.00 | 0.00 | 0.00 | 0.13 |
| Liver 5 | 0.00 | 0.91 | 0.00 | 0.00 | 0.05 | 0.00 | 0.00 | 0.00 | 0.00 | 0.11 |
| Lung 1 | 0.00 | 18.52 | 0.00 | 1.39 | 1.30 | 0.00 | 0.00 | 1.27 | 0.73 | 4.42 |
| Lung 2 | 0.00 | 13.23 | 0.00 | 2.94 | 1.40 | 1.00 | 0.00 | 2.65 | 0.00 | 4.34 |
| Lung 3 | 0.00 | 27.14 | 0.00 | 2.22 | 1.56 | 0.00 | 0.00 | 3.45 | 1.07 | 7.77 |
| Lung 4 | 0.00 | 3.74 | 0.00 | 0.00 | 0.00 | 0.00 | 0.00 | 0.88 | 0.00 | 2.90 |
| Lung 5 | 0.00 | 8.70 | 0.79 | 1.23 | 1.03 | 0.00 | 0.00 | 1.75 | 0.00 | 4.42 |
| Muscle 1 | 0.00 | 4.73 | 0.00 | 0.00 | 0.00 | 0.00 | 0.00 | 0.00 | 0.00 | 0.00 |
| Muscle 2 | 0.00 | 3.55 | 0.00 | 0.00 | 0.00 | 0.00 | 0.00 | 0.00 | 0.00 | 0.00 |
| Muscle 3 | 0.00 | 5.69 | 0.00 | 0.00 | 0.00 | 0.00 | 0.00 | 0.00 | 0.00 | 0.00 |
| Muscle 4 | 0.00 | 2.63 | 0.00 | 0.00 | 0.00 | 0.00 | 0.00 | 0.00 | 0.00 | 0.00 |
| Muscle 5 | 0.00 | 8.72 | 1.21 | 0.00 | 0.00 | 0.00 | 0.00 | 0.00 | 0.00 | 3.18 |
| Ovary 1 | 0.00 | 10.47 | 0.00 | 0.00 | 0.00 | 0.00 | 0.00 | 2.25 | 0.00 | 3.05 |
| Ovary 2 | 0.00 | 5.51 | 0.00 | 0.41 | 0.26 | 0.26 | 0.00 | 1.03 | 0.00 | 0.85 |
| Ovary 3 | 0.00 | 11.59 | 0.00 | 0.00 | 0.00 | 0.00 | 0.00 | 1.46 | 0.49 | 1.81 |
| Ovary 4 | 0.00 | 10.65 | 0.00 | 0.31 | 0.40 | 0.28 | 0.00 | 1.37 | 0.00 | 1.93 |
| Ovary 5 | 0.00 | 15.97 | 0.00 | 0.47 | 0.74 | 0.33 | 0.00 | 2.07 | 0.38 | 2.37 |
| Pancreas 1 | 30.20 | 55.77 | 0.00 | 0.00 | 0.00 | 0.00 | 7.49 | 0.00 | 13.27 | 0.00 |
| Pancreas 2 | 4.61 | 1.50 | 0.00 | 0.00 | 0.00 | 0.00 | 0.00 | 0.00 | 0.00 | 0.00 |
| Pancreas 3 | 11.35 | 42.87 | 0.00 | 0.00 | 0.00 | 0.00 | 1.68 | 0.00 | 4.03 | 0.00 |
| Pancreas 4 | 14.08 | 43.25 | 0.00 | 0.00 | 0.00 | 0.00 | 2.80 | 0.00 | 5.58 | 0.00 |
| Pancreas 5 | 8.99 | 36.37 | 0.00 | 0.00 | 0.00 | 0.00 | 8.76 | 0.00 | 2.49 | 0.00 |
| Skin 1 | 0.00 | 3.36 | 0.00 | 0.00 | 0.00 | 0.00 | 0.00 | 1.73 | 0.72 | 8.18 |
| Skin 2 | 0.00 | 6.78 | 0.00 | 0.00 | 0.63 | 0.00 | 0.00 | 3.31 | 0.79 | 14.84 |
| Skin 3 | 0.84 | 9.69 | 1.01 | 0.00 | 0.00 | 0.00 | 0.00 | 0.00 | 0.00 | 19.39 |
| Skin 4 | 0.93 | 9.80 | 1.14 | 0.00 | 0.00 | 0.00 | 0.00 | 0.00 | 0.00 | 38.51 |
| Skin 5 | 0.00 | 13.99 | 0.00 | 0.00 | 0.00 | 0.00 | 0.00 | 0.00 | 0.00 | 24.52 |
| Spleen 1 | 0.00 | 7.84 | 0.00 | 0.00 | 0.00 | 0.00 | 0.00 | 0.00 | 0.00 | 0.98 |
| Spleen 2 | 0.00 | 24.05 | 0.00 | 1.15 | 1.98 | 0.00 | 0.00 | 1.38 | 0.00 | 2.68 |
| Spleen 3 | 0.00 | 7.84 | 0.00 | 0.89 | 0.89 | 0.00 | 0.00 | 0.94 | 0.00 | 1.42 |
| Spleen 4 | 0.00 | 18.00 | 0.00 | 1.63 | 1.64 | 0.00 | 0.00 | 0.86 | 0.00 | 1.37 |
| Spleen 5 | 0.00 | 17.68 | 0.00 | 0.00 | 1.62 | 0.00 | 0.00 | 1.60 | 0.00 | 2.44 |
| Stomach 1 | 2.26 | 9.41 | 0.00 | 0.00 | 0.00 | 0.00 | 0.00 | 0.00 | 0.00 | 2.59 |
| Stomach 2 | 0.93 | 6.82 | 0.00 | 0.00 | 0.00 | 0.00 | 0.41 | 0.42 | 0.00 | 0.00 |
| Stomach 3 | 0.53 | 0.00 | 0.00 | 0.00 | 0.00 | 0.00 | 0.00 | 0.00 | 0.00 | 0.72 |
| Stomach 4 | 1.78 | 9.46 | 0.00 | 0.00 | 0.00 | 0.00 | 0.00 | 0.46 | 0.00 | 2.03 |
| Stomach 5 | 0.66 | 4.75 | 0.00 | 0.00 | 0.00 | 0.00 | 0.31 | 0.33 | 0.00 | 0.76 |
| Testis 1 | 7.73 | 22.36 | 0.00 | 1.85 | 0.00 | 0.00 | 0.00 | 0.00 | 0.00 | 0.81 |
| Testis 2 | 7.35 | 10.38 | 0.00 | 2.07 | 0.00 | 0.64 | 0.00 | 0.00 | 0.00 | 0.00 |
| Testis 3 | 6.38 | 14.77 | 0.00 | 1.64 | 0.00 | 0.60 | 0.00 | 0.00 | 0.00 | 0.43 |
| Testis 4 | 6.81 | 19.11 | 0.00 | 2.42 | 0.00 | 0.72 | 0.00 | 0.00 | 0.00 | 0.47 |
| Testis 5 | 4.50 | 11.81 | 0.00 | 2.37 | 0.00 | 0.56 | 0.00 | 0.00 | 0.00 | 0.46 |
| Thyroid 1 | 0.00 | 265.35 | 0.00 | 0.00 | 0.00 | 6.77 | 0.00 | 1.85 | 0.00 | 0.00 |
| Thyroid 2 | 0.00 | 147.50 | 2.28 | 2.04 | 0.00 | 4.88 | 0.00 | 1.23 | 0.00 | 0.00 |
| Thyroid 3 | 0.00 | 159.41 | 2.10 | 1.99 | 0.00 | 5.63 | 0.00 | 1.21 | 0.00 | 0.00 |
| Thyroid 4 | 2.54 | 331.59 | 4.89 | 2.85 | 0.00 | 6.84 | 0.00 | 1.64 | 0.00 | 0.00 |
| Thyroid 5 | 2.39 | 226.12 | 3.95 | 3.01 | 0.00 | 7.33 | 0.00 | 1.48 | 0.00 | 0.00 |
| Uterus 1 | 0.00 | 6.88 | 0.00 | 0.00 | 0.00 | 0.00 | 0.00 | 1.35 | 0.00 | 1.96 |
| Uterus 2 | 0.00 | 22.57 | 0.00 | 0.00 | 0.00 | 0.00 | 0.00 | 3.08 | 1.20 | 3.08 |
| Uterus 3 | 0.00 | 16.50 | 0.00 | 0.00 | 0.00 | 0.00 | 0.00 | 3.51 | 1.39 | 2.14 |
| Uterus 4 | 0.45 | 17.17 | 0.00 | 0.63 | 0.42 | 0.00 | 0.00 | 2.13 | 0.69 | 2.40 |
| Uterus 5 | 0.00 | 11.67 | 0.00 | 0.60 | 0.00 | 0.41 | 0.00 | 1.70 | 0.47 | 1.76 |
| Serum 1 | 0.00 | 1.60 | 0.00 | 0.00 | 2.69 | 0.00 | 0.00 | 3.46 | 0.00 | 6.90 |
| Serum 2 | 0.00 | 0.94 | 0.00 | 0.00 | 2.94 | 0.00 | 0.00 | 3.14 | 0.00 | 6.09 |
| Serum 3 | 0.00 | 0.00 | 0.00 | 0.00 | 2.77 | 0.00 | 0.00 | 2.72 | 0.00 | 5.51 |
| Serum 4 | 0.00 | 1.46 | 0.00 | 0.00 | 3.56 | 0.00 | 0.00 | 3.78 | 0.00 | 7.82 |
| Serum 5 | 0.00 | 1.11 | 0.00 | 0.00 | 2.49 | 0.00 | 0.00 | 3.10 | 0.00 | 5.70 |
| Exosome 1 | 0.00 | 2.87 | 0.00 | 0.91 | 1.29 | 0.00 | 0.00 | 1.71 | 0.00 | 6.44 |
| Exosome 2 | 0.00 | 1.23 | 0.00 | 0.73 | 1.09 | 0.00 | 0.00 | 1.66 | 0.00 | 5.52 |
| Exosome 3 | 0.00 | 2.90 | 0.00 | 1.03 | 1.38 | 0.00 | 0.00 | 1.68 | 0.00 | 6.39 |
| Exosome 4 | 0.00 | 2.85 | 0.00 | 1.67 | 0.00 | 0.00 | 0.00 | 5.16 | 0.00 | 8.85 |
| Exosome 5 | 0.00 | 2.25 | 0.00 | 1.25 | 0.00 | 0.00 | 0.00 | 4.04 | 0.00 | 6.99 |

| Glycoform | **51** | **52** | **53** | **54** | **55** | **56** | **57** | **58** | **60** |
| --- | --- | --- | --- | --- | --- | --- | --- | --- | --- |
| *m/z* | 2403.95 | 2416.95 | 2419.95 | 2435.94 | 2444.98 | 2445.96 | 2460.98 | 2474.92 | 2489.99 |
| Abbreviation | H4N5D2 | H4N5A1 | H5N5D1 | H6N5 | H3N6D2 | H3N3D2A2 | H4N6D1 | H10N2 | H3N4D3A1 |
| Brain 1 | 23.41 | 0.00 | 0.00 | 1.33 | 0.00 | 0.00 | 1.76 | 0.00 | 0.00 |
| Brain 2 | 3.32 | 0.00 | 0.00 | 0.68 | 0.00 | 0.00 | 0.00 | 0.00 | 0.00 |
| Brain 3 | 1.92 | 0.00 | 0.00 | 0.63 | 0.00 | 0.00 | 0.00 | 0.00 | 0.00 |
| Brain 4 | 2.22 | 0.00 | 0.00 | 0.37 | 0.00 | 0.00 | 0.00 | 0.00 | 0.00 |
| Brain 5 | 2.55 | 0.00 | 0.00 | 0.92 | 0.00 | 0.10 | 0.00 | 0.00 | 0.00 |
| Femur 1 | 0.00 | 0.00 | 0.00 | 0.86 | 0.00 | 0.00 | 0.00 | 0.00 | 0.00 |
| Femur 2 | 0.00 | 0.00 | 1.10 | 1.60 | 0.00 | 0.00 | 0.00 | 0.00 | 0.00 |
| Femur 3 | 0.00 | 0.00 | 0.00 | 1.00 | 0.00 | 0.00 | 0.00 | 0.00 | 0.00 |
| Femur 4 | 0.00 | 0.00 | 0.00 | 1.03 | 0.00 | 0.00 | 0.00 | 0.00 | 0.00 |
| Femur 5 | 0.00 | 0.00 | 1.80 | 1.27 | 0.00 | 0.00 | 0.00 | 0.00 | 0.00 |
| Heart 1 | 0.00 | 0.00 | 0.00 | 1.62 | 0.00 | 0.00 | 0.00 | 1.82 | 0.00 |
| Heart 2 | 0.00 | 0.00 | 0.00 | 0.17 | 0.00 | 0.00 | 0.00 | 0.00 | 0.00 |
| Heart 3 | 0.00 | 0.00 | 0.00 | 0.21 | 0.00 | 0.00 | 0.00 | 0.00 | 0.00 |
| Heart 4 | 0.00 | 0.00 | 0.00 | 0.18 | 0.00 | 0.00 | 0.00 | 0.00 | 0.00 |
| Heart 5 | 0.00 | 0.00 | 0.00 | 0.00 | 0.00 | 0.00 | 0.00 | 0.00 | 0.00 |
| Intestines 1 | 3.14 | 0.00 | 11.43 | 0.00 | 0.00 | 0.00 | 17.81 | 0.00 | 0.00 |
| Intestines 2 | 1.13 | 0.00 | 6.95 | 1.28 | 0.00 | 0.60 | 0.00 | 0.00 | 0.00 |
| Intestines 3 | 0.87 | 0.00 | 8.16 | 1.76 | 0.00 | 0.96 | 1.99 | 0.00 | 0.00 |
| Intestines 4 | 1.01 | 0.58 | 4.60 | 2.00 | 0.00 | 0.00 | 2.14 | 0.00 | 0.00 |
| Intestines 5 | 0.95 | 0.00 | 13.43 | 0.00 | 0.00 | 1.11 | 1.72 | 0.00 | 0.00 |
| Kidney 1 | 12.93 | 0.00 | 2.72 | 1.52 | 0.00 | 0.00 | 0.00 | 0.00 | 0.00 |
| Kidney 2 | 7.84 | 0.00 | 0.00 | 2.04 | 0.00 | 0.00 | 0.00 | 2.07 | 0.00 |
| Kidney 3 | 9.57 | 0.00 | 0.00 | 1.80 | 0.00 | 0.00 | 0.00 | 0.00 | 0.00 |
| Kidney 4 | 4.26 | 0.00 | 0.00 | 1.26 | 0.00 | 0.00 | 0.00 | 0.00 | 0.00 |
| Kidney 5 | 8.78 | 0.00 | 0.00 | 1.68 | 0.00 | 0.00 | 0.00 | 0.00 | 0.00 |
| Liver 1 | 0.00 | 0.00 | 0.00 | 1.75 | 0.00 | 0.00 | 0.00 | 0.00 | 0.00 |
| Liver 2 | 0.00 | 0.00 | 0.00 | 0.55 | 0.00 | 0.00 | 0.00 | 0.00 | 0.00 |
| Liver 3 | 0.00 | 0.00 | 0.00 | 1.03 | 0.00 | 0.07 | 0.00 | 0.00 | 0.66 |
| Liver 4 | 0.00 | 0.00 | 0.00 | 0.89 | 0.00 | 0.07 | 0.00 | 0.00 | 0.00 |
| Liver 5 | 0.00 | 0.00 | 0.00 | 0.60 | 0.00 | 0.00 | 0.00 | 0.00 | 0.00 |
| Lung 1 | 0.00 | 0.00 | 0.82 | 2.42 | 0.00 | 0.00 | 0.00 | 0.00 | 0.00 |
| Lung 2 | 0.00 | 0.00 | 1.01 | 1.66 | 0.00 | 0.00 | 0.00 | 0.00 | 0.00 |
| Lung 3 | 0.00 | 0.00 | 2.13 | 0.00 | 0.00 | 0.00 | 0.00 | 0.00 | 0.00 |
| Lung 4 | 0.00 | 0.00 | 0.00 | 0.00 | 0.00 | 0.00 | 0.00 | 0.00 | 0.00 |
| Lung 5 | 0.00 | 0.00 | 0.83 | 1.13 | 0.00 | 0.00 | 0.00 | 0.00 | 0.00 |
| Muscle 1 | 0.00 | 0.00 | 0.00 | 1.78 | 0.00 | 0.00 | 0.00 | 0.00 | 0.00 |
| Muscle 2 | 0.00 | 0.00 | 0.00 | 1.25 | 0.00 | 0.00 | 0.00 | 0.00 | 0.00 |
| Muscle 3 | 0.00 | 0.00 | 0.00 | 1.71 | 0.00 | 0.00 | 0.00 | 0.00 | 0.00 |
| Muscle 4 | 0.00 | 0.00 | 0.00 | 0.92 | 0.00 | 0.00 | 0.00 | 0.00 | 0.00 |
| Muscle 5 | 0.00 | 0.00 | 0.00 | 1.26 | 0.00 | 0.00 | 0.00 | 0.00 | 0.00 |
| Ovary 1 | 0.00 | 0.00 | 0.00 | 3.15 | 0.00 | 0.00 | 0.00 | 0.00 | 0.00 |
| Ovary 2 | 0.00 | 0.00 | 0.59 | 0.56 | 0.00 | 0.00 | 0.00 | 0.00 | 0.00 |
| Ovary 3 | 0.00 | 0.00 | 1.36 | 0.88 | 0.00 | 0.00 | 0.00 | 0.00 | 0.00 |
| Ovary 4 | 0.00 | 0.00 | 0.69 | 0.73 | 0.00 | 0.00 | 0.00 | 0.00 | 0.00 |
| Ovary 5 | 0.00 | 0.00 | 1.27 | 0.77 | 0.00 | 0.00 | 0.00 | 0.00 | 0.00 |
| Pancreas 1 | 0.00 | 0.00 | 2.39 | 2.15 | 0.00 | 0.00 | 0.00 | 0.00 | 0.00 |
| Pancreas 2 | 0.00 | 0.00 | 0.00 | 1.04 | 0.00 | 0.00 | 0.00 | 0.00 | 0.00 |
| Pancreas 3 | 0.00 | 0.00 | 0.00 | 0.00 | 0.00 | 0.00 | 0.00 | 0.00 | 0.00 |
| Pancreas 4 | 0.00 | 0.00 | 1.65 | 0.00 | 0.00 | 0.00 | 0.00 | 0.00 | 0.00 |
| Pancreas 5 | 0.00 | 0.00 | 0.00 | 0.00 | 0.00 | 0.00 | 0.00 | 0.00 | 0.00 |
| Skin 1 | 0.00 | 0.00 | 0.86 | 2.28 | 0.00 | 0.00 | 0.00 | 0.00 | 0.00 |
| Skin 2 | 0.00 | 0.00 | 0.00 | 1.33 | 0.00 | 0.00 | 0.00 | 0.00 | 0.00 |
| Skin 3 | 0.00 | 0.00 | 2.62 | 2.49 | 0.00 | 0.00 | 0.00 | 0.00 | 0.00 |
| Skin 4 | 0.00 | 1.24 | 4.13 | 4.55 | 0.00 | 0.00 | 0.00 | 0.00 | 0.00 |
| Skin 5 | 0.00 | 1.31 | 4.02 | 3.01 | 0.00 | 0.00 | 0.00 | 0.00 | 0.00 |
| Spleen 1 | 0.00 | 0.00 | 1.44 | 2.05 | 0.00 | 0.00 | 0.00 | 0.00 | 0.00 |
| Spleen 2 | 0.00 | 0.00 | 1.58 | 1.53 | 0.00 | 0.00 | 0.00 | 0.00 | 0.00 |
| Spleen 3 | 0.00 | 0.00 | 0.00 | 1.56 | 0.00 | 0.00 | 0.00 | 0.00 | 0.00 |
| Spleen 4 | 0.00 | 0.00 | 0.79 | 1.58 | 0.00 | 0.00 | 0.00 | 2.41 | 0.00 |
| Spleen 5 | 0.00 | 0.00 | 1.34 | 2.32 | 0.00 | 0.00 | 0.00 | 0.00 | 0.00 |
| Stomach 1 | 1.62 | 0.00 | 4.00 | 0.00 | 0.00 | 0.00 | 0.00 | 0.00 | 0.00 |
| Stomach 2 | 0.42 | 0.00 | 0.95 | 0.00 | 0.57 | 0.00 | 0.00 | 0.00 | 0.00 |
| Stomach 3 | 1.43 | 0.23 | 1.28 | 0.00 | 0.47 | 0.00 | 2.76 | 0.00 | 0.00 |
| Stomach 4 | 0.63 | 0.00 | 1.85 | 0.00 | 0.85 | 0.00 | 0.00 | 0.00 | 0.00 |
| Stomach 5 | 0.32 | 0.00 | 1.13 | 0.36 | 0.51 | 0.00 | 0.00 | 0.00 | 0.00 |
| Testis 1 | 0.00 | 0.00 | 0.00 | 0.00 | 0.00 | 0.00 | 4.97 | 2.27 | 0.00 |
| Testis 2 | 0.00 | 0.00 | 0.00 | 0.00 | 0.00 | 0.00 | 4.94 | 2.10 | 0.00 |
| Testis 3 | 0.00 | 0.00 | 0.00 | 0.58 | 0.00 | 0.00 | 2.41 | 0.00 | 0.00 |
| Testis 4 | 0.00 | 0.00 | 0.00 | 0.74 | 0.00 | 0.00 | 5.76 | 0.00 | 0.00 |
| Testis 5 | 0.00 | 0.00 | 0.00 | 0.60 | 0.00 | 0.00 | 4.68 | 0.00 | 0.00 |
| Thyroid 1 | 0.00 | 0.00 | 0.00 | 0.00 | 0.00 | 0.00 | 0.00 | 0.00 | 15.16 |
| Thyroid 2 | 0.00 | 0.00 | 0.00 | 0.00 | 0.00 | 0.00 | 0.00 | 0.00 | 11.88 |
| Thyroid 3 | 0.00 | 0.00 | 0.00 | 0.00 | 0.00 | 0.00 | 0.00 | 0.00 | 10.30 |
| Thyroid 4 | 0.00 | 0.00 | 0.00 | 0.00 | 0.00 | 0.00 | 0.00 | 0.00 | 12.63 |
| Thyroid 5 | 0.00 | 0.00 | 0.00 | 0.00 | 0.00 | 0.00 | 0.00 | 0.00 | 12.32 |
| Uterus 1 | 0.00 | 0.00 | 0.84 | 1.62 | 0.00 | 0.00 | 0.00 | 0.00 | 0.00 |
| Uterus 2 | 0.00 | 0.00 | 2.26 | 1.06 | 0.00 | 0.00 | 0.00 | 1.79 | 0.00 |
| Uterus 3 | 0.00 | 0.00 | 2.25 | 0.00 | 0.00 | 0.00 | 0.00 | 0.00 | 0.00 |
| Uterus 4 | 0.00 | 0.00 | 1.69 | 1.49 | 0.00 | 0.00 | 0.00 | 0.00 | 0.00 |
| Uterus 5 | 0.00 | 0.00 | 1.30 | 0.91 | 0.00 | 0.00 | 0.00 | 0.00 | 0.00 |
| Serum 1 | 0.00 | 0.00 | 0.00 | 0.00 | 0.00 | 0.00 | 0.00 | 0.00 | 0.00 |
| Serum 2 | 0.00 | 0.00 | 0.00 | 0.00 | 0.00 | 0.00 | 0.00 | 0.00 | 0.00 |
| Serum 3 | 0.00 | 0.00 | 0.00 | 2.31 | 0.00 | 0.00 | 0.00 | 0.00 | 0.00 |
| Serum 4 | 0.00 | 0.00 | 0.00 | 0.00 | 0.00 | 0.00 | 0.00 | 0.00 | 0.00 |
| Serum 5 | 0.00 | 0.00 | 0.00 | 0.00 | 0.00 | 0.00 | 0.00 | 0.00 | 0.00 |
| Exosome 1 | 0.00 | 0.00 | 0.00 | 2.93 | 0.00 | 0.00 | 0.00 | 0.00 | 0.00 |
| Exosome 2 | 0.00 | 0.00 | 0.00 | 3.06 | 0.00 | 0.00 | 0.00 | 0.00 | 0.00 |
| Exosome 3 | 0.00 | 0.00 | 0.00 | 3.30 | 0.00 | 0.00 | 0.00 | 0.00 | 0.00 |
| Exosome 4 | 0.00 | 0.00 | 0.00 | 3.60 | 0.00 | 0.00 | 0.00 | 0.00 | 0.00 |
| Exosome 5 | 0.00 | 0.00 | 0.00 | 3.17 | 0.00 | 0.00 | 0.00 | 0.00 | 0.00 |

| Glycoform | **61** | **62** | **63** | **64** | **65** | **66** | **67** | **68** | **69** | **70** |
| --- | --- | --- | --- | --- | --- | --- | --- | --- | --- | --- |
| *m/z* | 2499.95 | 2502 | 2505.99 | 2508.99 | 2518 | 2518.98 | 2521.98 | 2524.98 | 2534.98 | 2537.97 |
| Abbreviation | H8N3D1 | H3N7D1 | H4N4D2A1 | H5N4D3 | H4N7 | H4N4A2 | H5N4D1A1 | H6N4D2 | H3N4D1G2 | H5N4D1G1 |
| Brain 1 | 0.00 | 0.00 | 0.00 | 0.00 | 0.00 | 0.00 | 0.00 | 0.47 | 0.00 | 0.00 |
| Brain 2 | 0.00 | 0.00 | 0.00 | 0.00 | 0.00 | 0.00 | 0.00 | 0.00 | 0.00 | 0.00 |
| Brain 3 | 0.00 | 0.18 | 1.81 | 0.72 | 0.00 | 0.00 | 0.00 | 0.00 | 0.00 | 0.00 |
| Brain 4 | 0.27 | 0.00 | 0.00 | 0.00 | 0.00 | 0.00 | 0.00 | 0.00 | 0.00 | 0.00 |
| Brain 5 | 0.00 | 0.00 | 0.00 | 0.00 | 0.00 | 0.00 | 0.00 | 0.00 | 0.00 | 0.00 |
| Femur 1 | 1.07 | 0.00 | 0.00 | 0.00 | 0.00 | 0.00 | 0.00 | 0.00 | 0.00 | 1.38 |
| Femur 2 | 6.93 | 0.00 | 1.29 | 0.00 | 0.00 | 0.00 | 0.00 | 0.00 | 0.00 | 3.73 |
| Femur 3 | 2.83 | 0.00 | 0.00 | 0.00 | 0.00 | 0.00 | 1.94 | 0.00 | 0.00 | 2.71 |
| Femur 4 | 11.36 | 0.00 | 1.10 | 0.00 | 0.00 | 0.00 | 0.00 | 0.00 | 0.00 | 1.94 |
| Femur 5 | 0.00 | 0.00 | 0.00 | 0.00 | 0.00 | 0.00 | 2.86 | 0.00 | 0.00 | 3.21 |
| Heart 1 | 0.00 | 0.00 | 0.00 | 0.00 | 0.00 | 0.00 | 0.00 | 0.00 | 0.00 | 1.53 |
| Heart 2 | 0.00 | 0.00 | 0.00 | 0.00 | 0.00 | 0.00 | 0.27 | 0.00 | 0.00 | 0.47 |
| Heart 3 | 0.40 | 0.00 | 0.00 | 0.00 | 0.00 | 0.00 | 0.37 | 0.00 | 0.00 | 0.73 |
| Heart 4 | 0.29 | 0.00 | 0.00 | 0.00 | 0.00 | 0.00 | 0.18 | 0.00 | 0.00 | 0.43 |
| Heart 5 | 0.00 | 0.00 | 0.00 | 0.00 | 0.00 | 0.00 | 0.00 | 0.00 | 0.00 | 0.49 |
| Intestines 1 | 0.00 | 0.00 | 0.00 | 0.00 | 0.00 | 0.00 | 0.00 | 0.00 | 0.00 | 2.02 |
| Intestines 2 | 1.27 | 0.00 | 0.00 | 0.00 | 0.46 | 0.00 | 0.00 | 0.00 | 0.00 | 2.14 |
| Intestines 3 | 0.00 | 0.00 | 0.00 | 1.74 | 0.65 | 0.00 | 0.00 | 0.00 | 0.00 | 1.38 |
| Intestines 4 | 1.28 | 0.00 | 0.00 | 0.00 | 0.69 | 0.00 | 0.00 | 0.00 | 0.00 | 1.45 |
| Intestines 5 | 1.51 | 0.00 | 0.00 | 0.00 | 0.63 | 0.00 | 0.00 | 0.00 | 0.00 | 1.16 |
| Kidney 1 | 1.52 | 0.00 | 0.00 | 0.00 | 0.00 | 0.00 | 0.00 | 0.00 | 0.00 | 0.91 |
| Kidney 2 | 0.00 | 0.00 | 1.10 | 1.63 | 0.00 | 0.00 | 0.00 | 0.00 | 0.00 | 0.70 |
| Kidney 3 | 0.00 | 0.00 | 0.00 | 1.62 | 0.00 | 0.00 | 0.00 | 0.00 | 0.00 | 0.00 |
| Kidney 4 | 7.50 | 0.00 | 0.00 | 1.28 | 0.00 | 0.00 | 0.00 | 0.00 | 0.00 | 0.00 |
| Kidney 5 | 0.90 | 0.00 | 0.73 | 2.48 | 0.00 | 0.00 | 0.00 | 0.00 | 0.00 | 0.69 |
| Liver 1 | 0.00 | 0.00 | 1.63 | 0.00 | 0.00 | 0.00 | 0.00 | 0.00 | 0.00 | 0.00 |
| Liver 2 | 0.00 | 0.00 | 0.00 | 0.00 | 0.00 | 0.00 | 0.00 | 0.00 | 0.00 | 0.00 |
| Liver 3 | 0.00 | 0.00 | 1.33 | 0.70 | 0.00 | 0.00 | 0.00 | 0.00 | 0.00 | 0.00 |
| Liver 4 | 0.00 | 0.00 | 0.00 | 0.00 | 0.00 | 0.00 | 0.00 | 0.00 | 0.23 | 0.11 |
| Liver 5 | 0.00 | 0.00 | 1.00 | 0.40 | 0.00 | 0.00 | 0.00 | 0.00 | 0.00 | 0.05 |
| Lung 1 | 0.00 | 0.00 | 0.00 | 0.00 | 0.00 | 0.87 | 4.69 | 0.00 | 0.79 | 5.93 |
| Lung 2 | 0.00 | 0.00 | 2.22 | 0.00 | 0.00 | 1.26 | 7.57 | 0.00 | 0.00 | 6.54 |
| Lung 3 | 0.00 | 0.00 | 2.22 | 0.00 | 0.00 | 0.00 | 31.11 | 0.00 | 1.12 | 13.20 |
| Lung 4 | 0.91 | 0.00 | 0.75 | 0.00 | 0.00 | 0.70 | 2.14 | 0.00 | 0.00 | 2.74 |
| Lung 5 | 0.00 | 0.00 | 1.34 | 0.00 | 0.00 | 0.86 | 4.32 | 0.00 | 0.00 | 5.73 |
| Muscle 1 | 0.00 | 0.00 | 2.35 | 0.00 | 0.00 | 0.00 | 0.00 | 0.00 | 0.00 | 4.29 |
| Muscle 2 | 0.00 | 0.00 | 1.42 | 0.00 | 0.00 | 0.00 | 0.00 | 0.00 | 0.00 | 3.48 |
| Muscle 3 | 2.37 | 0.00 | 1.54 | 0.00 | 0.00 | 0.00 | 0.00 | 0.00 | 0.00 | 2.45 |
| Muscle 4 | 2.00 | 0.00 | 0.81 | 0.00 | 0.00 | 0.00 | 0.00 | 0.00 | 0.00 | 0.91 |
| Muscle 5 | 0.00 | 0.00 | 0.00 | 0.00 | 0.00 | 0.00 | 0.00 | 0.00 | 0.00 | 3.37 |
| Ovary 1 | 0.00 | 0.00 | 3.24 | 0.00 | 0.00 | 0.00 | 0.00 | 0.00 | 0.00 | 4.23 |
| Ovary 2 | 0.00 | 0.00 | 0.00 | 0.00 | 0.00 | 0.84 | 3.21 | 0.00 | 0.35 | 2.61 |
| Ovary 3 | 0.00 | 0.00 | 0.00 | 0.00 | 0.00 | 0.93 | 3.66 | 0.00 | 0.00 | 4.39 |
| Ovary 4 | 0.00 | 0.00 | 0.00 | 0.00 | 0.34 | 0.00 | 0.00 | 0.00 | 0.00 | 5.15 |
| Ovary 5 | 0.00 | 0.00 | 0.46 | 0.00 | 0.00 | 0.00 | 0.00 | 0.00 | 0.00 | 6.59 |
| Pancreas 1 | 0.00 | 0.00 | 0.00 | 0.00 | 0.00 | 0.00 | 0.00 | 5.89 | 0.00 | 1.61 |
| Pancreas 2 | 1.77 | 0.00 | 0.96 | 0.00 | 0.00 | 0.00 | 0.00 | 0.00 | 0.00 | 1.42 |
| Pancreas 3 | 2.81 | 0.00 | 0.00 | 0.00 | 0.00 | 0.00 | 0.00 | 1.80 | 0.00 | 0.00 |
| Pancreas 4 | 0.00 | 0.00 | 0.00 | 0.00 | 0.00 | 0.00 | 0.00 | 2.98 | 0.00 | 0.00 |
| Pancreas 5 | 3.85 | 0.00 | 0.00 | 4.40 | 0.00 | 0.00 | 0.00 | 0.00 | 0.00 | 0.00 |
| Skin 1 | 0.00 | 0.00 | 2.07 | 1.01 | 0.00 | 0.00 | 0.00 | 0.00 | 0.00 | 11.44 |
| Skin 2 | 0.00 | 0.00 | 0.00 | 0.00 | 0.25 | 0.00 | 0.00 | 0.00 | 0.00 | 15.17 |
| Skin 3 | 0.00 | 0.82 | 0.00 | 0.00 | 0.00 | 1.03 | 3.86 | 0.00 | 0.00 | 16.76 |
| Skin 4 | 0.00 | 0.94 | 0.00 | 0.00 | 0.00 | 1.32 | 5.86 | 0.00 | 0.00 | 23.94 |
| Skin 5 | 0.00 | 0.00 | 0.00 | 0.00 | 0.00 | 0.79 | 5.57 | 0.00 | 0.00 | 20.91 |
| Spleen 1 | 0.00 | 0.00 | 0.00 | 0.00 | 0.00 | 0.00 | 0.00 | 0.00 | 0.00 | 2.77 |
| Spleen 2 | 1.53 | 0.00 | 1.36 | 0.00 | 0.00 | 0.00 | 0.00 | 0.00 | 0.00 | 5.74 |
| Spleen 3 | 5.97 | 0.00 | 1.58 | 0.00 | 0.00 | 0.00 | 0.00 | 0.00 | 0.00 | 3.21 |
| Spleen 4 | 7.25 | 0.00 | 1.49 | 0.00 | 0.00 | 0.00 | 0.00 | 0.00 | 0.00 | 3.11 |
| Spleen 5 | 0.00 | 0.00 | 1.86 | 0.00 | 0.00 | 0.00 | 0.00 | 0.00 | 0.00 | 4.88 |
| Stomach 1 | 0.00 | 0.00 | 0.00 | 0.00 | 0.00 | 0.00 | 0.00 | 0.00 | 0.00 | 2.56 |
| Stomach 2 | 0.00 | 0.00 | 0.00 | 0.00 | 0.00 | 0.00 | 0.00 | 0.00 | 0.00 | 0.71 |
| Stomach 3 | 0.00 | 0.00 | 0.00 | 0.00 | 0.00 | 0.50 | 0.00 | 0.00 | 0.00 | 0.00 |
| Stomach 4 | 0.00 | 0.00 | 0.00 | 0.00 | 0.00 | 0.00 | 0.00 | 0.00 | 0.00 | 1.12 |
| Stomach 5 | 0.24 | 0.00 | 0.00 | 0.00 | 0.00 | 0.00 | 0.00 | 0.00 | 0.00 | 0.50 |
| Testis 1 | 0.00 | 0.00 | 0.00 | 0.00 | 0.00 | 0.00 | 0.00 | 0.00 | 0.00 | 0.83 |
| Testis 2 | 0.00 | 0.00 | 0.00 | 0.00 | 0.00 | 0.00 | 0.00 | 0.00 | 0.00 | 0.00 |
| Testis 3 | 0.00 | 0.00 | 0.00 | 0.00 | 0.00 | 0.00 | 0.00 | 0.00 | 0.00 | 0.58 |
| Testis 4 | 0.00 | 0.00 | 0.00 | 0.00 | 0.32 | 0.00 | 0.00 | 0.00 | 0.00 | 0.59 |
| Testis 5 | 0.00 | 0.00 | 0.00 | 0.00 | 0.27 | 0.00 | 0.00 | 0.00 | 0.00 | 0.52 |
| Thyroid 1 | 0.00 | 0.00 | 0.00 | 0.00 | 0.00 | 0.00 | 278.35 | 0.00 | 0.00 | 0.00 |
| Thyroid 2 | 0.00 | 0.00 | 0.00 | 0.00 | 0.00 | 0.00 | 196.31 | 0.00 | 0.00 | 0.00 |
| Thyroid 3 | 0.00 | 0.00 | 0.00 | 0.00 | 0.00 | 0.00 | 199.42 | 0.00 | 0.00 | 0.00 |
| Thyroid 4 | 0.00 | 0.00 | 0.00 | 0.00 | 0.00 | 0.00 | 303.87 | 0.00 | 0.00 | 0.00 |
| Thyroid 5 | 0.00 | 0.00 | 0.00 | 0.00 | 0.00 | 0.00 | 251.80 | 0.00 | 0.00 | 0.00 |
| Uterus 1 | 0.00 | 0.00 | 0.00 | 0.00 | 0.00 | 1.11 | 5.70 | 0.00 | 0.99 | 3.83 |
| Uterus 2 | 0.00 | 0.56 | 0.00 | 0.00 | 0.00 | 1.07 | 10.47 | 0.00 | 0.79 | 7.46 |
| Uterus 3 | 0.00 | 0.00 | 0.00 | 0.00 | 0.00 | 1.12 | 13.27 | 0.00 | 0.91 | 7.94 |
| Uterus 4 | 0.00 | 0.00 | 0.00 | 0.00 | 0.00 | 0.91 | 5.54 | 0.00 | 0.00 | 4.73 |
| Uterus 5 | 0.72 | 0.00 | 0.00 | 0.00 | 0.00 | 0.94 | 5.01 | 0.00 | 0.00 | 3.98 |
| Serum 1 | 0.00 | 0.00 | 0.00 | 0.00 | 0.00 | 0.00 | 0.00 | 0.00 | 0.00 | 7.22 |
| Serum 2 | 0.00 | 0.00 | 0.00 | 0.00 | 0.00 | 0.00 | 0.00 | 0.00 | 0.00 | 6.08 |
| Serum 3 | 0.00 | 0.00 | 1.56 | 0.00 | 0.00 | 0.00 | 0.00 | 0.00 | 0.00 | 5.12 |
| Serum 4 | 0.00 | 0.00 | 1.27 | 0.00 | 0.00 | 0.00 | 0.00 | 0.00 | 0.00 | 7.73 |
| Serum 5 | 0.00 | 0.00 | 0.00 | 0.00 | 0.00 | 0.00 | 0.00 | 0.00 | 0.00 | 6.19 |
| Exosome 1 | 4.63 | 0.00 | 0.00 | 0.00 | 1.22 | 0.00 | 0.00 | 0.00 | 0.00 | 19.81 |
| Exosome 2 | 0.00 | 0.00 | 6.89 | 0.00 | 0.21 | 0.00 | 0.00 | 0.00 | 0.00 | 20.17 |
| Exosome 3 | 0.00 | 0.00 | 1.24 | 0.00 | 0.74 | 0.00 | 0.00 | 0.00 | 0.00 | 20.70 |
| Exosome 4 | 7.49 | 0.00 | 0.00 | 0.00 | 1.57 | 0.00 | 0.00 | 0.00 | 0.00 | 45.51 |
| Exosome 5 | 0.00 | 0.00 | 0.00 | 0.00 | 0.88 | 0.00 | 0.00 | 0.00 | 0.00 | 37.65 |

| Glycoform | **71** | **72** | **73** | **74** | **75** | **76** | **77** | **78** | **79** | **80** |
| --- | --- | --- | --- | --- | --- | --- | --- | --- | --- | --- |
| *m/z* | 2540.97 | 2550.01 | 2553.97 | 2563.01 | 2566.01 | 2579 | 2582 | 2588.99 | 2591.04 | 2607.03 |
| Abbreviation | H7N4D1 | H4N5D3 | H6N4G1 | H4N5D1A1 | H5N5D2 | H5N5A1 | H6N5D1 | H8N2D3 | H3N6D3 | H4N6D2 |
| Brain 1 | 0.00 | 0.00 | 0.00 | 2.36 | 0.92 | 0.00 | 0.00 | 0.00 | 0.00 | 1.83 |
| Brain 2 | 0.00 | 0.16 | 0.00 | 0.00 | 0.00 | 0.00 | 0.00 | 0.00 | 0.00 | 0.21 |
| Brain 3 | 0.00 | 3.44 | 0.00 | 1.27 | 0.35 | 0.00 | 0.00 | 0.37 | 0.00 | 0.29 |
| Brain 4 | 0.00 | 0.00 | 0.00 | 0.00 | 0.00 | 0.00 | 0.00 | 0.00 | 0.00 | 0.16 |
| Brain 5 | 0.00 | 1.29 | 0.00 | 0.00 | 0.00 | 0.00 | 0.00 | 0.19 | 0.00 | 0.25 |
| Femur 1 | 0.00 | 0.00 | 0.66 | 0.00 | 0.00 | 0.00 | 0.00 | 0.00 | 0.00 | 0.00 |
| Femur 2 | 0.00 | 0.00 | 0.00 | 0.00 | 0.00 | 0.00 | 0.00 | 0.00 | 0.00 | 0.00 |
| Femur 3 | 0.00 | 0.00 | 0.00 | 0.00 | 0.00 | 0.00 | 0.00 | 0.00 | 0.00 | 0.00 |
| Femur 4 | 0.00 | 0.00 | 0.00 | 0.00 | 0.00 | 0.00 | 0.00 | 0.00 | 0.00 | 0.00 |
| Femur 5 | 0.00 | 0.00 | 0.00 | 0.00 | 0.00 | 0.00 | 0.00 | 0.00 | 0.00 | 0.00 |
| Heart 1 | 0.00 | 1.27 | 0.00 | 0.00 | 0.00 | 0.00 | 0.00 | 0.00 | 0.00 | 0.00 |
| Heart 2 | 0.00 | 0.00 | 0.00 | 0.18 | 0.00 | 0.00 | 0.00 | 0.00 | 0.00 | 0.00 |
| Heart 3 | 0.00 | 0.00 | 0.00 | 0.00 | 0.00 | 0.00 | 0.00 | 0.00 | 0.00 | 0.00 |
| Heart 4 | 0.00 | 0.00 | 0.00 | 0.00 | 0.00 | 0.00 | 0.00 | 0.00 | 0.00 | 0.00 |
| Heart 5 | 0.00 | 0.00 | 0.00 | 0.00 | 0.00 | 0.00 | 0.00 | 0.00 | 0.00 | 0.00 |
| Intestines 1 | 0.00 | 0.00 | 0.00 | 0.00 | 2.40 | 0.00 | 0.00 | 0.00 | 0.00 | 0.00 |
| Intestines 2 | 0.00 | 0.58 | 0.00 | 0.55 | 1.33 | 0.84 | 0.00 | 0.00 | 0.00 | 0.00 |
| Intestines 3 | 0.00 | 0.00 | 0.00 | 0.00 | 0.00 | 0.00 | 0.00 | 0.00 | 0.00 | 0.00 |
| Intestines 4 | 0.00 | 0.00 | 0.00 | 0.00 | 1.18 | 0.00 | 0.00 | 0.00 | 0.00 | 0.00 |
| Intestines 5 | 0.00 | 0.00 | 0.00 | 0.00 | 1.06 | 0.00 | 0.00 | 0.00 | 0.00 | 0.00 |
| Kidney 1 | 0.00 | 1.19 | 0.00 | 0.00 | 6.09 | 0.00 | 0.00 | 0.00 | 0.00 | 0.00 |
| Kidney 2 | 0.00 | 0.00 | 0.00 | 0.00 | 3.87 | 0.00 | 0.00 | 0.00 | 0.00 | 0.00 |
| Kidney 3 | 0.00 | 0.00 | 0.00 | 0.00 | 4.51 | 0.00 | 0.00 | 0.00 | 0.00 | 0.00 |
| Kidney 4 | 0.00 | 0.00 | 0.00 | 0.00 | 2.10 | 0.00 | 0.00 | 0.00 | 0.00 | 0.00 |
| Kidney 5 | 0.00 | 0.75 | 0.00 | 0.00 | 5.44 | 0.00 | 0.00 | 0.00 | 0.00 | 0.00 |
| Liver 1 | 0.00 | 0.00 | 0.00 | 0.00 | 0.00 | 0.00 | 0.00 | 0.00 | 0.00 | 0.00 |
| Liver 2 | 0.00 | 0.32 | 0.00 | 0.00 | 0.00 | 0.00 | 0.00 | 0.00 | 0.00 | 0.00 |
| Liver 3 | 0.00 | 0.00 | 0.00 | 0.00 | 0.11 | 0.00 | 0.00 | 0.20 | 0.00 | 0.00 |
| Liver 4 | 0.00 | 0.00 | 0.00 | 0.00 | 0.13 | 0.12 | 0.00 | 0.23 | 0.00 | 0.00 |
| Liver 5 | 0.00 | 0.00 | 0.00 | 0.00 | 0.00 | 0.00 | 0.00 | 0.18 | 0.00 | 0.00 |
| Lung 1 | 1.87 | 0.00 | 2.08 | 0.00 | 0.92 | 0.00 | 0.98 | 0.00 | 0.00 | 0.00 |
| Lung 2 | 0.00 | 1.32 | 0.00 | 0.00 | 0.00 | 0.00 | 0.00 | 0.00 | 0.00 | 0.00 |
| Lung 3 | 0.00 | 1.54 | 0.00 | 0.00 | 0.00 | 0.00 | 3.08 | 0.00 | 0.00 | 0.00 |
| Lung 4 | 0.00 | 0.00 | 0.00 | 0.00 | 0.00 | 0.00 | 0.00 | 0.00 | 0.00 | 0.00 |
| Lung 5 | 1.42 | 0.00 | 1.29 | 0.00 | 0.00 | 0.00 | 0.00 | 0.00 | 0.00 | 0.00 |
| Muscle 1 | 0.00 | 0.00 | 0.00 | 0.00 | 0.00 | 0.00 | 0.00 | 0.00 | 0.00 | 0.00 |
| Muscle 2 | 0.00 | 0.00 | 0.00 | 0.00 | 0.00 | 0.00 | 0.00 | 0.00 | 0.00 | 0.00 |
| Muscle 3 | 0.00 | 0.00 | 0.00 | 0.00 | 0.00 | 0.00 | 0.00 | 0.00 | 0.00 | 0.00 |
| Muscle 4 | 0.00 | 0.00 | 0.00 | 0.00 | 0.00 | 0.00 | 0.00 | 0.00 | 0.00 | 0.00 |
| Muscle 5 | 0.00 | 0.00 | 0.00 | 0.00 | 0.00 | 0.00 | 0.00 | 0.00 | 0.00 | 0.00 |
| Ovary 1 | 0.00 | 0.00 | 0.00 | 0.00 | 0.00 | 0.00 | 0.00 | 0.00 | 0.00 | 0.00 |
| Ovary 2 | 0.00 | 0.00 | 0.00 | 0.00 | 0.00 | 0.00 | 0.00 | 0.00 | 0.00 | 0.00 |
| Ovary 3 | 0.00 | 0.00 | 0.00 | 0.00 | 0.00 | 0.00 | 0.00 | 0.00 | 0.00 | 0.00 |
| Ovary 4 | 0.00 | 0.00 | 0.00 | 0.00 | 0.00 | 0.00 | 0.00 | 0.00 | 0.00 | 0.00 |
| Ovary 5 | 0.00 | 0.00 | 0.00 | 0.00 | 0.00 | 0.00 | 0.00 | 0.00 | 0.00 | 0.00 |
| Pancreas 1 | 4.71 | 0.00 | 0.00 | 0.00 | 0.00 | 0.00 | 0.00 | 0.00 | 0.00 | 0.00 |
| Pancreas 2 | 0.00 | 0.00 | 0.00 | 0.00 | 0.00 | 0.00 | 0.00 | 0.00 | 0.00 | 0.00 |
| Pancreas 3 | 0.00 | 0.00 | 0.00 | 0.00 | 0.00 | 0.00 | 0.00 | 0.00 | 0.00 | 0.00 |
| Pancreas 4 | 0.00 | 0.00 | 0.00 | 0.00 | 0.00 | 0.00 | 0.00 | 0.00 | 0.00 | 0.00 |
| Pancreas 5 | 0.00 | 0.00 | 0.00 | 0.00 | 0.00 | 0.00 | 0.00 | 0.00 | 0.00 | 0.00 |
| Skin 1 | 0.00 | 0.00 | 0.00 | 0.00 | 0.00 | 0.00 | 0.00 | 0.00 | 0.00 | 0.00 |
| Skin 2 | 0.00 | 0.00 | 0.00 | 0.00 | 0.00 | 0.00 | 0.00 | 0.00 | 0.00 | 0.00 |
| Skin 3 | 0.00 | 0.00 | 0.00 | 0.00 | 0.00 | 0.00 | 0.00 | 0.00 | 0.00 | 0.00 |
| Skin 4 | 0.00 | 0.00 | 0.00 | 0.00 | 0.00 | 1.11 | 4.04 | 0.00 | 0.00 | 0.00 |
| Skin 5 | 0.00 | 0.00 | 1.30 | 0.00 | 0.00 | 0.00 | 0.00 | 0.00 | 0.00 | 0.00 |
| Spleen 1 | 0.00 | 0.00 | 0.00 | 0.00 | 0.00 | 0.00 | 0.00 | 0.00 | 0.00 | 0.00 |
| Spleen 2 | 0.00 | 0.00 | 1.83 | 0.00 | 0.00 | 0.00 | 0.00 | 0.00 | 0.00 | 0.00 |
| Spleen 3 | 0.00 | 0.00 | 0.00 | 0.00 | 0.00 | 0.00 | 0.00 | 0.00 | 0.00 | 0.00 |
| Spleen 4 | 0.00 | 0.00 | 0.00 | 0.00 | 0.00 | 0.00 | 0.00 | 0.00 | 0.00 | 0.00 |
| Spleen 5 | 0.00 | 0.00 | 0.00 | 0.00 | 0.00 | 0.00 | 0.00 | 0.00 | 0.00 | 0.00 |
| Stomach 1 | 0.00 | 1.82 | 0.00 | 0.00 | 1.27 | 0.00 | 0.00 | 0.00 | 3.55 | 0.00 |
| Stomach 2 | 0.00 | 0.32 | 0.00 | 0.00 | 0.30 | 0.00 | 0.00 | 0.00 | 1.46 | 0.00 |
| Stomach 3 | 0.00 | 0.00 | 0.00 | 0.00 | 0.46 | 0.00 | 0.00 | 0.00 | 1.30 | 0.00 |
| Stomach 4 | 0.00 | 0.52 | 0.00 | 0.00 | 0.56 | 0.00 | 0.00 | 0.00 | 2.80 | 0.00 |
| Stomach 5 | 0.00 | 0.63 | 0.00 | 0.00 | 0.00 | 0.00 | 0.00 | 0.00 | 1.30 | 0.00 |
| Testis 1 | 0.00 | 0.00 | 0.00 | 0.00 | 0.00 | 0.00 | 1.30 | 0.00 | 0.00 | 0.00 |
| Testis 2 | 0.00 | 0.00 | 0.00 | 0.00 | 0.00 | 0.00 | 0.00 | 0.00 | 0.00 | 0.00 |
| Testis 3 | 0.00 | 0.00 | 0.00 | 0.00 | 0.00 | 0.00 | 0.50 | 0.00 | 0.00 | 0.00 |
| Testis 4 | 0.00 | 0.00 | 0.00 | 0.00 | 0.00 | 0.00 | 0.77 | 0.00 | 0.00 | 0.00 |
| Testis 5 | 0.00 | 0.00 | 0.00 | 0.00 | 0.00 | 0.00 | 0.37 | 0.00 | 0.00 | 0.00 |
| Thyroid 1 | 0.00 | 0.00 | 0.00 | 0.00 | 0.00 | 0.00 | 22.91 | 0.00 | 0.00 | 0.00 |
| Thyroid 2 | 0.00 | 0.00 | 0.00 | 0.00 | 0.00 | 0.00 | 10.71 | 0.00 | 0.00 | 0.00 |
| Thyroid 3 | 0.00 | 0.00 | 0.00 | 0.00 | 0.00 | 0.00 | 11.49 | 0.00 | 0.00 | 0.00 |
| Thyroid 4 | 0.00 | 0.00 | 0.00 | 0.00 | 0.00 | 0.00 | 27.91 | 0.00 | 0.00 | 0.00 |
| Thyroid 5 | 0.00 | 0.00 | 0.00 | 0.00 | 0.00 | 2.92 | 16.09 | 0.00 | 0.00 | 0.00 |
| Uterus 1 | 0.00 | 0.00 | 0.00 | 0.00 | 0.00 | 0.00 | 0.00 | 0.00 | 0.00 | 0.00 |
| Uterus 2 | 0.00 | 0.00 | 0.99 | 0.00 | 0.00 | 0.00 | 0.00 | 0.00 | 0.00 | 0.00 |
| Uterus 3 | 0.00 | 0.00 | 0.00 | 0.00 | 0.00 | 0.00 | 0.00 | 0.00 | 0.00 | 0.00 |
| Uterus 4 | 0.00 | 0.00 | 0.00 | 0.00 | 0.00 | 0.00 | 0.00 | 0.00 | 0.00 | 0.00 |
| Uterus 5 | 0.00 | 0.00 | 0.00 | 0.00 | 0.00 | 0.00 | 0.00 | 0.00 | 0.00 | 0.00 |
| Serum 1 | 0.00 | 0.00 | 0.00 | 0.00 | 0.00 | 0.00 | 0.00 | 0.00 | 0.00 | 0.00 |
| Serum 2 | 0.00 | 0.00 | 0.00 | 0.00 | 0.00 | 0.00 | 0.00 | 0.00 | 0.00 | 0.00 |
| Serum 3 | 0.00 | 0.00 | 0.00 | 0.00 | 0.00 | 0.00 | 0.00 | 0.00 | 0.00 | 0.00 |
| Serum 4 | 0.00 | 0.00 | 0.00 | 0.00 | 0.00 | 0.00 | 0.00 | 0.00 | 0.00 | 0.00 |
| Serum 5 | 0.00 | 0.00 | 0.00 | 0.00 | 0.00 | 0.00 | 0.00 | 0.00 | 0.00 | 0.00 |
| Exosome 1 | 0.00 | 0.00 | 0.00 | 0.00 | 0.00 | 0.00 | 0.00 | 0.00 | 0.00 | 0.00 |
| Exosome 2 | 0.00 | 0.00 | 0.00 | 0.00 | 0.00 | 0.00 | 0.00 | 0.00 | 0.00 | 0.00 |
| Exosome 3 | 0.00 | 0.00 | 0.00 | 0.00 | 0.00 | 0.00 | 0.00 | 0.00 | 0.00 | 0.00 |
| Exosome 4 | 0.00 | 0.00 | 0.00 | 0.00 | 0.00 | 0.00 | 0.00 | 0.00 | 0.00 | 0.00 |
| Exosome 5 | 0.00 | 0.00 | 0.00 | 0.00 | 0.00 | 0.00 | 0.00 | 0.00 | 0.00 | 0.00 |

| Glycoform | **81** | **82** | **83** | **84** | **85** | **86** | **87** | **88** | **89** | **90** |
| --- | --- | --- | --- | --- | --- | --- | --- | --- | --- | --- |
| *m/z* | 2623.03 | 2671.04 | 2680.05 | 2681.03 | 2684.03 | 2697.03 | 2700.03 | 2712.06 | 2713.02 | 2725.06 |
| Abbreviation | H5N6D1 | H6N4D3 | H5N7 | H5N4A2 | H6N4D1A1 | H5N4A1G1 | H7N4A1 | H5N5D3 | H5N4G2 | H5N5D1A1 |
| Brain 1 | 0.00 | 0.00 | 0.00 | 0.00 | 0.00 | 0.00 | 0.00 | 2.50 | 0.00 | 0.00 |
| Brain 2 | 0.00 | 0.00 | 0.00 | 0.00 | 0.00 | 0.00 | 0.00 | 0.45 | 0.00 | 0.00 |
| Brain 3 | 0.00 | 0.00 | 0.00 | 0.00 | 0.00 | 0.00 | 0.00 | 0.29 | 0.00 | 0.00 |
| Brain 4 | 0.00 | 0.00 | 0.00 | 0.00 | 0.00 | 0.00 | 0.00 | 0.19 | 0.00 | 0.00 |
| Brain 5 | 0.00 | 0.00 | 0.00 | 0.00 | 0.00 | 0.00 | 0.00 | 0.30 | 0.00 | 0.00 |
| Femur 1 | 0.00 | 0.00 | 0.00 | 0.00 | 0.00 | 0.00 | 0.00 | 0.00 | 12.97 | 0.00 |
| Femur 2 | 0.00 | 0.00 | 0.00 | 0.00 | 0.00 | 0.00 | 0.00 | 0.00 | 16.88 | 0.00 |
| Femur 3 | 0.00 | 0.00 | 0.00 | 0.00 | 0.00 | 0.00 | 0.00 | 0.00 | 12.93 | 0.00 |
| Femur 4 | 0.00 | 0.00 | 0.00 | 0.00 | 0.00 | 0.00 | 0.00 | 0.00 | 9.17 | 0.00 |
| Femur 5 | 0.00 | 0.00 | 0.00 | 0.00 | 0.00 | 0.00 | 0.00 | 0.00 | 16.45 | 0.00 |
| Heart 1 | 0.00 | 0.00 | 0.00 | 0.00 | 0.00 | 0.00 | 0.00 | 0.00 | 5.50 | 0.00 |
| Heart 2 | 0.00 | 0.00 | 0.00 | 0.20 | 0.00 | 0.00 | 0.00 | 0.00 | 7.89 | 0.00 |
| Heart 3 | 0.00 | 0.00 | 0.00 | 0.21 | 0.00 | 0.00 | 0.00 | 0.00 | 9.59 | 0.00 |
| Heart 4 | 0.00 | 0.00 | 0.00 | 0.13 | 0.00 | 0.00 | 0.00 | 0.00 | 5.49 | 0.00 |
| Heart 5 | 0.00 | 0.00 | 0.00 | 0.00 | 0.00 | 0.00 | 0.00 | 0.00 | 5.93 | 0.00 |
| Intestines 1 | 4.99 | 0.00 | 0.00 | 0.00 | 0.00 | 0.00 | 0.00 | 2.25 | 0.00 | 1.77 |
| Intestines 2 | 1.61 | 0.00 | 0.00 | 0.00 | 0.00 | 0.00 | 0.00 | 0.00 | 2.48 | 0.66 |
| Intestines 3 | 1.20 | 0.00 | 0.00 | 0.00 | 0.00 | 0.00 | 0.00 | 0.00 | 2.04 | 0.52 |
| Intestines 4 | 0.99 | 0.00 | 0.00 | 0.00 | 0.00 | 0.00 | 0.00 | 0.45 | 0.00 | 0.00 |
| Intestines 5 | 1.04 | 0.00 | 0.00 | 0.00 | 0.00 | 0.00 | 0.00 | 0.00 | 1.55 | 0.00 |
| Kidney 1 | 0.00 | 0.00 | 0.00 | 0.00 | 0.00 | 0.00 | 0.00 | 33.41 | 0.00 | 0.00 |
| Kidney 2 | 0.00 | 0.00 | 0.00 | 0.00 | 0.00 | 0.00 | 0.00 | 31.00 | 0.00 | 0.00 |
| Kidney 3 | 0.00 | 0.00 | 0.00 | 0.00 | 0.00 | 0.00 | 0.00 | 39.37 | 0.00 | 0.00 |
| Kidney 4 | 0.00 | 0.00 | 0.00 | 0.00 | 0.00 | 0.00 | 0.00 | 25.53 | 0.00 | 0.00 |
| Kidney 5 | 0.00 | 0.00 | 0.00 | 0.76 | 0.00 | 0.00 | 0.00 | 38.61 | 0.00 | 0.00 |
| Liver 1 | 0.00 | 0.00 | 0.00 | 0.57 | 0.00 | 0.00 | 0.00 | 0.00 | 0.00 | 0.00 |
| Liver 2 | 0.00 | 0.00 | 0.00 | 0.10 | 0.00 | 0.00 | 0.00 | 0.00 | 1.51 | 0.00 |
| Liver 3 | 0.00 | 0.00 | 0.00 | 0.25 | 0.00 | 0.00 | 0.00 | 0.00 | 3.92 | 0.00 |
| Liver 4 | 0.00 | 0.00 | 0.00 | 0.24 | 0.00 | 0.00 | 0.00 | 0.00 | 3.32 | 0.00 |
| Liver 5 | 0.00 | 0.00 | 0.00 | 0.11 | 0.00 | 0.00 | 0.00 | 0.00 | 2.57 | 0.00 |
| Lung 1 | 0.00 | 0.00 | 0.80 | 0.00 | 0.00 | 0.00 | 0.00 | 0.00 | 40.20 | 0.00 |
| Lung 2 | 0.00 | 0.00 | 0.00 | 4.19 | 1.89 | 0.00 | 0.00 | 0.00 | 52.07 | 0.00 |
| Lung 3 | 0.00 | 0.00 | 0.00 | 3.41 | 1.92 | 2.53 | 6.47 | 0.00 | 72.74 | 0.00 |
| Lung 4 | 0.00 | 0.00 | 0.00 | 1.31 | 0.00 | 0.00 | 0.00 | 0.00 | 33.39 | 0.00 |
| Lung 5 | 0.00 | 0.00 | 0.00 | 0.00 | 0.00 | 0.00 | 0.00 | 0.00 | 34.19 | 0.00 |
| Muscle 1 | 0.00 | 0.00 | 0.00 | 0.00 | 0.00 | 0.00 | 0.00 | 0.00 | 14.88 | 0.00 |
| Muscle 2 | 0.00 | 0.00 | 0.00 | 0.00 | 0.00 | 0.00 | 0.00 | 0.00 | 17.66 | 0.00 |
| Muscle 3 | 0.00 | 0.00 | 0.00 | 0.00 | 0.00 | 0.00 | 0.00 | 0.00 | 6.61 | 0.00 |
| Muscle 4 | 0.00 | 0.00 | 0.00 | 0.00 | 0.00 | 0.00 | 0.00 | 0.00 | 2.78 | 0.00 |
| Muscle 5 | 0.00 | 0.00 | 0.00 | 0.00 | 0.00 | 0.00 | 0.00 | 0.00 | 12.36 | 0.00 |
| Ovary 1 | 0.00 | 0.00 | 0.00 | 0.00 | 0.00 | 0.00 | 0.00 | 0.00 | 39.87 | 0.00 |
| Ovary 2 | 0.00 | 0.00 | 0.00 | 0.40 | 0.16 | 0.00 | 0.00 | 0.00 | 8.74 | 0.00 |
| Ovary 3 | 0.00 | 0.00 | 0.00 | 0.72 | 0.35 | 0.00 | 0.00 | 0.00 | 17.65 | 0.00 |
| Ovary 4 | 0.00 | 0.00 | 0.00 | 0.36 | 0.20 | 0.00 | 0.00 | 0.00 | 27.49 | 0.00 |
| Ovary 5 | 0.00 | 0.00 | 0.00 | 0.63 | 0.38 | 0.00 | 0.00 | 0.00 | 36.28 | 0.00 |
| Pancreas 1 | 0.00 | 0.00 | 0.00 | 0.00 | 0.00 | 0.00 | 0.00 | 0.00 | 0.00 | 0.00 |
| Pancreas 2 | 0.00 | 0.00 | 0.00 | 0.00 | 0.00 | 0.00 | 0.00 | 0.00 | 1.15 | 0.00 |
| Pancreas 3 | 0.00 | 0.00 | 0.00 | 0.00 | 0.00 | 0.00 | 0.00 | 0.00 | 1.59 | 0.00 |
| Pancreas 4 | 0.00 | 0.00 | 0.00 | 0.00 | 0.00 | 0.00 | 0.00 | 0.00 | 3.38 | 0.00 |
| Pancreas 5 | 0.00 | 0.00 | 0.00 | 0.00 | 0.00 | 0.00 | 0.00 | 0.00 | 1.51 | 0.00 |
| Skin 1 | 0.00 | 0.00 | 0.00 | 0.00 | 0.00 | 0.00 | 0.00 | 0.00 | 32.83 | 0.00 |
| Skin 2 | 0.00 | 0.00 | 0.00 | 0.86 | 0.00 | 0.00 | 0.00 | 0.00 | 62.23 | 0.00 |
| Skin 3 | 0.00 | 0.00 | 0.00 | 0.78 | 0.00 | 0.00 | 0.00 | 0.00 | 51.87 | 0.00 |
| Skin 4 | 0.00 | 0.00 | 0.00 | 0.00 | 0.00 | 0.00 | 0.00 | 0.00 | 32.51 | 0.00 |
| Skin 5 | 0.00 | 0.00 | 0.00 | 0.00 | 0.00 | 0.00 | 0.00 | 0.00 | 32.55 | 0.00 |
| Spleen 1 | 0.00 | 0.00 | 0.00 | 0.00 | 0.00 | 0.00 | 1.66 | 0.00 | 5.80 | 0.00 |
| Spleen 2 | 0.00 | 0.00 | 0.00 | 1.17 | 2.32 | 1.22 | 3.80 | 0.00 | 14.52 | 0.00 |
| Spleen 3 | 0.00 | 0.00 | 0.00 | 0.00 | 0.00 | 0.00 | 1.40 | 0.00 | 8.32 | 0.00 |
| Spleen 4 | 0.00 | 0.00 | 0.00 | 0.00 | 0.00 | 0.00 | 1.86 | 0.00 | 9.28 | 0.00 |
| Spleen 5 | 0.00 | 0.00 | 0.00 | 0.00 | 0.00 | 0.00 | 0.00 | 0.00 | 15.33 | 0.00 |
| Stomach 1 | 0.00 | 0.00 | 0.00 | 0.00 | 0.00 | 0.00 | 0.00 | 0.00 | 0.00 | 0.00 |
| Stomach 2 | 0.00 | 0.00 | 0.00 | 0.00 | 0.00 | 0.00 | 0.00 | 0.00 | 6.85 | 0.00 |
| Stomach 3 | 0.53 | 0.00 | 0.00 | 0.00 | 0.00 | 0.00 | 0.00 | 0.00 | 0.00 | 0.00 |
| Stomach 4 | 0.00 | 0.00 | 0.00 | 0.00 | 0.00 | 0.00 | 0.00 | 0.00 | 11.56 | 0.00 |
| Stomach 5 | 0.00 | 0.00 | 0.00 | 0.22 | 0.00 | 0.00 | 0.00 | 0.00 | 8.24 | 0.00 |
| Testis 1 | 0.00 | 0.00 | 0.00 | 0.00 | 0.00 | 0.00 | 0.00 | 0.00 | 0.00 | 0.00 |
| Testis 2 | 0.00 | 0.00 | 0.00 | 0.00 | 0.00 | 0.00 | 0.00 | 0.00 | 2.01 | 0.00 |
| Testis 3 | 0.00 | 0.00 | 0.00 | 0.40 | 0.00 | 0.00 | 0.00 | 0.00 | 5.46 | 0.00 |
| Testis 4 | 0.00 | 0.00 | 0.00 | 0.44 | 0.00 | 0.00 | 0.00 | 0.00 | 4.58 | 0.00 |
| Testis 5 | 0.00 | 0.00 | 0.00 | 0.34 | 0.00 | 0.00 | 0.00 | 0.00 | 4.38 | 0.00 |
| Thyroid 1 | 0.00 | 0.00 | 0.00 | 1.78 | 0.00 | 0.00 | 0.00 | 0.00 | 0.00 | 0.00 |
| Thyroid 2 | 0.00 | 0.00 | 0.00 | 1.25 | 0.00 | 0.00 | 0.00 | 0.00 | 0.00 | 0.00 |
| Thyroid 3 | 0.00 | 0.00 | 0.00 | 1.18 | 0.00 | 0.00 | 0.00 | 0.00 | 0.00 | 0.00 |
| Thyroid 4 | 0.00 | 0.00 | 0.00 | 1.70 | 0.00 | 0.00 | 0.00 | 0.00 | 0.00 | 0.00 |
| Thyroid 5 | 0.00 | 0.00 | 0.00 | 1.43 | 0.00 | 0.00 | 0.00 | 0.00 | 0.00 | 0.00 |
| Uterus 1 | 0.00 | 0.00 | 0.00 | 0.00 | 0.00 | 0.00 | 0.00 | 0.00 | 50.16 | 0.00 |
| Uterus 2 | 0.00 | 0.00 | 0.00 | 0.00 | 0.00 | 0.00 | 0.00 | 0.00 | 18.42 | 0.00 |
| Uterus 3 | 0.00 | 0.00 | 0.00 | 0.79 | 2.35 | 0.00 | 0.00 | 0.00 | 14.08 | 0.00 |
| Uterus 4 | 0.00 | 0.00 | 0.00 | 0.00 | 0.00 | 0.00 | 0.00 | 0.00 | 24.74 | 0.00 |
| Uterus 5 | 0.00 | 0.00 | 0.33 | 0.00 | 0.00 | 0.00 | 0.00 | 0.00 | 27.56 | 0.00 |
| Serum 1 | 0.00 | 0.00 | 0.00 | 1.69 | 0.00 | 0.00 | 0.00 | 0.00 | 157.31 | 0.00 |
| Serum 2 | 0.00 | 1.45 | 0.00 | 1.43 | 0.00 | 0.00 | 0.00 | 0.00 | 151.68 | 0.00 |
| Serum 3 | 0.00 | 1.47 | 0.00 | 1.82 | 0.00 | 0.00 | 0.00 | 0.00 | 154.13 | 1.10 |
| Serum 4 | 0.00 | 2.06 | 0.00 | 2.47 | 0.00 | 0.00 | 0.00 | 0.00 | 204.58 | 0.92 |
| Serum 5 | 0.00 | 1.15 | 0.00 | 1.93 | 0.00 | 0.00 | 0.00 | 0.00 | 171.44 | 0.00 |
| Exosome 1 | 0.00 | 0.58 | 0.72 | 0.00 | 0.00 | 0.00 | 0.00 | 0.00 | 151.15 | 0.00 |
| Exosome 2 | 0.00 | 0.74 | 0.62 | 0.00 | 0.00 | 0.00 | 0.00 | 0.00 | 149.80 | 0.00 |
| Exosome 3 | 0.00 | 0.70 | 0.85 | 0.00 | 0.00 | 0.00 | 0.00 | 0.00 | 156.97 | 0.00 |
| Exosome 4 | 0.00 | 0.00 | 0.00 | 6.42 | 2.71 | 4.21 | 7.14 | 0.00 | 116.77 | 0.00 |
| Exosome 5 | 0.00 | 0.00 | 0.00 | 5.58 | 2.27 | 3.80 | 6.46 | 0.00 | 118.04 | 0.00 |

| Glycoform | **91** | **92** | **93** | **94** | **95** | **96** | **97** | **98** | **99** | **100** |
| --- | --- | --- | --- | --- | --- | --- | --- | --- | --- | --- |
| *m/z* | 2741.05 | 2766.09 | 2785.08 | 2827.09 | 2842.1 | 2843.09 | 2859.08 | 2887.11 | 3002.14 | 3034.13 |
| Abbreviation | H6N5A1 | H4N6D1A1 | H6N6D1 | H5N4D1A2 | H6N7 | H5N4D1A1G1 | H5N4D1G2 | H6N5D1A1 | H5N4A2G1 | H5N4G3 |
| Brain 1 | 0.00 | 0.76 | 0.00 | 0.00 | 0.00 | 0.00 | 0.00 | 0.00 | 0.00 | 0.00 |
| Brain 2 | 0.00 | 0.00 | 0.00 | 0.00 | 0.00 | 0.00 | 0.00 | 0.00 | 0.00 | 0.00 |
| Brain 3 | 0.00 | 0.09 | 0.00 | 0.00 | 0.00 | 0.00 | 0.00 | 0.00 | 0.00 | 0.00 |
| Brain 4 | 0.00 | 0.00 | 0.00 | 0.00 | 0.00 | 0.00 | 0.00 | 0.00 | 0.00 | 0.00 |
| Brain 5 | 0.00 | 0.09 | 0.00 | 0.09 | 0.00 | 0.00 | 0.00 | 0.00 | 0.00 | 0.00 |
| Femur 1 | 0.00 | 0.00 | 0.00 | 0.00 | 0.00 | 0.00 | 1.88 | 0.00 | 0.00 | 0.00 |
| Femur 2 | 0.00 | 0.00 | 0.00 | 1.18 | 0.00 | 0.00 | 2.69 | 0.00 | 0.00 | 0.00 |
| Femur 3 | 0.00 | 0.00 | 0.00 | 0.00 | 0.00 | 0.00 | 5.15 | 0.00 | 0.00 | 0.00 |
| Femur 4 | 0.00 | 0.00 | 0.00 | 0.00 | 0.00 | 0.00 | 3.98 | 0.00 | 0.00 | 0.00 |
| Femur 5 | 0.00 | 0.00 | 0.00 | 0.91 | 0.00 | 0.00 | 4.98 | 0.00 | 0.00 | 0.00 |
| Heart 1 | 0.00 | 0.00 | 0.00 | 0.70 | 0.00 | 0.00 | 0.76 | 0.00 | 0.00 | 0.00 |
| Heart 2 | 0.00 | 0.00 | 0.00 | 0.27 | 0.00 | 0.17 | 0.46 | 0.00 | 0.00 | 0.12 |
| Heart 3 | 0.00 | 0.00 | 0.00 | 0.36 | 0.00 | 0.24 | 0.77 | 0.00 | 0.00 | 0.00 |
| Heart 4 | 0.00 | 0.00 | 0.00 | 0.21 | 0.00 | 0.13 | 0.42 | 0.00 | 0.00 | 0.00 |
| Heart 5 | 0.00 | 0.00 | 0.00 | 0.00 | 0.00 | 0.00 | 0.43 | 0.00 | 0.00 | 0.00 |
| Intestines 1 | 0.00 | 0.00 | 0.00 | 0.00 | 0.00 | 0.00 | 0.00 | 0.00 | 0.00 | 0.00 |
| Intestines 2 | 0.00 | 0.00 | 0.38 | 0.00 | 0.00 | 0.33 | 0.93 | 0.00 | 0.00 | 0.00 |
| Intestines 3 | 0.00 | 0.00 | 0.00 | 0.00 | 0.00 | 0.00 | 0.00 | 0.00 | 0.00 | 0.00 |
| Intestines 4 | 0.00 | 0.00 | 0.00 | 0.00 | 0.00 | 0.00 | 0.00 | 0.00 | 0.00 | 0.00 |
| Intestines 5 | 0.00 | 0.00 | 0.74 | 0.31 | 0.00 | 0.00 | 0.40 | 0.00 | 0.00 | 0.00 |
| Kidney 1 | 0.00 | 0.00 | 0.00 | 0.00 | 0.00 | 0.00 | 0.87 | 0.00 | 0.00 | 0.00 |
| Kidney 2 | 0.00 | 0.00 | 0.00 | 0.00 | 0.00 | 0.00 | 0.00 | 0.00 | 0.00 | 0.00 |
| Kidney 3 | 0.00 | 0.00 | 0.00 | 0.00 | 0.00 | 0.00 | 0.00 | 0.00 | 0.00 | 0.00 |
| Kidney 4 | 0.00 | 0.00 | 0.00 | 0.00 | 0.00 | 0.00 | 0.00 | 0.00 | 0.00 | 0.00 |
| Kidney 5 | 0.00 | 0.00 | 0.00 | 0.00 | 0.00 | 0.00 | 0.73 | 0.00 | 0.00 | 0.00 |
| Liver 1 | 0.00 | 0.00 | 0.00 | 0.00 | 0.00 | 0.00 | 0.00 | 0.00 | 0.00 | 0.00 |
| Liver 2 | 0.00 | 0.00 | 0.00 | 0.00 | 0.00 | 0.00 | 0.00 | 0.00 | 0.00 | 0.00 |
| Liver 3 | 0.00 | 0.00 | 0.00 | 0.00 | 0.00 | 0.00 | 0.20 | 0.00 | 0.00 | 0.07 |
| Liver 4 | 0.00 | 0.00 | 0.00 | 0.00 | 0.00 | 0.00 | 0.11 | 0.00 | 0.00 | 0.00 |
| Liver 5 | 0.00 | 0.00 | 0.00 | 0.00 | 0.00 | 0.00 | 0.12 | 0.00 | 0.00 | 0.07 |
| Lung 1 | 1.28 | 0.00 | 0.00 | 1.87 | 0.00 | 3.63 | 9.00 | 0.00 | 0.00 | 0.00 |
| Lung 2 | 1.71 | 0.00 | 0.00 | 6.13 | 0.00 | 5.46 | 10.82 | 0.00 | 0.00 | 1.78 |
| Lung 3 | 1.40 | 0.00 | 0.00 | 9.23 | 0.00 | 6.36 | 20.15 | 1.73 | 0.00 | 1.50 |
| Lung 4 | 0.00 | 0.00 | 0.00 | 1.27 | 0.00 | 1.50 | 5.20 | 0.00 | 0.00 | 0.52 |
| Lung 5 | 0.00 | 0.00 | 0.00 | 3.06 | 0.00 | 4.11 | 9.17 | 0.00 | 0.00 | 0.82 |
| Muscle 1 | 0.00 | 0.00 | 0.00 | 1.12 | 0.00 | 0.00 | 2.14 | 0.00 | 0.00 | 0.00 |
| Muscle 2 | 0.00 | 0.00 | 0.00 | 0.00 | 0.00 | 0.00 | 3.23 | 0.00 | 0.00 | 0.00 |
| Muscle 3 | 0.00 | 0.00 | 0.00 | 0.00 | 0.00 | 0.00 | 2.31 | 0.00 | 0.00 | 0.00 |
| Muscle 4 | 0.00 | 0.00 | 0.00 | 0.00 | 0.00 | 0.00 | 0.61 | 0.00 | 0.00 | 0.00 |
| Muscle 5 | 0.00 | 0.00 | 0.00 | 1.06 | 0.00 | 0.00 | 3.11 | 0.00 | 0.00 | 0.00 |
| Ovary 1 | 0.00 | 0.00 | 0.00 | 1.54 | 0.00 | 1.52 | 11.20 | 0.00 | 0.00 | 0.00 |
| Ovary 2 | 0.00 | 0.00 | 0.00 | 1.22 | 0.00 | 1.28 | 0.00 | 0.00 | 0.00 | 0.00 |
| Ovary 3 | 0.00 | 0.00 | 0.00 | 1.36 | 0.00 | 1.56 | 7.17 | 0.00 | 0.00 | 0.00 |
| Ovary 4 | 0.00 | 0.00 | 0.00 | 0.83 | 0.00 | 1.16 | 10.44 | 0.00 | 0.00 | 0.70 |
| Ovary 5 | 0.00 | 0.00 | 0.00 | 1.10 | 0.00 | 1.37 | 11.41 | 0.00 | 0.00 | 0.72 |
| Pancreas 1 | 0.00 | 0.00 | 0.00 | 0.00 | 0.00 | 0.00 | 0.00 | 0.00 | 0.00 | 0.00 |
| Pancreas 2 | 0.00 | 0.00 | 0.00 | 0.00 | 0.00 | 0.00 | 0.00 | 0.00 | 0.00 | 0.00 |
| Pancreas 3 | 0.00 | 0.00 | 0.00 | 0.00 | 0.00 | 0.00 | 0.00 | 0.00 | 0.00 | 0.00 |
| Pancreas 4 | 0.00 | 0.00 | 0.00 | 0.00 | 0.00 | 0.00 | 0.00 | 0.00 | 0.00 | 0.00 |
| Pancreas 5 | 0.00 | 0.00 | 0.00 | 0.00 | 0.00 | 0.00 | 0.00 | 0.00 | 0.00 | 0.00 |
| Skin 1 | 0.78 | 0.00 | 0.00 | 0.79 | 0.00 | 0.94 | 13.40 | 0.00 | 0.00 | 0.54 |
| Skin 2 | 0.00 | 0.00 | 0.00 | 0.78 | 0.00 | 0.63 | 18.03 | 0.00 | 0.00 | 0.00 |
| Skin 3 | 0.00 | 0.00 | 0.00 | 0.95 | 0.00 | 0.93 | 16.29 | 0.00 | 0.00 | 0.00 |
| Skin 4 | 0.00 | 0.00 | 0.00 | 0.00 | 0.00 | 0.00 | 10.47 | 0.00 | 0.00 | 0.00 |
| Skin 5 | 0.00 | 0.00 | 0.00 | 0.91 | 0.00 | 0.80 | 9.47 | 0.00 | 0.00 | 0.00 |
| Spleen 1 | 0.00 | 0.00 | 0.00 | 0.79 | 0.00 | 0.88 | 6.61 | 0.00 | 0.00 | 0.00 |
| Spleen 2 | 0.00 | 0.00 | 0.00 | 2.53 | 0.00 | 2.27 | 14.00 | 0.00 | 0.00 | 0.00 |
| Spleen 3 | 0.00 | 0.00 | 0.00 | 1.75 | 0.00 | 1.00 | 8.25 | 0.00 | 0.00 | 0.00 |
| Spleen 4 | 0.00 | 0.00 | 0.00 | 1.85 | 0.00 | 1.63 | 13.70 | 0.00 | 0.00 | 0.00 |
| Spleen 5 | 0.00 | 0.00 | 0.00 | 2.36 | 0.00 | 2.04 | 13.68 | 0.00 | 0.00 | 0.00 |
| Stomach 1 | 0.00 | 0.00 | 0.00 | 0.00 | 0.00 | 0.00 | 0.00 | 0.00 | 0.00 | 0.00 |
| Stomach 2 | 0.00 | 0.00 | 0.00 | 0.00 | 0.00 | 0.00 | 0.51 | 0.00 | 0.00 | 0.00 |
| Stomach 3 | 0.00 | 0.00 | 0.00 | 0.00 | 0.00 | 0.00 | 0.00 | 0.00 | 0.00 | 0.00 |
| Stomach 4 | 0.00 | 0.00 | 0.00 | 0.41 | 0.00 | 0.38 | 1.04 | 0.00 | 0.00 | 0.00 |
| Stomach 5 | 0.00 | 0.00 | 0.00 | 0.19 | 0.17 | 0.00 | 0.54 | 0.00 | 0.00 | 0.00 |
| Testis 1 | 0.00 | 0.00 | 0.00 | 0.00 | 0.00 | 0.00 | 0.00 | 0.00 | 0.00 | 0.00 |
| Testis 2 | 0.00 | 0.00 | 0.00 | 0.79 | 0.00 | 0.00 | 0.00 | 0.00 | 0.00 | 0.00 |
| Testis 3 | 0.00 | 0.00 | 0.00 | 1.39 | 0.00 | 0.47 | 0.00 | 0.00 | 0.00 | 0.00 |
| Testis 4 | 0.00 | 0.00 | 0.00 | 1.24 | 0.00 | 0.43 | 0.61 | 0.00 | 0.00 | 0.00 |
| Testis 5 | 0.00 | 0.00 | 0.00 | 0.95 | 0.00 | 0.34 | 0.53 | 0.00 | 0.00 | 0.00 |
| Thyroid 1 | 0.00 | 0.00 | 0.00 | 224.04 | 0.00 | 0.00 | 0.00 | 36.60 | 0.00 | 0.00 |
| Thyroid 2 | 0.00 | 0.00 | 0.00 | 184.73 | 0.00 | 0.00 | 0.00 | 17.85 | 0.00 | 0.00 |
| Thyroid 3 | 0.00 | 0.00 | 0.00 | 171.21 | 0.00 | 0.00 | 0.00 | 18.70 | 0.00 | 0.00 |
| Thyroid 4 | 0.00 | 0.00 | 0.00 | 224.56 | 0.00 | 0.00 | 0.00 | 39.25 | 0.00 | 0.00 |
| Thyroid 5 | 0.00 | 0.00 | 0.00 | 204.69 | 0.00 | 0.00 | 0.00 | 26.26 | 0.00 | 0.00 |
| Uterus 1 | 0.00 | 0.00 | 0.00 | 1.61 | 1.51 | 0.00 | 8.76 | 0.00 | 0.00 | 0.00 |
| Uterus 2 | 0.00 | 0.00 | 0.00 | 3.23 | 0.00 | 2.61 | 7.82 | 0.00 | 0.00 | 0.00 |
| Uterus 3 | 0.00 | 0.00 | 0.00 | 3.44 | 0.00 | 2.88 | 6.10 | 0.00 | 0.00 | 0.00 |
| Uterus 4 | 0.00 | 0.00 | 0.00 | 1.16 | 0.00 | 1.06 | 6.67 | 0.00 | 0.00 | 0.00 |
| Uterus 5 | 0.00 | 0.00 | 0.00 | 1.59 | 0.00 | 1.16 | 9.60 | 0.00 | 0.00 | 0.00 |
| Serum 1 | 0.00 | 0.00 | 0.00 | 0.00 | 0.00 | 2.09 | 88.55 | 0.00 | 0.00 | 2.48 |
| Serum 2 | 4.03 | 0.00 | 1.10 | 1.04 | 0.00 | 1.87 | 82.62 | 1.66 | 0.00 | 3.02 |
| Serum 3 | 3.14 | 0.00 | 0.00 | 1.12 | 0.00 | 1.82 | 74.59 | 1.51 | 0.00 | 2.85 |
| Serum 4 | 3.50 | 0.00 | 0.00 | 0.00 | 0.00 | 0.00 | 108.19 | 1.60 | 1.24 | 3.18 |
| Serum 5 | 1.80 | 0.00 | 0.00 | 1.11 | 2.32 | 0.00 | 93.60 | 0.00 | 0.95 | 2.26 |
| Exosome 1 | 0.00 | 0.00 | 0.00 | 1.54 | 0.00 | 1.79 | 34.95 | 0.00 | 0.00 | 0.91 |
| Exosome 2 | 2.17 | 0.00 | 0.00 | 1.24 | 0.00 | 1.75 | 36.52 | 0.00 | 0.00 | 0.86 |
| Exosome 3 | 0.00 | 0.00 | 0.00 | 1.80 | 0.00 | 1.86 | 37.44 | 0.00 | 0.00 | 1.09 |
| Exosome 4 | 0.00 | 0.00 | 0.00 | 4.26 | 0.00 | 4.23 | 70.50 | 0.00 | 1.23 | 1.15 |
| Exosome 5 | 0.00 | 0.00 | 0.00 | 3.01 | 0.00 | 3.17 | 53.22 | 0.00 | 1.06 | 0.00 |

| Glycoform | **101** | **102** | **103** | **104** |
| --- | --- | --- | --- | --- |
| *m/z* | 3078.16 | 3192.22 | 3399.26 | 3497.33 |
| Abbreviation | H6N5G2 | H6N5D1A2 | H6N5G3 | H6N5D1A3 |
| Brain 1 | 0.00 | 0.00 | 0.00 | 0.00 |
| Brain 2 | 0.00 | 0.00 | 0.00 | 0.00 |
| Brain 3 | 0.00 | 0.00 | 0.00 | 0.00 |
| Brain 4 | 0.00 | 0.00 | 0.00 | 0.00 |
| Brain 5 | 0.00 | 0.00 | 0.00 | 0.00 |
| Femur 1 | 0.00 | 0.00 | 0.00 | 0.00 |
| Femur 2 | 0.00 | 0.00 | 0.00 | 0.00 |
| Femur 3 | 0.00 | 0.00 | 0.00 | 0.00 |
| Femur 4 | 0.00 | 0.00 | 0.00 | 0.00 |
| Femur 5 | 0.00 | 0.00 | 0.00 | 0.00 |
| Heart 1 | 0.00 | 0.00 | 0.00 | 0.00 |
| Heart 2 | 0.00 | 0.00 | 0.00 | 0.00 |
| Heart 3 | 0.00 | 0.00 | 0.00 | 0.00 |
| Heart 4 | 0.00 | 0.00 | 0.00 | 0.00 |
| Heart 5 | 0.00 | 0.00 | 0.00 | 0.00 |
| Intestines 1 | 0.00 | 0.00 | 0.00 | 0.00 |
| Intestines 2 | 0.00 | 0.00 | 0.00 | 0.00 |
| Intestines 3 | 0.00 | 0.00 | 0.00 | 0.00 |
| Intestines 4 | 0.00 | 0.00 | 0.00 | 0.00 |
| Intestines 5 | 0.00 | 0.00 | 0.00 | 0.00 |
| Kidney 1 | 0.00 | 0.00 | 0.00 | 0.00 |
| Kidney 2 | 0.00 | 0.00 | 0.00 | 0.00 |
| Kidney 3 | 0.00 | 0.00 | 0.00 | 0.00 |
| Kidney 4 | 0.00 | 0.00 | 0.00 | 0.00 |
| Kidney 5 | 0.00 | 0.00 | 0.00 | 0.00 |
| Liver 1 | 0.00 | 0.00 | 0.00 | 0.00 |
| Liver 2 | 0.00 | 0.00 | 0.00 | 0.00 |
| Liver 3 | 0.00 | 0.00 | 0.00 | 0.00 |
| Liver 4 | 0.00 | 0.00 | 0.00 | 0.00 |
| Liver 5 | 0.00 | 0.00 | 0.00 | 0.00 |
| Lung 1 | 0.00 | 0.00 | 0.00 | 0.00 |
| Lung 2 | 0.00 | 0.00 | 0.47 | 0.00 |
| Lung 3 | 0.00 | 0.00 | 0.00 | 0.00 |
| Lung 4 | 0.00 | 0.00 | 0.00 | 0.00 |
| Lung 5 | 0.00 | 0.00 | 0.00 | 0.00 |
| Muscle 1 | 0.00 | 0.00 | 0.00 | 0.00 |
| Muscle 2 | 0.00 | 0.00 | 0.00 | 0.00 |
| Muscle 3 | 0.00 | 0.00 | 0.00 | 0.00 |
| Muscle 4 | 0.00 | 0.00 | 0.00 | 0.00 |
| Muscle 5 | 0.00 | 0.00 | 0.00 | 0.00 |
| Ovary 1 | 0.00 | 0.00 | 0.00 | 0.00 |
| Ovary 2 | 0.00 | 0.00 | 0.00 | 0.00 |
| Ovary 3 | 0.00 | 0.00 | 0.21 | 0.00 |
| Ovary 4 | 0.00 | 0.00 | 0.00 | 0.00 |
| Ovary 5 | 0.00 | 0.00 | 1.90 | 0.00 |
| Pancreas 1 | 0.00 | 0.00 | 0.00 | 0.00 |
| Pancreas 2 | 0.00 | 0.00 | 0.00 | 0.00 |
| Pancreas 3 | 0.00 | 0.00 | 0.00 | 0.00 |
| Pancreas 4 | 0.00 | 0.00 | 0.00 | 0.00 |
| Pancreas 5 | 0.00 | 0.00 | 0.00 | 0.00 |
| Skin 1 | 0.78 | 0.00 | 0.59 | 0.00 |
| Skin 2 | 2.93 | 0.00 | 1.89 | 0.00 |
| Skin 3 | 2.23 | 0.00 | 0.00 | 0.00 |
| Skin 4 | 1.17 | 0.00 | 0.00 | 0.00 |
| Skin 5 | 1.09 | 0.00 | 0.00 | 0.00 |
| Spleen 1 | 0.00 | 0.00 | 0.00 | 0.00 |
| Spleen 2 | 0.00 | 0.00 | 0.00 | 0.00 |
| Spleen 3 | 0.00 | 0.00 | 0.00 | 0.00 |
| Spleen 4 | 0.00 | 0.00 | 0.00 | 0.00 |
| Spleen 5 | 0.00 | 0.00 | 0.00 | 0.00 |
| Stomach 1 | 0.00 | 0.00 | 0.00 | 0.00 |
| Stomach 2 | 0.00 | 0.00 | 0.00 | 0.00 |
| Stomach 3 | 0.00 | 0.00 | 0.00 | 0.00 |
| Stomach 4 | 0.00 | 0.00 | 0.00 | 0.00 |
| Stomach 5 | 0.00 | 0.00 | 0.00 | 0.00 |
| Testis 1 | 0.00 | 0.00 | 0.00 | 0.00 |
| Testis 2 | 0.00 | 0.00 | 0.00 | 0.00 |
| Testis 3 | 0.00 | 0.00 | 0.00 | 0.00 |
| Testis 4 | 0.00 | 0.00 | 0.00 | 0.00 |
| Testis 5 | 0.00 | 0.00 | 0.00 | 0.00 |
| Thyroid 1 | 0.00 | 8.57 | 0.00 | 1.96 |
| Thyroid 2 | 0.00 | 4.57 | 0.00 | 1.14 |
| Thyroid 3 | 0.00 | 3.77 | 0.00 | 0.77 |
| Thyroid 4 | 0.00 | 7.21 | 0.00 | 1.16 |
| Thyroid 5 | 0.00 | 5.77 | 0.00 | 1.32 |
| Uterus 1 | 0.00 | 0.00 | 1.01 | 0.00 |
| Uterus 2 | 0.00 | 0.00 | 0.00 | 0.00 |
| Uterus 3 | 0.00 | 0.00 | 0.00 | 0.00 |
| Uterus 4 | 0.00 | 0.00 | 0.00 | 0.00 |
| Uterus 5 | 0.00 | 0.00 | 0.00 | 0.00 |
| Serum 1 | 0.00 | 0.00 | 1.91 | 0.00 |
| Serum 2 | 0.00 | 0.00 | 2.07 | 0.00 |
| Serum 3 | 0.00 | 0.00 | 2.23 | 0.00 |
| Serum 4 | 0.00 | 0.00 | 2.79 | 0.00 |
| Serum 5 | 0.00 | 0.00 | 1.68 | 0.00 |
| Exosome 1 | 0.00 | 0.00 | 1.58 | 0.00 |
| Exosome 2 | 0.00 | 0.00 | 1.18 | 0.00 |
| Exosome 3 | 0.00 | 0.00 | 0.00 | 0.00 |
| Exosome 4 | 0.00 | 0.00 | 0.00 | 0.00 |
| Exosome 5 | 0.00 | 0.00 | 0.00 | 0.00 |

The amount of glycans were represented as pmol/100 μg protein.

**Table S3**. Averages for the expression levels of glycan groups in each organ in the glycotyping analysis (pmol/100 μg protein)

| Group | Paucimannose | high_man | hybrid | complex | Man | Hybrid | Glc | GlcNAc | Gal | GalNAc | Sia |
| --- | --- | --- | --- | --- | --- | --- | --- | --- | --- | --- | --- |
| Brain | 2.74 | 60.42 | 3.65 | 33.19 | 63.08 | 0.42 | 0.08 | 16.96 | 14.50 | 0.42 | 4.54 |
| Femur | 13.22 | 60.91 | 6.26 | 19.60 | 74.13 | 0.08 | 0.00 | 1.79 | 8.38 | 0.31 | 15.32 |
| Heart | 6.75 | 66.92 | 3.52 | 22.81 | 73.22 | 0.00 | 0.45 | 3.44 | 4.45 | 0.00 | 18.43 |
| Intestines | 9.48 | 47.69 | 3.73 | 39.10 | 57.17 | 0.42 | 0.00 | 12.96 | 25.65 | 0.40 | 3.40 |
| Kidney | 19.36 | 48.54 | 3.40 | 28.70 | 67.76 | 0.17 | 0.14 | 4.48 | 25.03 | 0.00 | 2.42 |
| Liver | 5.66 | 68.69 | 6.17 | 19.48 | 74.35 | 0.00 | 0.00 | 5.60 | 3.65 | 0.00 | 16.40 |
| Lung | 8.45 | 44.70 | 7.67 | 39.18 | 53.15 | 0.50 | 0.00 | 2.77 | 15.10 | 0.00 | 28.48 |
| Muscle | 11.44 | 51.41 | 8.60 | 28.55 | 62.86 | 0.00 | 0.00 | 1.26 | 7.07 | 0.00 | 28.82 |
| Ovary | 7.98 | 52.89 | 4.04 | 35.08 | 60.88 | 0.15 | 0.00 | 7.23 | 8.43 | 0.00 | 23.31 |
| Pancreas | 3.92 | 63.54 | 2.05 | 30.49 | 67.46 | 0.29 | 0.00 | 21.38 | 8.14 | 1.62 | 1.11 |
| Skin | 9.58 | 33.93 | 3.39 | 53.09 | 43.51 | 0.36 | 0.00 | 10.47 | 20.77 | 0.06 | 24.84 |
| Spleen | 32.29 | 41.27 | 7.03 | 19.42 | 73.41 | 0.28 | 0.15 | 2.81 | 5.01 | 0.00 | 18.34 |
| Stomach | 9.67 | 55.39 | 3.68 | 31.27 | 65.05 | 0.22 | 0.00 | 16.38 | 11.86 | 0.56 | 5.93 |
| Testis | 6.56 | 42.50 | 1.74 | 49.20 | 48.94 | 0.32 | 0.12 | 40.01 | 7.02 | 1.04 | 2.55 |
| Thyroid | 9.65 | 67.80 | 0.52 | 22.03 | 77.44 | 0.03 | 0.01 | 0.13 | 3.19 | 0.03 | 19.17 |
| Uterus | 10.51 | 54.93 | 3.27 | 31.29 | 65.37 | 0.32 | 0.07 | 6.74 | 10.18 | 0.02 | 17.29 |
| Serum | 0.00 | 3.21 | 2.86 | 93.93 | 3.21 | 0.04 | 0.00 | 17.32 | 13.77 | 0.00 | 65.65 |
| Exosome | 0.00 | 7.10 | 8.99 | 83.91 | 7.10 | 0.00 | 0.00 | 5.43 | 8.88 | 0.00 | 78.59 |

| Group | HexNAc x0 | HexNAc x1 | HexNAc x2 | HexNAc x3 | HexNAc x4 | HexNAc x5 |
| --- | --- | --- | --- | --- | --- | --- |
| Brain | 63.16 | 4.88 | 15.19 | 16.57 | 0.14 | 0.06 |
| Femur | 74.13 | 7.48 | 16.95 | 1.44 | 0.00 | 0.00 |
| Heart | 74.16 | 4.65 | 19.86 | 1.33 | 0.00 | 0.00 |
| Intestines | 58.35 | 10.00 | 14.92 | 15.05 | 1.38 | 0.30 |
| Kidney | 68.35 | 4.98 | 5.86 | 20.81 | 0.00 | 0.00 |
| Liver | 75.13 | 7.27 | 14.56 | 3.04 | 0.00 | 0.00 |
| Lung | 53.34 | 9.84 | 35.06 | 1.72 | 0.00 | 0.04 |
| Muscle | 62.86 | 8.60 | 25.56 | 2.99 | 0.00 | 0.00 |
| Ovary | 61.04 | 6.27 | 30.69 | 1.96 | 0.00 | 0.04 |
| Pancreas | 68.55 | 5.93 | 22.15 | 3.38 | 0.00 | 0.00 |
| Skin | 44.84 | 6.67 | 43.78 | 4.64 | 0.00 | 0.07 |
| Spleen | 73.99 | 10.36 | 14.29 | 1.36 | 0.00 | 0.00 |
| Stomach | 68.60 | 10.77 | 14.82 | 4.93 | 0.86 | 0.02 |
| Testis | 50.26 | 6.66 | 25.01 | 17.31 | 0.73 | 0.02 |
| Thyroid | 77.45 | 1.98 | 18.70 | 1.87 | 0.00 | 0.00 |
| Uterus | 65.89 | 6.55 | 25.43 | 1.97 | 0.00 | 0.16 |
| Serum | 3.21 | 3.34 | 91.42 | 1.81 | 0.12 | 0.10 |
| Exosome | 7.10 | 8.90 | 82.34 | 1.26 | 0.00 | 0.40 |

| Group | Sia x0 | Sia x1 | Sia x2 | Sia x3 | Fuc x0 | Fuc x1 | Fuc x2 | Fuc x3 |
| --- | --- | --- | --- | --- | --- | --- | --- | --- |
| Brain | 95.46 | 4.47 | 0.07 | 0.00 | 66.11 | 24.20 | 7.28 | 2.41 |
| Femur | 84.68 | 5.89 | 9.43 | 0.00 | 82.35 | 17.39 | 0.25 | 0.00 |
| Heart | 81.57 | 5.40 | 12.98 | 0.06 | 86.80 | 12.89 | 0.00 | 0.31 |
| Intestines | 96.60 | 2.81 | 0.59 | 0.00 | 67.68 | 31.20 | 0.90 | 0.22 |
| Kidney | 97.58 | 2.26 | 0.15 | 0.00 | 64.57 | 17.94 | 4.79 | 12.70 |
| Liver | 83.60 | 9.35 | 6.97 | 0.08 | 92.08 | 4.60 | 1.87 | 1.44 |
| Lung | 71.52 | 10.11 | 18.11 | 0.26 | 73.58 | 25.92 | 0.38 | 0.11 |
| Muscle | 71.18 | 12.82 | 16.00 | 0.00 | 84.32 | 14.06 | 1.62 | 0.00 |
| Ovary | 76.69 | 8.13 | 14.89 | 0.29 | 75.61 | 24.15 | 0.23 | 0.00 |
| Pancreas | 98.89 | 0.86 | 0.25 | 0.00 | 72.92 | 26.13 | 0.84 | 0.11 |
| Skin | 75.16 | 10.57 | 14.08 | 0.20 | 64.93 | 34.77 | 0.20 | 0.10 |
| Spleen | 81.66 | 9.90 | 8.44 | 0.00 | 70.12 | 29.44 | 0.44 | 0.00 |
| Stomach | 94.07 | 3.58 | 2.35 | 0.00 | 77.80 | 20.30 | 1.03 | 0.87 |
| Testis | 97.45 | 1.71 | 0.84 | 0.00 | 55.05 | 44.95 | 0.00 | 0.00 |
| Thyroid | 80.83 | 11.56 | 7.56 | 0.05 | 77.66 | 21.87 | 0.00 | 0.47 |
| Uterus | 82.71 | 6.49 | 10.72 | 0.08 | 76.66 | 23.29 | 0.05 | 0.00 |
| Serum | 34.35 | 8.29 | 56.19 | 1.18 | 47.61 | 51.93 | 0.18 | 0.27 |
| Exosome | 21.41 | 21.12 | 56.93 | 0.53 | 64.36 | 34.97 | 0.53 | 0.13 |

Averages of the glycan expression levels (n=5) were represented as pmol/100 μg protein.

**Table S4**. Expression levels of glycoforms identified in DBA/2Crslc and MRL-*lpr*/*lpr* mice.

| Peak No. | **1** | **2** | **3** | **4** | **5** | **6** | **7** | **8** | **9** | **10** |
| --- | --- | --- | --- | --- | --- | --- | --- | --- | --- | --- |
| m/z | 1340.55 | 1486.60 | 1502.60 | 1543.63 | 1648.66 | 1664.65 | 1689.68 | 1705.68 | 1746.70 | 1810.71 |
| Abbreviation | H3N2 | H3N2D1 | H4N2 | H3N3 | H4N2D1 | H5N2 | H3N3D1 | H4N3 | H3N4 | H5N2D1 |
| DBA.1 | 4.97 | 4.82 | 4.57 | 0.94 | 0.00 | 61.70 | 2.10 | 2.14 | 0.00 | 0.00 |
| DBA.2 | 3.32 | 3.03 | 3.14 | 0.00 | 0.00 | 42.13 | 1.40 | 0.00 | 0.00 | 0.00 |
| DBA.3 | 7.57 | 8.30 | 7.07 | 1.58 | 0.00 | 82.31 | 4.05 | 3.96 | 0.00 | 0.00 |
| DBA.4 | 6.56 | 6.69 | 5.77 | 1.01 | 0.00 | 78.32 | 2.47 | 2.40 | 0.00 | 0.00 |
| DBA.5 | 9.46 | 8.66 | 8.82 | 1.47 | 0.00 | 97.33 | 3.78 | 3.77 | 0.00 | 0.00 |
| MRL.1 | 7.11 | 6.48 | 9.55 | 2.33 | 0.00 | 140.54 | 5.82 | 0.00 | 0.00 | 0.00 |
| MRL.2 | 3.32 | 2.79 | 4.39 | 0.00 | 0.00 | 67.61 | 2.07 | 2.05 | 0.00 | 0.00 |
| MRL.3 | 3.86 | 2.41 | 5.02 | 0.00 | 0.00 | 81.49 | 0.00 | 2.25 | 0.00 | 0.00 |
| MRL.4 | 6.12 | 5.12 | 8.56 | 2.14 | 0.00 | 125.01 | 5.44 | 5.89 | 0.00 | 0.00 |
| MRL.5 | 17.79 | 12.21 | 18.34 | 0.00 | 0.00 | 220.08 | 9.46 | 10.34 | 0.00 | 0.00 |

| Peak No. | **11** | **12** | **13** | **14** | **15** | **16** | **17** | **18** | **19** | **20** |
| --- | --- | --- | --- | --- | --- | --- | --- | --- | --- | --- |
| m/z | 1826.71 | 1851.74 | 1867.73 | 1892.76 | 1908.76 | 1949.78 | 1988.76 | 1997.79 | 2010.79 | 2013.79 |
| Abbreviation | H6N2 | H4N3D1 | H5N3 | H3N4D1 | H4N4 | H3N5 | H7N2 | H4N3D2 | H4N3A1 | H5N3D1 |
| DBA.1 | 33.17 | 2.00 | 5.75 | 3.07 | 0.00 | 0.00 | 19.18 | 0.00 | 4.63 | 0.00 |
| DBA.2 | 20.93 | 0.00 | 5.13 | 0.00 | 0.00 | 0.00 | 12.41 | 0.00 | 4.21 | 0.00 |
| DBA.3 | 50.58 | 4.04 | 11.84 | 5.92 | 4.08 | 0.00 | 32.36 | 0.00 | 6.84 | 3.73 |
| DBA.4 | 40.07 | 2.19 | 6.19 | 3.37 | 1.92 | 0.00 | 24.13 | 0.00 | 4.81 | 2.24 |
| DBA.5 | 61.13 | 3.17 | 12.28 | 5.63 | 2.76 | 0.00 | 40.34 | 0.00 | 7.22 | 3.31 |
| MRL.1 | 74.35 | 5.63 | 11.37 | 15.51 | 0.00 | 0.00 | 52.48 | 0.00 | 9.87 | 5.64 |
| MRL.2 | 40.07 | 0.00 | 6.10 | 4.39 | 0.00 | 0.00 | 27.53 | 0.00 | 4.87 | 0.00 |
| MRL.3 | 42.11 | 0.00 | 6.60 | 4.25 | 0.00 | 0.00 | 30.59 | 0.00 | 5.39 | 0.00 |
| MRL.4 | 65.77 | 6.98 | 13.68 | 13.38 | 0.00 | 0.00 | 53.06 | 0.00 | 6.74 | 6.38 |
| MRL.5 | 95.52 | 8.64 | 14.10 | 18.88 | 0.00 | 0.00 | 62.64 | 0.00 | 0.00 | 0.00 |

| Peak No. | **21** | **22** | **23** | **24** | **25** | **26** | **27** | **28** | **29** | **30** |
| --- | --- | --- | --- | --- | --- | --- | --- | --- | --- | --- |
| m/z | 2026.79 | 2029.78 | 2054.82 | 2070.81 | 2095.84 | 2111.84 | 2150.81 | 2156.85 | 2172.84 | 2175.84 |
| Abbreviation | H4N3G1 | H6N3 | H4N4D1 | H5N4 | H3N5D1 | H4N5 | H8N2 | H4N3D1A1 | H5N3A1 | H6N3D1 |
| DBA.1 | 2.23 | 12.25 | 0.00 | 6.92 | 2.38 | 0.00 | 26.39 | 4.43 | 40.03 | 0.00 |
| DBA.2 | 0.00 | 8.55 | 0.00 | 0.00 | 0.00 | 0.00 | 18.09 | 3.67 | 47.60 | 0.00 |
| DBA.3 | 0.00 | 20.26 | 0.00 | 15.55 | 0.00 | 0.00 | 44.18 | 0.00 | 52.93 | 0.00 |
| DBA.4 | 2.16 | 15.83 | 4.21 | 7.10 | 1.70 | 0.00 | 31.46 | 3.58 | 36.80 | 0.00 |
| DBA.5 | 2.67 | 22.16 | 6.50 | 8.92 | 2.39 | 0.00 | 54.85 | 4.14 | 52.13 | 0.00 |
| MRL.1 | 6.05 | 18.61 | 16.26 | 16.29 | 5.71 | 0.00 | 71.16 | 8.51 | 44.19 | 0.00 |
| MRL.2 | 0.00 | 6.00 | 5.06 | 0.00 | 0.00 | 0.00 | 41.81 | 5.91 | 35.49 | 0.00 |
| MRL.3 | 3.00 | 8.80 | 5.44 | 16.72 | 0.00 | 0.00 | 44.31 | 7.57 | 41.94 | 0.00 |
| MRL.4 | 4.31 | 18.76 | 16.18 | 24.95 | 5.07 | 0.00 | 71.72 | 6.25 | 42.73 | 0.00 |
| MRL.5 | 0.00 | 19.51 | 18.98 | 30.84 | 0.00 | 0.00 | 76.83 | 10.77 | 37.18 | 0.00 |

| Peak No. | **31** | **32** | **33** | **34** | **35** | **36** | **37** | **38** | **39** | **40** |
| --- | --- | --- | --- | --- | --- | --- | --- | --- | --- | --- |
| m/z | 2185.84 | 2188.84 | 2200.87 | 2213.87 | 2216.87 | 2229.86 | 2232.86 | 2241.90 | 2257.90 | 2273.89 |
| Abbreviation | H3N3G2 | H5N3G1 | H4N4D2 | H4N4A1 | H5N4D1 | H4N4G1 | H6N4 | H3N5D2 | H4N5D1 | H5N5 |
| DBA.1 | 0.00 | 0.00 | 0.00 | 0.00 | 46.73 | 0.00 | 0.00 | 0.00 | 3.52 | 0.00 |
| DBA.2 | 0.00 | 0.00 | 0.00 | 2.32 | 41.07 | 0.00 | 0.00 | 0.00 | 2.28 | 0.00 |
| DBA.3 | 0.00 | 0.00 | 0.00 | 0.00 | 82.66 | 0.00 | 0.00 | 0.00 | 0.00 | 0.00 |
| DBA.4 | 0.00 | 0.00 | 0.00 | 0.00 | 53.63 | 0.00 | 0.00 | 0.00 | 3.09 | 0.00 |
| DBA.5 | 0.00 | 1.74 | 0.00 | 0.00 | 82.49 | 0.00 | 0.00 | 0.00 | 0.00 | 0.00 |
| MRL.1 | 0.00 | 0.00 | 0.00 | 6.70 | 94.44 | 0.00 | 0.00 | 0.00 | 0.00 | 0.00 |
| MRL.2 | 0.00 | 0.00 | 0.00 | 0.00 | 43.56 | 0.00 | 0.00 | 0.00 | 0.00 | 0.00 |
| MRL.3 | 0.00 | 0.00 | 0.00 | 0.00 | 47.37 | 0.00 | 0.00 | 0.00 | 0.00 | 0.00 |
| MRL.4 | 0.00 | 6.07 | 0.00 | 0.00 | 153.78 | 0.00 | 0.00 | 0.00 | 0.00 | 0.00 |
| MRL.5 | 0.00 | 0.00 | 0.00 | 0.00 | 123.20 | 0.00 | 0.00 | 0.00 | 0.00 | 0.00 |

| Peak No. | **41** | **42** | **43** | **44** | **45** | **46** | **47** | **48** | **49** | **50** |
| --- | --- | --- | --- | --- | --- | --- | --- | --- | --- | --- |
| m/z | 2298.92 | 2312.86 | 2318.90 | 2334.90 | 2350.89 | 2359.93 | 2362.93 | 2375.92 | 2378.92 | 2391.92 |
| Abbreviation | H3N6D1 | H9N2 | H5N3D1A1 | H6N3A1 | H6N3G1 | H4N4D1A1 | H5N4D2 | H5N4A1 | H6N4D1 | H5N4G1 |
| DBA.1 | 0.00 | 27.81 | 0.00 | 2.80 | 0.00 | 0.00 | 0.00 | 5.15 | 0.00 | 7.89 |
| DBA.2 | 0.00 | 18.35 | 0.00 | 2.07 | 0.00 | 0.00 | 0.00 | 3.02 | 0.00 | 5.20 |
| DBA.3 | 0.00 | 45.87 | 0.00 | 4.04 | 0.00 | 0.00 | 0.00 | 5.79 | 3.54 | 7.42 |
| DBA.4 | 0.00 | 32.72 | 0.00 | 2.45 | 1.66 | 0.00 | 0.00 | 4.33 | 0.00 | 5.45 |
| DBA.5 | 0.00 | 58.72 | 0.00 | 3.34 | 2.30 | 1.98 | 0.00 | 5.95 | 2.69 | 7.76 |
| MRL.1 | 0.00 | 82.56 | 0.00 | 8.29 | 5.31 | 5.08 | 0.00 | 16.48 | 5.54 | 22.96 |
| MRL.2 | 0.00 | 47.09 | 0.00 | 3.51 | 0.00 | 0.00 | 0.00 | 4.61 | 0.00 | 8.69 |
| MRL.3 | 0.00 | 50.72 | 0.00 | 3.97 | 0.00 | 0.00 | 0.00 | 6.40 | 0.00 | 14.37 |
| MRL.4 | 0.00 | 83.76 | 0.00 | 6.28 | 0.00 | 0.00 | 0.00 | 0.00 | 0.00 | 42.47 |
| MRL.5 | 0.00 | 80.70 | 0.00 | 0.00 | 0.00 | 0.00 | 0.00 | 13.89 | 11.27 | 36.54 |

| Peak No. | **51** | **52** | **53** | **54** | **55** | **56** | **57** | **58** | **60** |
| --- | --- | --- | --- | --- | --- | --- | --- | --- | --- |
| m/z | 2403.95 | 2416.95 | 2419.95 | 2435.94 | 2444.98 | 2445.96 | 2460.98 | 2474.92 | 2489.99 |
| Abbreviation | H4N5D2 | H4N5A1 | H5N5D1 | H6N5 | H3N6D2 | H3N3D2A2 | H4N6D1 | H10N2 | H3N4D3A1 |
| DBA.1 | 0.00 | 0.00 | 2.74 | 12.37 | 0.00 | 0.00 | 0.00 | 0.00 | 0.00 |
| DBA.2 | 0.00 | 0.00 | 0.00 | 12.12 | 0.00 | 0.00 | 0.00 | 0.00 | 0.00 |
| DBA.3 | 0.00 | 0.00 | 0.00 | 15.40 | 0.00 | 0.00 | 0.00 | 16.17 | 0.00 |
| DBA.4 | 0.00 | 0.00 | 0.00 | 13.73 | 0.00 | 0.00 | 0.00 | 7.58 | 0.00 |
| DBA.5 | 0.00 | 0.00 | 4.45 | 15.54 | 0.00 | 0.00 | 0.00 | 0.00 | 2.61 |
| MRL.1 | 0.00 | 0.00 | 0.00 | 15.09 | 0.00 | 0.00 | 0.00 | 4.01 | 0.00 |
| MRL.2 | 0.00 | 0.00 | 0.00 | 5.55 | 0.00 | 0.00 | 0.00 | 9.31 | 0.00 |
| MRL.3 | 0.00 | 0.00 | 0.00 | 11.69 | 0.00 | 0.00 | 0.00 | 10.34 | 0.00 |
| MRL.4 | 0.00 | 0.00 | 11.47 | 18.33 | 0.00 | 0.00 | 0.00 | 0.00 | 8.81 |
| MRL.5 | 0.00 | 0.00 | 0.00 | 17.80 | 0.00 | 0.00 | 0.00 | 0.00 | 0.00 |

| Peak No. | **61** | **62** | **63** | **64** | **65** | **66** | **67** | **68** | **69** | **70** |
| --- | --- | --- | --- | --- | --- | --- | --- | --- | --- | --- |
| m/z | 2499.95 | 2502.00 | 2505.99 | 2508.99 | 2518.00 | 2518.98 | 2521.98 | 2524.98 | 2534.98 | 2537.97 |
| Abbreviation | H8N3D1 | H3N7D1 | H4N4D2A1 | H5N4D3 | H4N7 | H4N4A2 | H5N4D1A1 | H6N4D2 | H3N4D1G2 | H5N4D1G1 |
| DBA.1 | 23.75 | 0.00 | 0.00 | 0.00 | 0.00 | 0.00 | 0.00 | 0.00 | 0.00 | 19.42 |
| DBA.2 | 28.41 | 0.00 | 12.64 | 0.00 | 0.00 | 0.00 | 0.00 | 0.00 | 0.00 | 8.45 |
| DBA.3 | 29.85 | 0.00 | 16.13 | 0.00 | 0.00 | 0.00 | 0.00 | 0.00 | 0.00 | 0.00 |
| DBA.4 | 11.19 | 0.00 | 0.00 | 0.00 | 0.00 | 0.00 | 1.73 | 0.00 | 0.00 | 15.56 |
| DBA.5 | 0.00 | 0.00 | 19.56 | 0.00 | 0.00 | 0.00 | 0.00 | 0.00 | 0.00 | 14.74 |
| MRL.1 | 16.31 | 0.00 | 21.61 | 0.00 | 0.00 | 10.19 | 0.00 | 0.00 | 0.00 | 42.10 |
| MRL.2 | 18.78 | 0.00 | 7.70 | 0.00 | 2.43 | 0.00 | 0.00 | 0.00 | 0.00 | 12.27 |
| MRL.3 | 15.91 | 0.00 | 14.78 | 0.00 | 2.06 | 0.00 | 0.00 | 0.00 | 0.00 | 15.54 |
| MRL.4 | 0.00 | 0.00 | 19.93 | 0.00 | 0.00 | 17.96 | 50.52 | 0.00 | 10.05 | 49.14 |
| MRL.5 | 15.02 | 0.00 | 18.00 | 0.00 | 0.00 | 12.47 | 44.40 | 0.00 | 0.00 | 43.26 |

| Peak No. | **71** | **72** | **73** | **74** | **75** | **76** | **77** | **78** | **79** | **80** |
| --- | --- | --- | --- | --- | --- | --- | --- | --- | --- | --- |
| m/z | 2540.97 | 2550.01 | 2553.97 | 2563.01 | 2566.01 | 2579.00 | 2582.00 | 2588.99 | 2591.04 | 2607.03 |
| Abbreviation | H7N4D1 | H4N5D3 | H6N4G1 | H4N5D1A1 | H5N5D2 | H5N5A1 | H6N5D1 | H8N2D3 | H3N6D3 | H4N6D2 |
| DBA.1 | 0.00 | 0.00 | 0.00 | 0.00 | 0.00 | 0.00 | 0.00 | 0.00 | 0.00 | 0.00 |
| DBA.2 | 0.00 | 0.00 | 0.00 | 0.00 | 0.00 | 0.00 | 0.00 | 0.00 | 0.00 | 0.00 |
| DBA.3 | 0.00 | 0.00 | 7.00 | 0.00 | 0.00 | 0.00 | 4.61 | 0.00 | 0.00 | 0.00 |
| DBA.4 | 2.29 | 0.00 | 0.00 | 0.00 | 0.00 | 0.00 | 0.00 | 0.00 | 0.00 | 0.00 |
| DBA.5 | 8.72 | 0.00 | 5.42 | 0.00 | 6.24 | 0.00 | 4.85 | 0.00 | 0.00 | 0.00 |
| MRL.1 | 0.00 | 0.00 | 18.94 | 0.00 | 0.00 | 0.00 | 0.00 | 0.00 | 0.00 | 0.00 |
| MRL.2 | 5.59 | 0.00 | 5.21 | 0.00 | 3.19 | 0.00 | 0.00 | 0.00 | 0.00 | 0.00 |
| MRL.3 | 5.54 | 0.00 | 8.43 | 0.00 | 0.00 | 0.00 | 0.00 | 0.00 | 0.00 | 0.00 |
| MRL.4 | 16.47 | 0.00 | 0.00 | 0.00 | 0.00 | 0.00 | 15.48 | 0.00 | 0.00 | 0.00 |
| MRL.5 | 0.00 | 0.00 | 12.99 | 0.00 | 0.00 | 0.00 | 0.00 | 0.00 | 0.00 | 0.00 |

| Peak No. | **81** | **82** | **83** | **84** | **85** | **86** | **87** | **88** | **89** | **90** |
| --- | --- | --- | --- | --- | --- | --- | --- | --- | --- | --- |
| m/z | 2623.03 | 2671.04 | 2680.05 | 2681.03 | 2684.03 | 2697.03 | 2700.03 | 2712.06 | 2713.02 | 2725.06 |
| Abbreviation | H5N6D1 | H6N4D3 | H5N7 | H5N4A2 | H6N4D1A1 | H5N4A1G1 | H7N4A1 | H5N5D3 | H5N4G2 | H5N5D1A1 |
| DBA.1 | 0.00 | 0.00 | 3.32 | 0.00 | 0.00 | 0.00 | 0.00 | 0.00 | 8.36 | 0.00 |
| DBA.2 | 0.00 | 0.00 | 1.63 | 0.00 | 0.00 | 0.00 | 0.00 | 0.00 | 29.43 | 0.00 |
| DBA.3 | 0.00 | 0.00 | 3.52 | 0.00 | 0.00 | 5.02 | 11.41 | 0.00 | 44.75 | 0.00 |
| DBA.4 | 0.00 | 0.00 | 2.72 | 0.00 | 0.00 | 0.00 | 0.00 | 0.00 | 53.00 | 0.00 |
| DBA.5 | 0.00 | 0.00 | 0.00 | 5.08 | 10.10 | 0.00 | 0.00 | 0.00 | 53.67 | 0.00 |
| MRL.1 | 0.00 | 0.00 | 8.62 | 0.00 | 0.00 | 14.88 | 34.05 | 0.00 | 115.14 | 0.00 |
| MRL.2 | 0.00 | 0.00 | 2.23 | 0.00 | 0.00 | 0.00 | 0.00 | 0.00 | 5.77 | 0.00 |
| MRL.3 | 0.00 | 0.00 | 0.00 | 0.00 | 0.00 | 0.00 | 0.00 | 0.00 | 66.54 | 0.00 |
| MRL.4 | 0.00 | 0.00 | 5.41 | 0.00 | 0.00 | 0.00 | 0.00 | 0.00 | 107.11 | 0.00 |
| MRL.5 | 0.00 | 0.00 | 0.00 | 0.00 | 0.00 | 0.00 | 0.00 | 0.00 | 79.46 | 0.00 |

| Peak No. | **91** | **92** | **93** | **94** | **95** | **96** | **97** | **98** | **99** | **100** |
| --- | --- | --- | --- | --- | --- | --- | --- | --- | --- | --- |
| m/z | 2741.05 | 2766.09 | 2785.08 | 2827.09 | 2842.10 | 2843.09 | 2859.08 | 2887.11 | 3002.14 | 3034.13 |
| Abbreviation | H6N5A1 | H4N6D1A1 | H6N6D1 | H5N4D1A2 | H6N7 | H5N4D1A1G1 | H5N4D1G2 | H6N5D1A1 | H5N4A2G1 | H5N4G3 |
| DBA.1 | 2.75 | 0.00 | 0.00 | 13.89 | 0.00 | 13.86 | 19.34 | 0.00 | 0.00 | 2.65 |
| DBA.2 | 0.00 | 0.00 | 0.00 | 6.38 | 0.00 | 6.42 | 8.58 | 0.00 | 0.00 | 0.00 |
| DBA.3 | 0.00 | 0.00 | 0.00 | 13.21 | 0.00 | 13.71 | 19.21 | 0.00 | 0.00 | 2.27 |
| DBA.4 | 2.00 | 0.00 | 0.00 | 12.32 | 0.00 | 11.31 | 17.52 | 0.00 | 0.00 | 1.97 |
| DBA.5 | 0.00 | 0.00 | 0.00 | 12.90 | 0.00 | 13.38 | 19.02 | 0.00 | 0.00 | 2.37 |
| MRL.1 | 14.85 | 0.00 | 0.00 | 32.30 | 0.00 | 36.68 | 56.05 | 0.00 | 0.00 | 3.90 |
| MRL.2 | 3.14 | 0.00 | 0.00 | 8.37 | 0.00 | 10.55 | 19.18 | 0.00 | 0.00 | 0.00 |
| MRL.3 | 0.00 | 0.00 | 0.00 | 9.01 | 0.00 | 11.71 | 21.15 | 0.00 | 0.00 | 0.00 |
| MRL.4 | 8.39 | 0.00 | 0.00 | 23.20 | 0.00 | 28.69 | 49.52 | 0.00 | 0.00 | 4.29 |
| MRL.5 | 0.00 | 0.00 | 0.00 | 14.45 | 0.00 | 17.84 | 31.68 | 0.00 | 0.00 | 0.00 |

| Peak No. | **101** | **102** | **103** | **104** |
| --- | --- | --- | --- | --- |
| m/z | 3078.16 | 3192.22 | 3399.26 | 3497.33 |
| Abbreviation | H6N5G2 | H6N5D1A2 | H6N5G3 | H6N5D1A3 |
| DBA.1 | 0.00 | 0.00 | 2.65 | 0.00 |
| DBA.2 | 0.00 | 0.00 | 0.00 | 0.00 |
| DBA.3 | 0.00 | 0.00 | 2.03 | 0.00 |
| DBA.4 | 0.00 | 0.00 | 1.64 | 0.00 |
| DBA.5 | 0.00 | 0.00 | 2.60 | 0.00 |
| MRL.1 | 0.00 | 0.00 | 4.98 | 0.00 |
| MRL.2 | 0.00 | 0.00 | 0.00 | 0.00 |
| MRL.3 | 0.00 | 0.00 | 0.00 | 0.00 |
| MRL.4 | 0.00 | 0.00 | 5.51 | 0.00 |
| MRL.5 | 0.00 | 0.00 | 0.00 | 0.00 |

Averages of the glycan expression levels were represented as pmol/100 μg protein.

**Table S5**. The confusion matrixes by (a) decision tree in 1007 tests, (b) neural network in 1006 tests, (c) random forest in 1006 tests, and (d) SVM in 1006 tests.

**a**

|  | Reference | |  |  |  |  |  |  |  |  |  |  |  |  |  |  |  |  |
| --- | --- | --- | --- | --- | --- | --- | --- | --- | --- | --- | --- | --- | --- | --- | --- | --- | --- | --- |
|  | Brain | Exosome | Femur | Heart | Intestines | Kidney | Liver | Lung | Muscle | Ovary | Pancreas | Serum | Skin | Spleen | Stomach | Testis | Thyroid | Uterus |
| Brain | 777 | 0 | 2 | 16 | 0 | 0 | 12 | 3 | 0 | 11 | 0 | 0 | 0 | 1 | 202 | 1 | 0 | 0 |
| Exosome | 4 | 806 | 0 | 2 | 0 | 0 | 1 | 1 | 0 | 0 | 0 | 160 | 0 | 0 | 0 | 0 | 0 | 0 |
| Femur | 9 | 0 | 681 | 107 | 0 | 12 | 0 | 96 | 14 | 309 | 8 | 0 | 1 | 30 | 1 | 12 | 2 | 24 |
| Heart | 20 | 0 | 62 | 580 | 0 | 13 | 125 | 31 | 104 | 53 | 27 | 0 | 26 | 17 | 3 | 4 | 0 | 27 |
| Intestines | 5 | 0 | 0 | 1 | 614 | 210 | 0 | 0 | 0 | 0 | 11 | 0 | 1 | 0 | 177 | 3 | 1 | 1 |
| Kidney | 14 | 0 | 6 | 20 | 217 | 750 | 3 | 0 | 0 | 31 | 0 | 0 | 4 | 4 | 34 | 0 | 0 | 4 |
| Liver | 5 | 0 | 0 | 52 | 0 | 0 | 652 | 19 | 54 | 65 | 22 | 0 | 3 | 13 | 2 | 4 | 0 | 46 |
| Lung | 0 | 0 | 13 | 6 | 0 | 0 | 0 | 454 | 0 | 20 | 0 | 0 | 35 | 10 | 0 | 0 | 0 | 110 |
| Muscle | 2 | 0 | 69 | 99 | 0 | 0 | 74 | 9 | 752 | 2 | 15 | 0 | 8 | 2 | 0 | 0 | 0 | 9 |
| Ovary | 6 | 0 | 135 | 28 | 1 | 1 | 2 | 81 | 6 | 449 | 2 | 0 | 51 | 26 | 2 | 0 | 0 | 120 |
| Pancreas | 13 | 0 | 2 | 0 | 10 | 7 | 8 | 0 | 76 | 0 | 629 | 0 | 15 | 0 | 7 | 160 | 14 | 40 |
| Serum | 0 | 201 | 0 | 1 | 0 | 0 | 0 | 0 | 0 | 0 | 1 | 847 | 0 | 0 | 0 | 0 | 0 | 0 |
| Skin | 3 | 0 | 14 | 9 | 3 | 1 | 49 | 132 | 0 | 0 | 49 | 0 | 697 | 0 | 2 | 4 | 0 | 30 |
| Spleen | 0 | 0 | 0 | 0 | 1 | 4 | 0 | 163 | 0 | 0 | 0 | 0 | 36 | 787 | 1 | 0 | 0 | 4 |
| Stomach | 149 | 0 | 1 | 14 | 154 | 8 | 2 | 0 | 0 | 2 | 5 | 0 | 3 | 0 | 565 | 0 | 0 | 11 |
| Testis | 0 | 0 | 17 | 15 | 6 | 1 | 0 | 0 | 0 | 0 | 196 | 0 | 8 | 1 | 3 | 801 | 0 | 13 |
| Thyroid | 0 | 0 | 0 | 0 | 0 | 0 | 0 | 0 | 0 | 0 | 0 | 0 | 0 | 0 | 0 | 0 | 990 | 0 |
| Uterus | 0 | 0 | 5 | 57 | 1 | 0 | 79 | 18 | 1 | 65 | 42 | 0 | 119 | 116 | 8 | 18 | 0 | 568 |

**b**

|  | Reference | | |  | |  | |  | |  | |  | |  | |  | |  | |  | |  | |  | |  | |  | |  | |  | |  |  |
| --- | --- | --- | --- | --- | --- | --- | --- | --- | --- | --- | --- | --- | --- | --- | --- | --- | --- | --- | --- | --- | --- | --- | --- | --- | --- | --- | --- | --- | --- | --- | --- | --- | --- | --- | --- |
|  | Brain | Exosome | Femur | | Heart | | Intestines | | Kidney | | Liver | | Lung | | Muscle | | Ovary | | Pancreas | | Serum | | Skin | | Spleen | | Stomach | | Testis | | Thyroid | | Uterus | | |
| Brain | 1006 | 0 | 0 | | 0 | | 0 | | 0 | | 0 | | 0 | | 0 | | 0 | | 60 | | 0 | | 0 | | 0 | | 0 | | 14 | | 0 | | 0 | | |
| Exosome | 0 | 1006 | 0 | | 0 | | 0 | | 0 | | 0 | | 0 | | 0 | | 0 | | 0 | | 0 | | 0 | | 0 | | 0 | | 0 | | 0 | | 0 | | |
| Femur | 0 | 0 | 994 | | 0 | | 0 | | 0 | | 0 | | 37 | | 0 | | 0 | | 0 | | 0 | | 0 | | 75 | | 0 | | 0 | | 175 | | 19 | | |
| Heart | 0 | 0 | 0 | | 866 | | 0 | | 0 | | 0 | | 0 | | 0 | | 307 | | 0 | | 0 | | 0 | | 0 | | 0 | | 0 | | 0 | | 0 | | |
| Intestines | 0 | 0 | 0 | | 0 | | 1006 | | 0 | | 0 | | 0 | | 0 | | 0 | | 0 | | 0 | | 0 | | 0 | | 0 | | 0 | | 0 | | 0 | | |
| Kidney | 0 | 0 | 0 | | 0 | | 0 | | 1006 | | 0 | | 0 | | 0 | | 0 | | 0 | | 0 | | 0 | | 0 | | 0 | | 0 | | 0 | | 0 | | |
| Liver | 0 | 0 | 7 | | 0 | | 0 | | 0 | | 1006 | | 0 | | 0 | | 0 | | 185 | | 0 | | 0 | | 0 | | 0 | | 0 | | 0 | | 211 | | |
| Lung | 0 | 0 | 0 | | 0 | | 0 | | 0 | | 0 | | 969 | | 0 | | 0 | | 0 | | 0 | | 0 | | 0 | | 0 | | 0 | | 0 | | 1 | | |
| Muscle | 0 | 0 | 5 | | 0 | | 0 | | 0 | | 0 | | 0 | | 1006 | | 0 | | 0 | | 0 | | 0 | | 0 | | 169 | | 0 | | 0 | | 0 | | |
| Ovary | 0 | 0 | 0 | | 140 | | 0 | | 0 | | 0 | | 0 | | 0 | | 694 | | 0 | | 0 | | 0 | | 0 | | 0 | | 0 | | 0 | | 150 | | |
| Pancreas | 0 | 0 | 0 | | 0 | | 0 | | 0 | | 0 | | 0 | | 0 | | 0 | | 760 | | 0 | | 0 | | 0 | | 0 | | 0 | | 0 | | 0 | | |
| Serum | 0 | 0 | 0 | | 0 | | 0 | | 0 | | 0 | | 0 | | 0 | | 0 | | 0 | | 1006 | | 0 | | 0 | | 0 | | 0 | | 0 | | 0 | | |
| Skin | 0 | 0 | 0 | | 0 | | 0 | | 0 | | 0 | | 0 | | 0 | | 0 | | 0 | | 0 | | 1006 | | 0 | | 17 | | 0 | | 0 | | 0 | | |
| Spleen | 0 | 0 | 0 | | 0 | | 0 | | 0 | | 0 | | 0 | | 0 | | 0 | | 0 | | 0 | | 0 | | 931 | | 31 | | 0 | | 0 | | 0 | | |
| Stomach | 0 | 0 | 0 | | 0 | | 0 | | 0 | | 0 | | 0 | | 0 | | 0 | | 1 | | 0 | | 0 | | 0 | | 789 | | 0 | | 0 | | 0 | | |
| Testis | 0 | 0 | 0 | | 0 | | 0 | | 0 | | 0 | | 0 | | 0 | | 0 | | 0 | | 0 | | 0 | | 0 | | 0 | | 992 | | 0 | | 0 | | |
| Thyroid | 0 | 0 | 0 | | 0 | | 0 | | 0 | | 0 | | 0 | | 0 | | 0 | | 0 | | 0 | | 0 | | 0 | | 0 | | 0 | | 831 | | 0 | | |
| Uterus | 0 | 0 | 0 | | 0 | | 0 | | 0 | | 0 | | 0 | | 0 | | 5 | | 0 | | 0 | | 0 | | 0 | | 0 | | 0 | | 0 | | 625 | | |

**c**

|  | Reference | |  |  |  |  |  |  |  |  |  |  |  |  |  |  |  |  |
| --- | --- | --- | --- | --- | --- | --- | --- | --- | --- | --- | --- | --- | --- | --- | --- | --- | --- | --- |
| Prediction | Brain | Exosome | Femur | Heart | Intestines | Kidney | Liver | Lung | Muscle | Ovary | Pancreas | Serum | Skin | Spleen | Stomach | Testis | Thyroid | Uterus |
| Brain | 1006 | 0 | 0 | 0 | 0 | 0 | 0 | 0 | 0 | 0 | 60 | 0 | 0 | 0 | 0 | 14 | 0 | 0 |
| Exosome | 0 | 1006 | 0 | 0 | 0 | 0 | 0 | 0 | 0 | 0 | 0 | 0 | 0 | 0 | 0 | 0 | 0 | 0 |
| Femur | 0 | 0 | 994 | 0 | 0 | 0 | 0 | 37 | 0 | 0 | 0 | 0 | 0 | 75 | 0 | 0 | 175 | 19 |
| Heart | 0 | 0 | 0 | 866 | 0 | 0 | 0 | 0 | 0 | 307 | 0 | 0 | 0 | 0 | 0 | 0 | 0 | 0 |
| Intestines | 0 | 0 | 0 | 0 | 1006 | 0 | 0 | 0 | 0 | 0 | 0 | 0 | 0 | 0 | 0 | 0 | 0 | 0 |
| Kidney | 0 | 0 | 0 | 0 | 0 | 1006 | 0 | 0 | 0 | 0 | 0 | 0 | 0 | 0 | 0 | 0 | 0 | 0 |
| Liver | 0 | 0 | 7 | 0 | 0 | 0 | 1006 | 0 | 0 | 0 | 185 | 0 | 0 | 0 | 0 | 0 | 0 | 211 |
| Lung | 0 | 0 | 0 | 0 | 0 | 0 | 0 | 969 | 0 | 0 | 0 | 0 | 0 | 0 | 0 | 0 | 0 | 1 |
| Muscle | 0 | 0 | 5 | 0 | 0 | 0 | 0 | 0 | 1006 | 0 | 0 | 0 | 0 | 0 | 169 | 0 | 0 | 0 |
| Ovary | 0 | 0 | 0 | 140 | 0 | 0 | 0 | 0 | 0 | 694 | 0 | 0 | 0 | 0 | 0 | 0 | 0 | 150 |
| Pancreas | 0 | 0 | 0 | 0 | 0 | 0 | 0 | 0 | 0 | 0 | 760 | 0 | 0 | 0 | 0 | 0 | 0 | 0 |
| Serum | 0 | 0 | 0 | 0 | 0 | 0 | 0 | 0 | 0 | 0 | 0 | 1006 | 0 | 0 | 0 | 0 | 0 | 0 |
| Skin | 0 | 0 | 0 | 0 | 0 | 0 | 0 | 0 | 0 | 0 | 0 | 0 | 1006 | 0 | 17 | 0 | 0 | 0 |
| Spleen | 0 | 0 | 0 | 0 | 0 | 0 | 0 | 0 | 0 | 0 | 0 | 0 | 0 | 931 | 31 | 0 | 0 | 0 |
| Stomach | 0 | 0 | 0 | 0 | 0 | 0 | 0 | 0 | 0 | 0 | 1 | 0 | 0 | 0 | 789 | 0 | 0 | 0 |
| Testis | 0 | 0 | 0 | 0 | 0 | 0 | 0 | 0 | 0 | 0 | 0 | 0 | 0 | 0 | 0 | 992 | 0 | 0 |
| Thyroid | 0 | 0 | 0 | 0 | 0 | 0 | 0 | 0 | 0 | 0 | 0 | 0 | 0 | 0 | 0 | 0 | 831 | 0 |
| Uterus | 0 | 0 | 0 | 0 | 0 | 0 | 0 | 0 | 0 | 5 | 0 | 0 | 0 | 0 | 0 | 0 | 0 | 625 |

**d**

|  | Reference | |  |  |  |  |  |  |  |  |  |  |  |  |  |  |  |  |
| --- | --- | --- | --- | --- | --- | --- | --- | --- | --- | --- | --- | --- | --- | --- | --- | --- | --- | --- |
|  | Brain | Exosome | Femur | Heart | Intestines | Kidney | Liver | Lung | Muscle | Ovary | Pancreas | Serum | Skin | Spleen | Stomach | Testis | Thyroid | Uterus |
| Brain | 384 | 45 | 0 | 3 | 37 | 0 | 1 | 0 | 0 | 0 | 21 | 4 | 3 | 1 | 25 | 0 | 13 | 5 |
| Exosome | 39 | 800 | 0 | 0 | 115 | 0 | 0 | 0 | 0 | 0 | 36 | 3 | 1 | 0 | 32 | 0 | 42 | 3 |
| Femur | 0 | 0 | 518 | 3 | 0 | 198 | 0 | 0 | 1 | 3 | 0 | 0 | 0 | 0 | 0 | 0 | 0 | 0 |
| Heart | 387 | 0 | 447 | 990 | 0 | 0 | 222 | 0 | 208 | 27 | 0 | 0 | 0 | 0 | 0 | 0 | 0 | 0 |
| Intestines | 131 | 124 | 0 | 0 | 814 | 0 | 0 | 2 | 0 | 0 | 124 | 93 | 28 | 1 | 139 | 0 | 129 | 22 |
| Kidney | 0 | 0 | 0 | 0 | 0 | 805 | 0 | 0 | 0 | 0 | 0 | 0 | 0 | 0 | 0 | 0 | 0 | 0 |
| Liver | 29 | 0 | 0 | 5 | 0 | 0 | 575 | 0 | 2 | 0 | 0 | 0 | 0 | 0 | 0 | 0 | 0 | 0 |
| Lung | 9 | 5 | 0 | 0 | 9 | 0 | 0 | 788 | 0 | 0 | 22 | 2 | 2 | 21 | 4 | 0 | 5 | 2 |
| Muscle | 0 | 0 | 36 | 5 | 0 | 0 | 207 | 0 | 795 | 145 | 194 | 0 | 29 | 0 | 0 | 0 | 0 | 0 |
| Ovary | 0 | 0 | 2 | 0 | 0 | 0 | 0 | 201 | 0 | 779 | 0 | 0 | 180 | 0 | 0 | 0 | 0 | 163 |
| Pancreas | 18 | 11 | 1 | 0 | 16 | 1 | 1 | 1 | 0 | 0 | 553 | 1 | 3 | 3 | 15 | 3 | 1 | 3 |
| Serum | 0 | 0 | 0 | 0 | 1 | 0 | 0 | 0 | 0 | 0 | 1 | 880 | 0 | 0 | 0 | 0 | 0 | 0 |
| Skin | 7 | 15 | 1 | 0 | 9 | 0 | 0 | 7 | 0 | 52 | 49 | 10 | 757 | 3 | 7 | 0 | 5 | 8 |
| Spleen | 0 | 0 | 0 | 0 | 0 | 0 | 0 | 0 | 0 | 0 | 0 | 0 | 0 | 974 | 0 | 0 | 0 | 0 |
| Stomach | 2 | 4 | 0 | 0 | 4 | 0 | 0 | 0 | 0 | 0 | 2 | 12 | 1 | 0 | 781 | 0 | 2 | 12 |
| Testis | 0 | 0 | 0 | 0 | 0 | 0 | 0 | 0 | 0 | 0 | 0 | 0 | 0 | 0 | 0 | 998 | 0 | 0 |
| Thyroid | 0 | 2 | 1 | 0 | 1 | 2 | 0 | 1 | 0 | 0 | 4 | 1 | 2 | 3 | 3 | 5 | 809 | 2 |
| Uterus | 0 | 0 | 0 | 0 | 0 | 0 | 0 | 6 | 0 | 0 | 0 | 0 | 0 | 0 | 0 | 0 | 0 | 786 |

.

**a**

**Figure S1**. Characterization of serum-derived exosomes. (a) The diameters of exosomes derived from the serum of Slc:ddY mice. (b) The western blot analysis by using anti-CD9 (upper) and anti-CD81 mAbs. The lane 1: mouse serum, lane 2: exosome standard obtained from human A549 cell culture supernatant, lane 3: exosome standard obtained from mouse B16F10 cell culture media, lane 4: exosomes derived from sera of Slc:ddY mice in this study, lane M: marker (kDa).


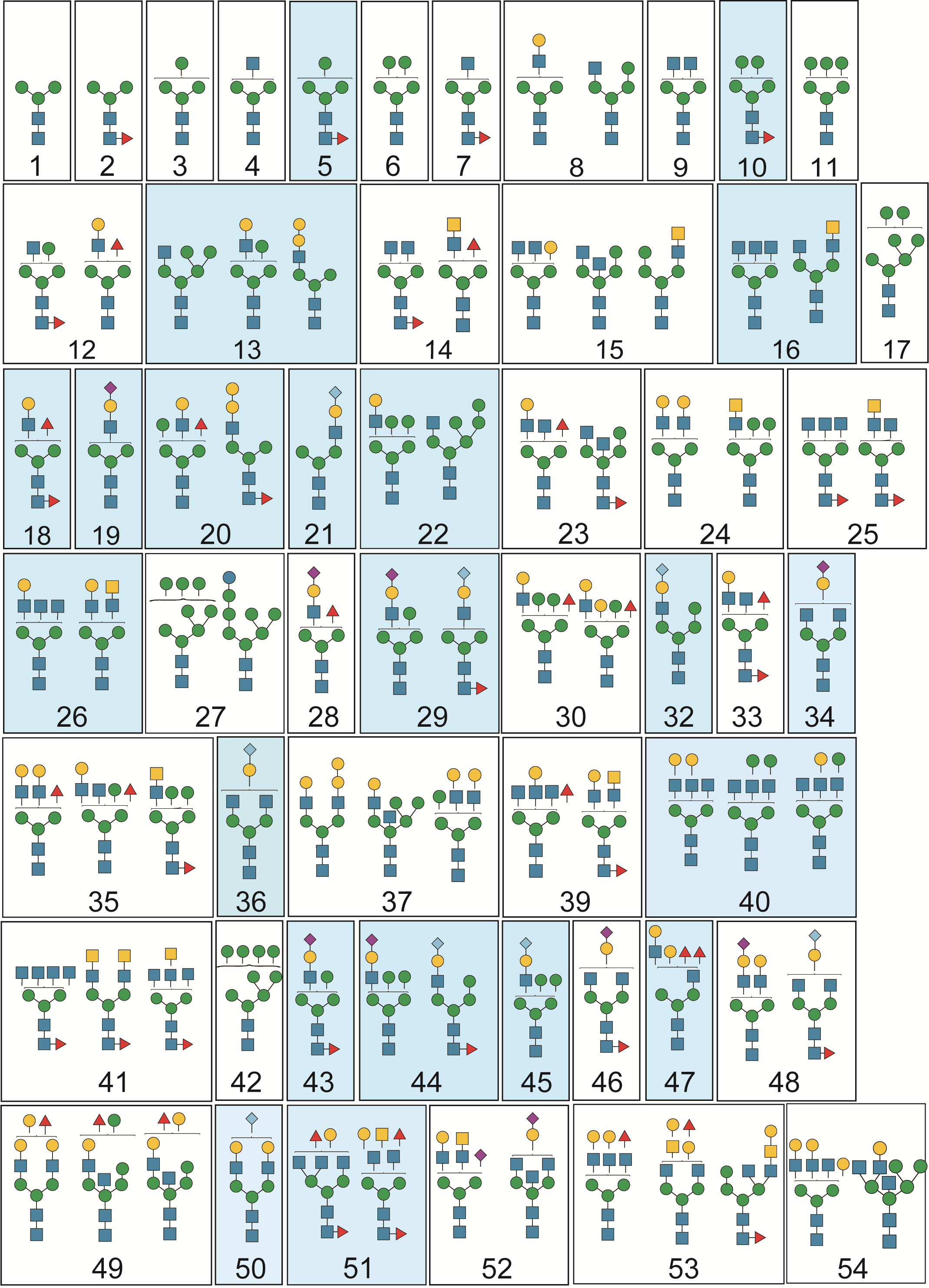

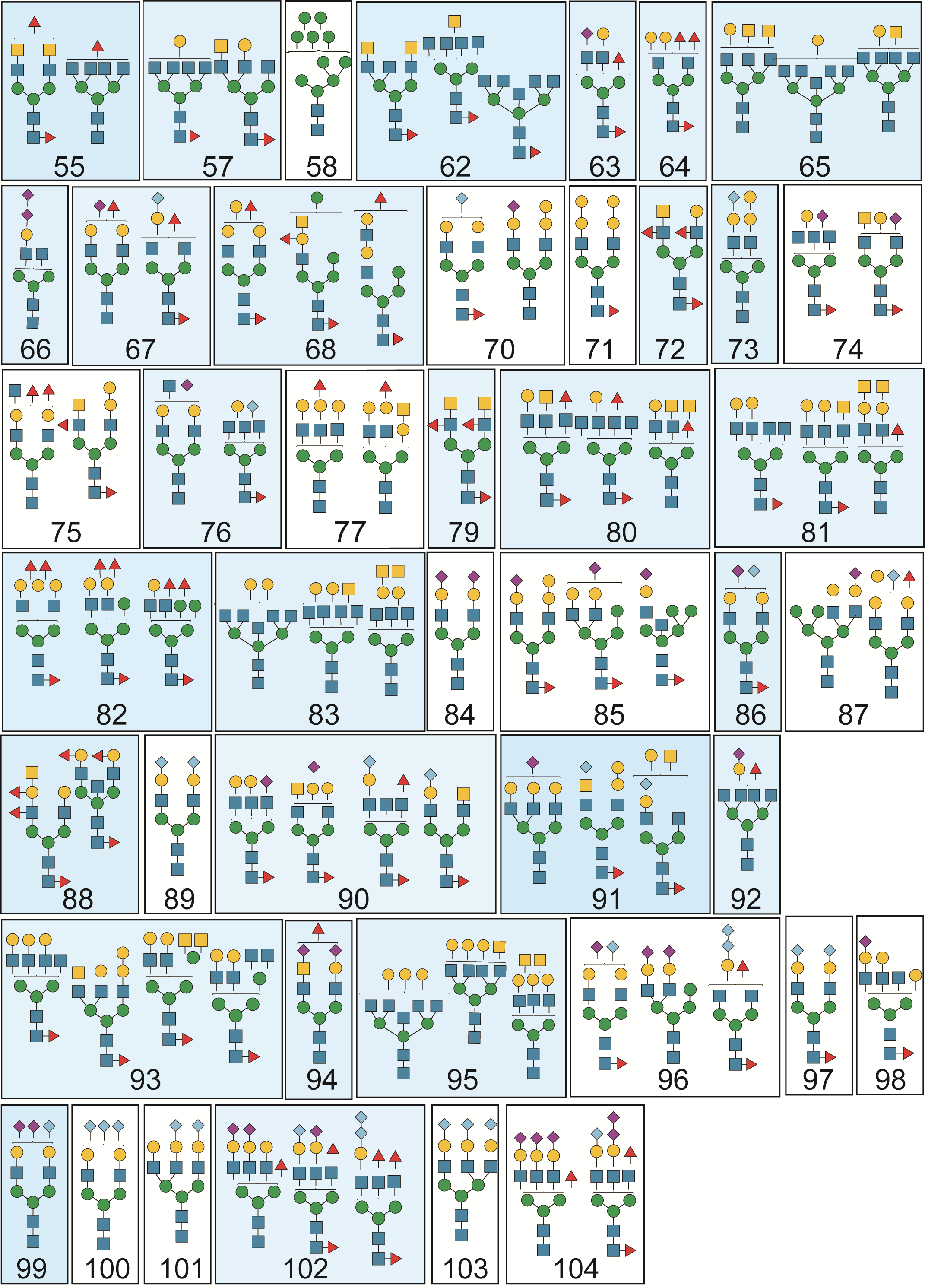


**Figure S2**. Predicted chemical structures of *N*-glycans (glycoforms) listed in Table S1. These glycoforms were predicted by using the reported data of the mammalian *N*-glycans in Expasy GlyConnect (<https://glyconnect.expasy.org/>). Glycoforms in the blue-colored boxes were identified for the first-time in mouse.

**Figure S3**. MALDI-TOFMS spectra of all *N*-glycan samples used in this study: (a) brain, (b) femur, (c) heart, (d) intestines, (e) kidney, (f) liver, (g) lung, (h) muscle, (i) ovary, (j) pancreas, (k) skin, (l) spleen, (m) stomach, (n) testis, (o) thyroid, (p) uterus, (q) serum, (r) exosomes. I.S. indicates the internal standard (predicted *m/z* = 2477.95).
